# Indeterminate results of interferon gamma release assays in the screening of latent tuberculosis infection: a systematic review and meta-analysis

**DOI:** 10.3389/fimmu.2023.1170579

**Published:** 2023-05-15

**Authors:** Guozhong Zhou, Qingyi Luo, Shiqi Luo, Hongbo Chen, Shunli Cai, Xin Guo, Jian He, Yuan Xia, Hanse Li, Yingchen Zhou, Yazhou Zhang, Chao Song

**Affiliations:** ^1^ Department of Science and Research, Anning First People’s Hospital Affiliated to Kunming University of Science and Technology, Kunming, Yunnan, China; ^2^ Department of Medical Imaging, Yanan Hospital Affiliated to Kunming Medical University, Kunming, Yunnan, China; ^3^ Department of Immunology, Institute of Basic Medical Sciences Chinese Academy of Medical Sciences, School of Basic Medicine Peking Union Medical College, Beijing, China; ^4^ Department of Pulmonary and Critical Care Medicine, Anning First People’s Hospital Affiliated to Kunming University of Science and Technology, Kunming, Yunnan, China; ^5^ School of Basic Medical Sciences, Kunming Medical University, Kunming, Yunnan, China; ^6^ The School of Medicine, Kunming University, Kunming, China; ^7^ Department of Gerontology 2, The Second People’s Hospital of Kunming, Kunming, China; ^8^ Department of Medical Imaging, Anning First People’s Hospital Affiliated to Kunming University of Science and Technology, Kunming, Yunnan, China

**Keywords:** interferon gamma release assay (IGRA), latent tuberculosis infection (LTBI), indeterminate, diagnosis, meta-analysis

## Abstract

**Objectives:**

We aimed to evaluate the indeterminate rate of interferon gamma release assays (IGRAs) in the detection of latent tuberculosis infection (LTBI).

**Methods:**

On 15 November 2022, we searched the PubMed® (National Library of Medicine, Bethesda, MD, USA), Embase® (Elsevier, Amsterdam, the Netherlands), and Cochrane Library databases in accordance with the Preferred Reporting Items for Systematic Reviews and Meta-Analyses (PRISMA) guidelines. Two investigators independently extracted the study data and assessed their quality using a modified quality assessment of diagnostic accuracy studies (i.e., QUADAS-2) tool. A random-effects model was used to calculate pooled results.

**Results:**

We included 403 studies involving 486,886 individuals and found that the pooled indeterminate rate was 3.9% (95% CI 3.5%–4.2%). The pooled indeterminate rate for QuantiFERON®-TB (QFT) was similar to that for T-SPOT®.TB (T-SPOT) [odds ratio (OR) = 0.88, 95% CI 0.59–1.32]; however, the indeterminate rate for a new generation of QFT (QFT-plus) was lower than that of T-SPOT (OR = 0.24, 95% CI 0.16–0.35). The indeterminate rate in the immunocompromised population was significantly higher than that in healthy controls (OR = 3.51, 95% CI 2.11–5.82), and it increased with the reduction of CD4+ cell count in HIV-positive patients. Children’s pooled indeterminate rates (OR = 2.56, 95% CI 1.79–3.57) were significantly higher than those of adults, and the rates increased as the children’s age decreased.

**Conclusion:**

On average, 1 in 26 tests yields indeterminate IGRA results in LTBI screening. The use of advanced versions of the QuantiFERON-TB assay (QFT-plus), may potentially reduce the occurrence of an indeterminate result. Our study emphasizes the high risk of immunosuppression and young age in relation to indeterminate IGRA, which should receive more attention in the management of LTBI.

**Systematic review registration:**

PROSPERO https://www.crd.york.ac.uk/prospero/display_record.php?ID=CRD42020211363, CRD42020211363.

## 1 Introduction

Latent tuberculosis infection (LTBI) is defined as a persistent immune response to *Mycobacterium tuberculosis* antigen stimulation, with no evidence of clinically manifested active tuberculosis (1, 2). Globally, approximately one-quarter of the population is affected by LTBI (3). The diagnosis and preventive treatment of LTBI are critical for TB elimination (4). Two immune-based tests, that is, the interferon gamma (interferon-γ) release assay (IGRA) and the tuberculin skin test (TST), are currently used to diagnose LTBI (5, 6).

Bacillus Calmette–Guérin (BCG) and non-tuberculosis mycobacteria (NTM) have no effect on IGRAs, which assess the level of interferon-γ responses to the TB-specific antigens early secreted antigenic target 6 kDa (ESAT-6) and culture filtrate protein-10 kDa (CFP-10) (7). Two types of IGRA are now commercially available, the T-SPOT®.TB (T-SPOT, Oxford Immunotec Ltd, Oxford, UK) and QuantiFERON®-TB (QFT, Cellestis Ltd., Carnegie, Australia), which use enzyme-linked immunosorbent spot (ELISPOT) and enzyme-linked immunosorbent assay (ELISA) formats respectively (8). A mitogen tube is used as positive control to assess the performance of the test and the functionality of the individual's T-cells in both types of commercially available IGRAs, and a nil tube is used as a negative control to adjust for background interferon-γ. Either high interferon-γ levels in the negative control or a low response in the positive control can result in an indeterminate result (9).

Previous meta-analyses have evaluated the indeterminate rate of IGRAs. Two studies assessed the indeterminate rate in HIV-positive patients (10, 11), two studies assessed the indeterminate rate in inflammatory bowel disease patients (12, 13), and one study assessed the indeterminate rate in patients that underwent an organ transplant (14). Two large meta-analyses assessed the indeterminate rate in the entire population (15, 16); however, active TB, suspected TB, and LTBI were included. As the immune response may differ depending on the type of TB infection, the indeterminate rate of IGRAs may vary among these groups. For example, Santin et al.’s meta-analysis, focusing on HIV-positive patients, found that the difference in indeterminate rates for QuantiFERON-TB Gold In-Tube (QFT-GIT) in active TB, suspected TB, and LTBI, were 15.3% (95% CI 10.8%−21.2%), 12.3% (95% CI 6.9%−39.4%), and 3.9% (95% CI 2.4%−6.4%), respectively (11). However, to our knowledge, no meta-analysis has comprehensively assessed the indeterminate rate of IGRAs specifically in the detection of LTBI. Therefore, we included 403 studies and conducted a systematic review and meta-analysis to evaluate the indeterminate rate of IGRAs in the screening of LTBI.

## 2 Methods

We strictly adhered to the standards of the Preferred Reporting Items for Systematic Reviews and Meta-Analyses (PRISMA) in reporting the findings of this review (17). The study is registered in PROSPERO as CRD42020211363; however, the protocol was greatly modified before it was implemented. The adjusted protocol is shown in the **Appendix**, page 67.

### 2.1 Search strategy and selection criteria

In this systematic review and meta-analysis, we searched the PubMed, Cochrane Library, and Embase® (Elsevier, Amsterdam, the Netherlands) databases on 15 November 2022, with no language or time constraints. **Table S1** in the appendix shows the keywords. The reference lists of relevant articles were manually checked for other potentially relevant papers.

Studies were included if they met all of the following criteria (1): they screened for LTBI in healthy or high-risk adults and/or children (2); IGRAs were used to detect LTBI, with initial indeterminate results reported; and (3) they were cross-sectional or longitudinal studies. Referring to Campbell et al.’s study (18) and our previous study (19), **Table S2** in the appendix provides the classification of the high-risk population, which accounted for recent contacts, populations with the possibility of contact, immunocompromised populations, and populations with the possibility of immunosuppression.

Studies were excluded based on the following criteria (1): individuals who were active TB and suspected active TB were not excluded at baseline (2); only IGRA- or TST-positive/-negative individuals were included at baseline (3); non-commercial or modified IGRAs were used (4); they included invalid results caused by technical errors, including insufficient cells or blood, machine failure, blood contamination, or prolonged incubation time that exceeded the manufacturer’s recommendation; and (5) they were abstracts, letters, case reports, or reviews.

Two investigators (GZZ and QYL) independently screened the article titles and abstracts retrieved from the literature search. The full texts of the potentially eligible studies were further reviewed before being included in the analysis. A third investigator (CS) cross-checked the extracted data. Disagreements were resolved through consensus.

### 2.2 Data extraction and quality assessment

Using a preconceived and standardized data extraction form, we collected information including the first author’s name, year of publication, title, study area, study design, the timing of data collection (prospective or retrospective), investigated population, participant demographics, IGRA type and manufacturer, number of individuals screened, and number of individuals with initial indeterminate results. Two investigators (GZZ and QYL) independently extracted data from individual studies. The third investigator (CS) cross-checked extracted data. Disagreements were resolved through consensus.

There is currently no reference diagnostic test for LTBI. Furthermore, the Quality Assessment of Diagnostic Accuracy Studies 2 (QUADAS-2) tool indicated that some items would not be appropriate for inclusion in this study. Therefore, with reference to previous studies (18–20), the QUADAS-2 tool was modified with six quality items to improve the assessment of study diagnostic accuracy. High-quality studies were defined as those that met at least five of the criteria, those of moderate quality met three or four criteria, and those of low quality met two or fewer criteria (Appendix **Table S3**). Two investigators (GZZ and QYL) independently assessed the methodological quality of one-quarter of the studies. A third investigator (CS) independently reviewed those assessments. Disagreements were resolved by consensus.

### 2.3 Statistical analysis

A random-effects model was used to calculate the pooled prevalence rates and risks, and their 95% CIs. A random-effects metaregression was used to examine the factors that influenced the pooled prevalence rates. The *I*²-statistic was used to assess the heterogeneity of the included studies (21), with *I*² > 50% indicating significant heterogeneity. Publication bias was not statistically calculated because factors other than results, including investigator motivation and funding, may influence the publication of a study (18, 22), and publication bias is only one possible explanation of funnel plot asymmetry (23, 24). The “meta” package in R statistical software, version 3.4.3 (Schwarzer, 2007; Team, 2017), was used to conduct the meta-analysis.

## 3 Results

### 3.1 Characteristics of the included studies

**Figure 1:**
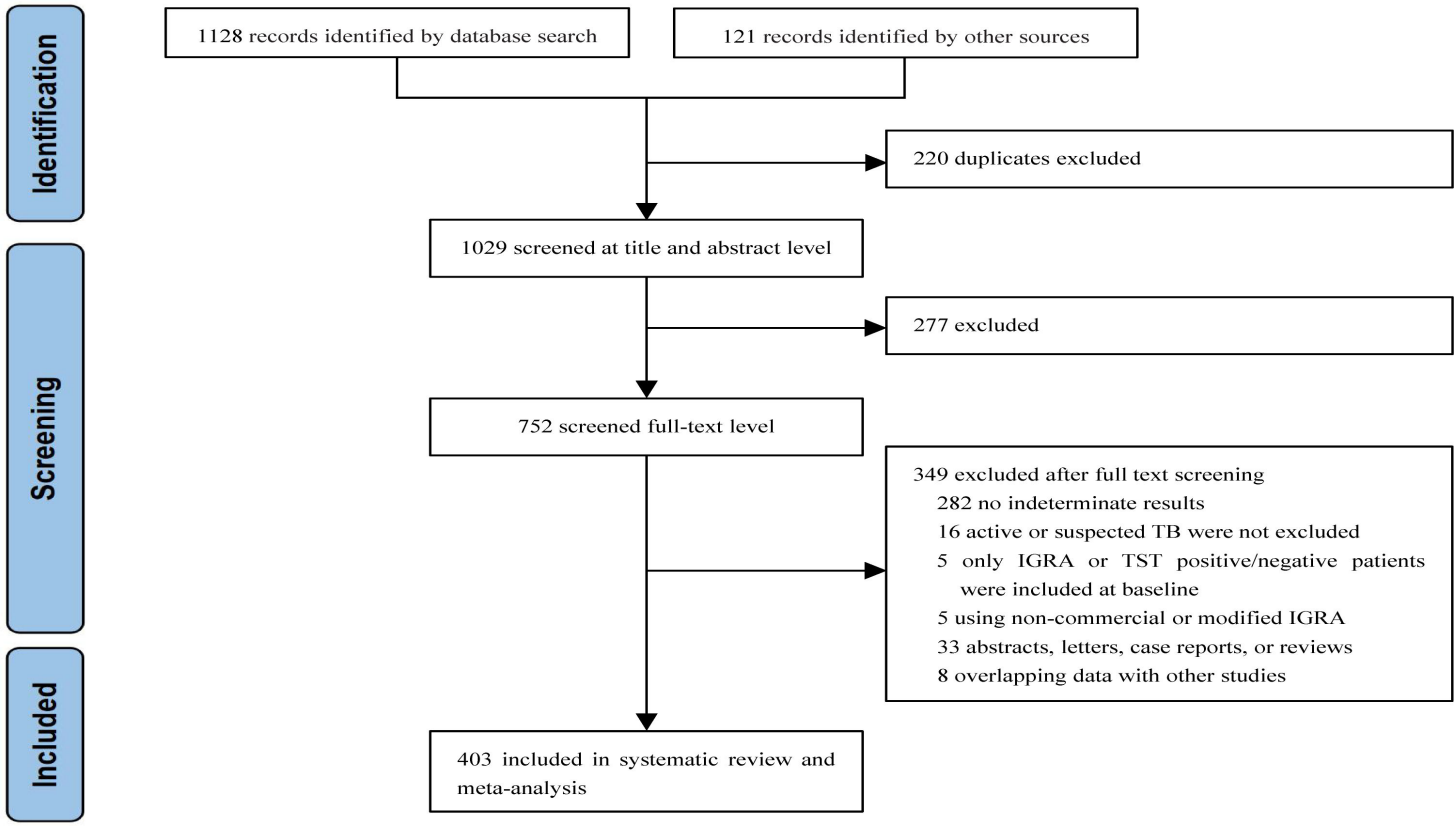
Study selection.

We identified 1,249 articles after removing duplicates. Of these, 277 articles were excluded after reviewing the titles and abstracts. The full texts of 752 articles were assessed. Finally, 403 studies involving 486,886 individuals were included in the analyses (**Figure 1**).

Of the 403 studies, 358 were from areas with a TB burden of less than 100 cases per 100,000 people, whereas 45 studies were from areas with a TB burden of more than 100 cases per 100,000 people. In addition, 183 studies were cohort studies, 125 were cross-sectional studies, 4 studies were randomized controlled trials, and 91 studies did not state the study type. Detailed characteristics of study participants can be found in Appendix **Table S4**.

Of the studies analyzed, 315 and 53 were considered to be of high and moderate quality, respectively. The main limitation found was that the definition of indeterminate for IGRA was not reported in 65 (16.1%) studies, which poses a potential risk of inconsistent definitions of indeterminate IGRA results. In addition, the reason for participants’ withdrawal from the study was not reported in 55 (13.6%) studies. See Appendix **Table S5** for details.

### 3.2 Comparison of IGRA indeterminate rates among different populations

Our calculation of the pooled indeterminate rate in all populations showed an overall rate of 3.9% (95% CI 3.5%–4.2%; *I* ^2^ = 97%). Subgroup analysis showed that immunity status significantly affected indeterminate results. The pooled indeterminate rate in the immunocompromised population (5.7%, 95% CI 4.8%–6.6%; *I* ^2^ = 94%) was significantly higher than in the immunocompetent population (1.9%, 95% CI 1.5%–2.3%; *I* ^2^ = 97%). In the population with a possibility of immunosuppression, the pooled indeterminate rate was 4.8% (95% CI 4.1%–5.6%; *I* ^2^ = 97%) (see **Table 1**).

**Table 1:** Comparison of interferon gamma release assay (IGRA) indeterminate rates among different populations.

	Studies (*n*)	Participants (*n*)	Indeterminate rate, % (95% CI)	*I^2^* (%)
All populations	432	486,886	3.9 (3.5–4.2)	97
Immunocompetent population	137	199,822	1.9 (1.5–2.3)	97
Healthy people^*^	40	115,057	1.8 (1.2–2.6)	98
Recent contacts	48	26,487	2.1 (1.4–2.9)	94
HCWs	41	43,814	1.6 (1.0–2.4)	97
Immigrants or refugees	7	5,769	2.9 (0.5–7.3)	98
Contacts and immigrants	1	8,698	1.9 (1.6–2.2)	–
Immunocompromised patients (all)	134	48,379	5.7 (4.8–6.6)	94
Immunocompromised patients^#^	15	6,201	8.6 (5.9–11.9)	94
HIV-positive	55	24,625	4.3 (3.2–5.6)	95
Hemodialysis	22	6,813	5.3 (3.9–7.0)	85
Transplant recipients	29	9,255	7.0 (5.6–8.7)	87
Cancer	7	937	8.5 (3.3–15.9)	91
Drug and alcohol abusers	3	548	2.5 (1.4–4.0)	0
With possibility of immunosuppression^$^	154	109,441	4.8 (4.1–5.6)	97
IMID patients	106	32,147	5.1 (4.2–6.1)	93
Prisoners	2	35,502	2.2 (0.0–9.4)	98
Children	31	34,351	4.3 (2.9–5.9)	98
Young children	8	1,893	4.7 (2.9–6.9)	74
Pregnant women	4	1,376	6.0 (4.7–7.4)	5
Diabetes	2	3,943	5.9 (2.7–10.2)	92
Nursing house residents	1	229	0.4 (0.0–1.7)	–
High risk (combined)	7	129,244	1.9 (1.0–3.1)	99

*Healthy people included general populations, army recruits, and healthy controls for high-risk populations in included studies. ^#^The target populations of these studies were a mixed group of immunocompromised individuals, including HIV-positive patients, cancer patients, hemodialysis patients, and/or transplant recipients. ^$^Referring to the previous study (18, 19), we classified IMID patients, prisoners, children, pregnant women, diabetes patients, and nursing home residents as populations with a possibility of immunosuppression. HCWs, health care workers; IMID, immune-mediated inflammatory disease.

Appendix **Table S6** shows that in multivariable models of metaregression, the population (i.e., immunity status), the timing of data collection, age, proportion of men, the type of IGRA, area, and the number of participants were all significantly associated with the pooled indeterminate rate (*p* < 0.05). We performed subgroup analyses stratified by the year of publication, type of study, timing of data collection, TB burden of the areas, age group, proportion of men, number of participants, type of IGRA, and quality of included studies (Appendix **Table S7**).

### 3.3 Comparison of IGRA indeterminate rates between QFT and T-SPOT in head-to-head studies

**Figure 2:**
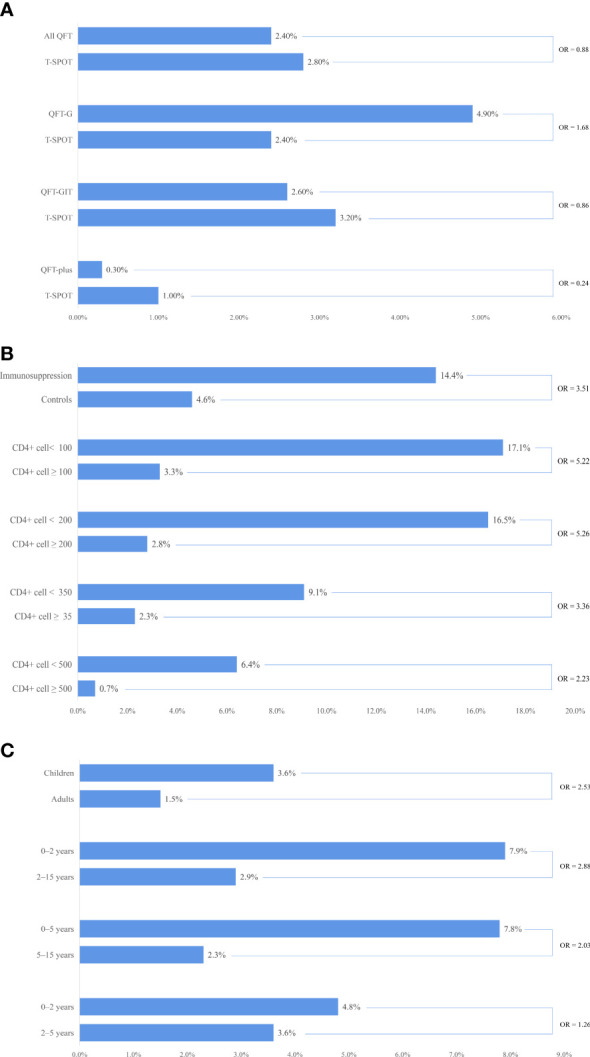
Comparisons of indeterminate interferon gamma release assay (IGRA) rates: immune status, age groups, and test types. **(A)** comparison of indeterminate IGRA rates between QuantiFERON®-TB (QFT) and T-SPOT®.TB (T-SPOT) in head-to-head studies; **(B)** comparison of indeterminate IGRA rates among different immune statuses; **(C)** comparison of indeterminate IGRA rates among different age groups.

We compared the indeterminate rates in 55 head-to-head studies and found that the indeterminate rate for QFT was similar to that for T-SPOT (pooled OR = 0.88, 95% CI 0.59%–1.32%; *I* ^2^ = 91%). However, the subgroup analysis stratified by the generation of QFT showed differences in indeterminate rate. Although not reaching statistical significance, we observed that the indeterminate rate for the second generation of QFT (QFT-G) was higher than that for T-SPOT (pooled OR = 1.68, 95% CI 0.76–3.70; *I* ^2^ = 39%). The indeterminate rate for the third generation of QFT (QFT-GIT) was similar to that for T-SPOT (pooled OR = 0.86, 95% CI 0.54–1.37; *I* ^2^ = 93%). The fourth generation of QFT (QFT-plus) had a significantly lower indeterminate rate than that for T-SPOT (pooled OR = 0.24, 95% CI 0.16–0.35; *I* ^2^ = 0%) (see **Figure 2A**, and Appendix **Table S8** and **Figure S1**).

We compared the indeterminate rate for QFT in head-to-head studies among different generations. Although not reaching statistical significance, we observed that the indeterminate rate for QFT-GIT was lower than that for QFT-G (pooled OR = 0.64, 95% CI 0.23–1.75; *I* ^2^ = 62%), but that the indeterminate rate for QFT-GIT was higher than that for QFT-plus (pooled OR = 1.49, 0.98–2.17; *I* ^2^ = 0%) (see Appendix **Table S9**).

### 3.4 Comparison of IGRA indeterminate rates among different immune statuses

We compared the indeterminate rate between the immunocompromised population and their healthy controls in 16 studies and found that the indeterminate rate for the immunocompromised population was significantly higher than that for the healthy control (pooled OR = 3.51, 95% CI 2.11–5.82; *I* ^2^ = 61%). We further compared the indeterminate rate in HIV-positive patients among different groups stratified by CD4+ cell count. The indeterminate rate for the group with a CD4+ cell count lower than 100 cells/mm^3^ was significantly higher than that for the group with a CD4+ cell count greater than 100 cells/mm^3^ (pooled OR = 5.22, 95% CI 2.66–10.25; *I* ^2^ = 52%). A similar result was found in other subgroup analyses stratified by CD4+ cell count (see Table 3). We also found that the indeterminate rate decreased with the increase of CD4+ cell count (see **Figure 2B**, Appendix **Tables S10–11** and **Figures S2–S4**).

### 3.5 Comparison of indeterminate rates of IGRA among different age groups

We compared the indeterminate rate between children and adults in seven studies and found that the indeterminate rate for children was significantly higher than that for adults (pooled OR = 2.56, 95% CI 1.79–3.57; *I* ^2^ = 41%) (see Appendix **Figure S5**). We further compared the indeterminate rate in children among different age groups. The indeterminate rate for groups aged less than 2 years was significantly higher than that for groups aged 2–15 years (pooled OR = 2.88, 95% CI 1.70–4.87; *I* ^2^ = 44%). A similar result was found in other subgroup analyses stratified by age (see Table 4). We also found that the indeterminate rate decreased with an increase in age in children (see **Figure 2C**, and Appendix **Table S12** and **Figure S6**).

### 3.6 Comparison of indeterminate rates of IGRA caused by failed positive and failed negative controls among different populations

Finally, we compared the indeterminate rate of IGRA caused by failed positive and negative controls among the different populations. In all populations, the proportion of failed positive controls in indeterminate cases was 94.6% (95% CI 89.6%–98.0%; *I* ^2^ = 95%) and the proportion of failed negative controls was 4.0% (95% CI 1.4%–12.4%; *I* ^2^ = 93%). In the immunocompetent population, the proportion of failed positive controls in indeterminate cases was 99.4% (95% CI 97.3%–100%; *I* ^2^ = 51%) and the proportion of failed negative controls was 1.0% (95% CI 0.0%–3.5%; *I* ^2^ = 52%).

Furthermore, in immunocompromised patients, the proportion of failed positive controls in indeterminate cases was 95.7% (95% CI 85.6%–99.9%; *I* ^2^ = 95%) and the proportion of failed negative controls was 2.6% (95% CI 0.2%–7.2%; *I* ^2^ = 72%). In the population with a possibility of immunosuppression, the proportion of failed positive controls in indeterminate cases was 91.7% (95% CI 83.2%–97.5%; *I* ^2^ = 93%) and the proportion of failed negative controls was 5.6% (95% CI 1.4%–12.4%; *I* ^2^ = 90%) (see **Table 2**). For more details, see Appendix **Table S13**.

**Table 2:** Comparison of indeterminate rates of interferon gamma release assay (IGRA) caused by failed positive controls and failed negative controls among different populations.

	Studies (*n*)	Participants (*n*)	Indeterminate rate, % (95% CI)	*I^2^* (%)
Failed positive control				
All populations	71	2,599	94.6 (89.6–98.0)	95
Immunocompetent population^*^	13	430	99.4 (97.3–100.0)	51
Immunocompromised patients^#^	25	588	95.7 (85.6–99.9)	95
With possibility of immunosuppression^$^	30	939	91.7 (83.2–97.5)	93
Failed negative control				
All populations	66	2,317	4.0 (1.4–12.4)	93
Immunocompetent population^*^	13	430	1.0 (0.0–3.5)	52
Immunocompromised patients^#^	23	343	2.6 (0.2–7.2)	72
With possibility of immunosuppression^$^	27	902	5.6 (1.4–12.4)	90

*Immunocompetent population included healthy people, recent contacts, and populations with the possibility of contact. For further details, please refer to **Table S2** in the appendix. ^#^Immunocompromised individuals included HIV-positive patients, cancer patients, hemodialysis patients, and transplant recipients. ^$^Populations with a possibility of immunosuppression included IMID patients, prisoners, children, pregnant women, diabetes patients, and nursing home residents. Abbreviations: IMID, immune-mediated inflammatory disease.

## 4 Discussion

To our knowledge, our meta-analysis of 403 studies involving 486,886 participants is the largest to systematically assess the indeterminate rate of IGRAs in the screening for LTBI. Our study revealed five main findings. First, the pooled indeterminate rate of IGRAs was 3.9% in all populations. Second, the indeterminate rate for QFT was similar to that for T-SPOT, but the indeterminate rate for a new generation of QFT (QFT-plus) was significantly lower than that for T-SPOT. Third, the indeterminate rate for the immunocompromised population was significantly higher than that for healthy controls, and the indeterminate rate increased as CD4+ cell count decreased in HIV-positive patients. Fourth, the indeterminate rate for children was significantly higher than that of adults, and the indeterminate rate in children increased as age decreased. Fifth, 94.6% of indeterminate cases were caused by a failed positive control.

The End TB Strategy of the World Health Organization (WHO) recommends that all countries should aim to provide preventive treatment for LTBI for > 90% of people living with HIV and living with children who are contacts of TB cases by 2025 (25), which means that a large number of people worldwide require screening for LTBI. Although our study reveals a low indeterminate rate of 3.9%, it is estimated that hundreds of thousands of IGRAs might not produce a conclusive result in the screening of LTBI. Indeterminate results pose diagnostic challenges for healthcare providers, in addition to causing frustration for individuals undergoing testing, particularly when determining whether or not to initiate preventive treatment, as this therapy has toxic side effects (26). Similarly, indeterminate IGRA results have significant consequences for individual patients (27), because an equivocal result can lead to repeat testing and even a workup for immunologic disease, with concomitant uncertainty and inconvenience (9). Therefore, further research is needed to reduce the indeterminate rate of IGRA.

The results from previous studies that compared the indeterminate rates of QFT and T-SPOT are controversial. In Diel et al.’s study (15), the pooled indeterminate rate for QFT-GIT (2.1%, 95% CI 2.0%–2.3%) was lower than that for T-SPOT (3.8%, 95% CI 3.5%−4.2%). Huo et al. found a significantly lower pooled indeterminate rate for QFT-GIT than for T-SPOT among HIV-positive individuals (10). The authors suggested that the more demanding laboratory work for the T-SPOT likely explained the higher indeterminate rates (15). However, although not reaching statistical significance, Meier et al. reported a higher indeterminate rate for QFT (5.0%, 95% CI 4.0%–6.0%) than for T-SPOT (3.0%, 95% CI 2.0%–5.0%) (16). Santin et al. also reported a higher pooled indeterminate rate for QFT-GIT than for T-SPOT (11). Our meta-analysis included more head-to-head studies and provided more comprehensive results. The comparison of 55 head-to-head studies revealed that the pooled indeterminate rate for QFT was similar to the pooled indeterminate rate for T-SPOT. Nevertheless, subgroup analysis revealed that the indeterminate rate for QFT-G was significantly higher than that for T-SPOT, the indeterminate rate for QFT-GIT was similar to that for T-SPOT, and the indeterminate rate of QFT-plus was significantly lower than that for T-SPOT. Although the finding did not reach statistical significance, further analysis found that the indeterminate rate for QFT-plus was the lowest across all generations of QFT. Therefore, our study revealed a potential advantage to using the newest generation of QFT (QFT-plus), owing to its low indeterminate rate.

Immunosuppression is an important risk factor for indeterminate IGRA results. Park et al. included six studies and reported that the indeterminate rate of IGRA was higher in patients on immunosuppression treatment than in those not on immunosuppression treatment (pooled OR = 2.91, 95% CI 1.36–6.24) (12). Santin et al. included 11 studies and found that the indeterminate rates of IGRA were higher in HIV-infected than in HIV-uninfected individuals; however, the difference did not reach statistical significance (11). Santin et al. also reported that the pooled indeterminate rate of IGRA for those with a CD4+ cell count of ≥200 was significantly lower than for those with a CD4+ cell count of <200 (11). Meier et al. reported that immunocompromised patients contributed to the indeterminate results in their meta-analysis (16). Our meta-analysis included 16 studies and found that the indeterminate rate for the immunocompromised population was significantly higher than that for the healthy control population (pooled OR = 3.51, 95% CI 2.11–5.82). In HIV-positive patients, we found that the indeterminate rate increased as the CD4+ cell count decreased. Therefore, our study adds to the evidence supporting the correlation between immunosuppression and highly indeterminate results.

There is also concern about the routine use of IGRA in young children (< 5 years), owing to a higher indeterminate rate of IGRA than older children (28, 29). In our study, the indeterminate rate for children was significantly higher than that for adults, and further analysis revealed that the indeterminate rate increased as the age of the children decreased. Our results differ from those reported in Meier et al.’s meta-analysis (16), which assessed the indeterminate rate of IGRA in children and found that the pooled indeterminate rate in the group with median or mean ages of 0–7 years (4.0%, 95% CI 3.0%–6.0%) was similar to those in the group with median or mean ages ≥8 years (4.0%, 95% CI 3.0%–5.0%). Therefore, to our knowledge, this is the first meta-analysis that reported that young children were related to a high indeterminate rate.

We identified that most of the indeterminate results (94.6%, 95% CI 89.6%–98.0%) were caused by failed positive controls and a small portion of indeterminate results (4.0%, 95% CI 1.4% to 12.4%) were caused by failed negative controls (see Table 5). Failed positive controls may be due to an impaired cellular immune response associated with a decrease in the number or function of T lymphocytes, as seen in HIV infection and cancer (30). Although the proportion of indeterminate results caused by failed positive controls was highest in the immunocompetent population (99.4%, 95% CI 97.3%–100%), most of the indeterminate results in the immunocompetent population can be attributed to technical errors, as few indeterminate cases were confirmed as being caused by immunosuppression (31, 32). Failed negative controls may be due to the presence of heterophilic antibodies (e.g., human anti-mouse) or spontaneous IFN-γ secretion during an infection or following vaccination (33). Although the 95% CIs overlapped, the proportion of indeterminate results caused by failed negative controls was higher in the immunocompromised population than in the immunocompetent population. This observation may be due to the higher possibility of infection in the immunocompromised population than in the immunocompetent population. In addition, immunometric assays are inherently vulnerable to interference from heterophilic antibodies, which is particularly relevant in dialysis patients and people with autoimmune diseases or an infection (34–36).

This meta-analysis had several limitations. First, 65 studies (16.1%) did not provide a definition of indeterminate IGRA results, which may have led to inconsistent interpretations of the test results. Second, although we carefully reviewed the methods sections of all included studies and attempted to exclude indeterminate cases caused by technical errors, few included studies reported the relevant information. Therefore, it was difficult to determine the extent to which technical errors may have influenced our overall findings. Third, as 358 studies (88.8%) included in the analysis were conducted in areas with low TB burden, the generalizability of the meta-analysis findings to areas with high TB burden may be limited. Finally, while obvious heterogeneity was present in several groups, subgroup analyses to identify the source of heterogeneity were not possible.

## 5 Conclusion

On average, 1 in 26 tests yields indeterminate IGRA results in LTBI screening. The use of advanced versions of the QuantiFERON-TB assay (QFT-plus) may reduce the occurrence of indeterminate results. Our study emphasizes the high risk of indeterminate IGRA in relation to immunosuppression and young age, which should receive more attention in the management of LTBI.

## Data availability statement

The original contributions presented in the study are included in the article/**Supplementary Material**. Further inquiries can be directed to the corresponding authors.

## Author contributions

GZ, YZZ, and CS initiated the project and were responsible for protocol design. GZ and QL performed the literature review, collected the data, assessed the quality of studies, and analyzed the data. SL, HC, SC, XG, JH, YX, HL, YCZ, and JH interpreted the data. GZ wrote the initial draft of the manuscript. All authors were responsible for critical revision of the manuscript and provided important intellectual content. All authors contributed to the article and approved the submitted version.
