# Supplementary material for: Indeterminate results of interferon gamma release assays in the screening of latent tuberculosis infection: a systematic review and meta-analysis
Source: Front Immunol. 2023 May 15;14:1170579. doi: 10.3389/fimmu.2023.1170579 (PMC10225525; doi:10.3389/fimmu.2023.1170579)
Supplement: Supplementary file 1 [file DataSheet_1.pdf]

Supplementary web appendix for:
Indeterminate events of interferon-gamma release assays in the screening of latent tuberculosis infection: A systematic review and meta-analysis

Content

Figure S1. Forest plot of the odds ratio of the indeterminate rate of QFT vs. the indeterminate rate of T-SPOT 2

Figure S2. Forest plot of the odds ratio of the indeterminate rate of immunocompromised population vs. the indeterminate rate of healthy control. 3

Figure S3. Forest plot of the indeterminate rate of IGRA in HIV-positive patients stratified by CD4+ cell count (subgroup1) 3

Figure S4. Forest plot of the indeterminate rate of IGRA in HIV-positive patients stratified by CD4+ cell count (subgroup2) 4

Figure S5. Forest plot of the odds ratio of the indeterminate rate of children vs. the indeterminate rate of adults 5

Figure S6. Forest plot of the indeterminate rate of IGRA in children stratified by age 5

Table S1. Search strategy 6

Table S2. Populations considered high-risk 7

Table S3. QUADAS-2 adapted quality assessment criteria 7

Table S4. Detailed individual study characteristics. 8

Table S5. Summary of study quality (items from the modified QUADAS-2). 33

Table S6. Meta-regression analysis for the indeterminate rate. 33

Table S7. Subgroup analysis of the indeterminate rate. 35

Table S8.Comparison of IGRA indeterminate rates between QFT and T-SPOT in head-to-head studies. 36

Table S9. Comparison of the indeterminate rates among three generation of QFT. 36

Table S10. Comparison of IGRA indeterminate rates among different immune status. 37

Table S11. Comparison of the indeterminate rates in HIV-positive patients stratified by CD4+ cell count. 37

Table S12. Comparison of IGRA indeterminate rates among different age group. 37

Table S13. Comparison of indeterminate rates of IGRA caused by failed positive control and failed negative control among different population. 38

Pre-specified analysis plan 66

Study	QFT Events	Total	T-SPOT Events	Total	OR	95%−CI	Weight
Abubakar et al 2018	163	8695	1625	9610	0.09	[0.08; 0.11]	2.6%
Adetifa et al 2010	2	215	0	215	5.05	[0.24; 105.74]	1.1%
Ahmed et al 2020	33	3625	15	3625	2.21	[1.20; 4.08]	2.5%
Arias−Guillén et al 2014	5	205	12	199	0.39	[0.13; 1.13]	2.2%
Arias−Guillén et al 2018	2	393	2	393	1.00	[0.14; 7.13]	1.6%
Basu Roy et al 2012	20	1093	6	382	1.17	[0.47; 2.93]	2.3%
Bocchino et al 2008	2	69	4	69	0.49	[0.09; 2.74]	1.8%
Bourgarit et al 2015	10	415	23	415	0.42	[0.20; 0.90]	2.4%
Carvalho et al 2013	3	18	2	18	1.60	[0.23; 10.94]	1.7%
Cheallaigh et al 2013	6	256	18	247	0.31	[0.12; 0.78]	2.3%
Chkhartishvili et al 2013	3	240	20	240	0.14	[0.04; 0.48]	2.1%
Chung et al 2009	21	167	8	167	2.86	[1.23; 6.65]	2.4%
de Souza−Galvao et al 2014	0	156	4	156	0.11	[0.01; 2.03]	1.1%
Domínguez et al 2008	4	584	1	584	4.02	[0.45; 36.08]	1.5%
Dorman et al 2014	37	2418	107	2418	0.34	[0.23; 0.49]	2.6%
Fernández−Blázquez et al 2022	30	1464	130	1535	0.23	[0.15; 0.34]	2.6%
Grant et al 2012	6	79	7	79	0.85	[0.27; 2.64]	2.2%
Hesseling et al 2009	3	82	1	82	3.08	[0.31; 30.20]	1.4%
Ho et al 2021	91	20788	119	20778	0.76	[0.58; 1.00]	2.6%
Igari et al 2017	1	138	4	143	0.25	[0.03; 2.30]	1.5%
Igari et al 2019	0	136	3	136	0.14	[0.01; 2.73]	1.1%
Igari et al 2021	1	184	0	184	3.02	[0.12; 74.53]	1.0%
Ishikawa et al 2017	2	92	2	92	1.00	[0.14; 7.25]	1.6%
Iwagaitsu et al 2016	3	68	1	68	3.09	[0.31; 30.50]	1.4%
Jung et al 2015	3	156	6	156	0.49	[0.12; 2.00]	2.0%
Latorre et al 2010	2	75	2	75	1.00	[0.14; 7.29]	1.6%
Latorre et al 2014	2	103	7	103	0.27	[0.06; 1.34]	1.9%
Leidl et al 2010	4	109	4	109	1.00	[0.24; 4.10]	2.0%
Lucas et al 2010	70	460	8	420	9.24	[4.39; 19.46]	2.4%
Mandalakas et al 2008	3	32	2	43	2.12	[0.33; 13.50]	1.7%
Mandalakas et al 2015	20	1044	1	297	5.78	[0.77; 43.26]	1.6%
Mariette et al 2012	11	392	25	392	0.42	[0.21; 0.87]	2.4%
Matsumura et al 2016	12	230	5	230	2.48	[0.86; 7.15]	2.2%
Mínguez et al 2012	4	53	2	53	2.08	[0.36; 11.88]	1.8%
Nozawa et al 2016	8	81	0	27	6.36	[0.36; 113.95]	1.1%
Pareek et al 2013	2	229	7	229	0.28	[0.06; 1.36]	1.9%
Parrella et al 2015	4	98	13	98	0.28	[0.09; 0.89]	2.2%
Pullar et al 2014	3	298	0	117	2.78	[0.14; 54.30]	1.1%
Ramos et al 2012	10	373	26	373	0.37	[0.17; 0.77]	2.4%
Rangeaka et al 2007	7	100	1	100	7.45	[0.90; 61.73]	1.5%
Rangeaka et al 2007	2	99	0	100	5.15	[0.24; 108.73]	1.1%
Richeldi et al 2009	24	331	2	331	12.86	[3.01; 54.87]	2.0%
Sester et al 2014	109	1532	125	1503	0.84	[0.65; 1.10]	2.6%
Stefan et al 2010	5	34	4	34	1.29	[0.32; 5.30]	2.0%
Stephan et al 2008	1	275	8	275	0.12	[0.02; 0.98]	1.6%
Sultan et al 2013	1	117	0	117	3.03	[0.12; 75.04]	1.0%
Talati et al 2009	6	336	47	336	0.11	[0.05; 0.27]	2.4%
Tanabe et al 2017	2	652	2	652	1.00	[0.14; 7.12]	1.6%
Tavast et al 2012	1	53	2	82	0.77	[0.07; 8.70]	1.4%
Triverio et al 2009	5	62	7	62	0.69	[0.21; 2.30]	2.1%
Tsuyuzaki et al 2019	0	412	0	412	1.00	[0.02; 50.52]	0.8%
Vassilopoulos et al 2011	2	157	0	157	5.06	[0.24; 106.34]	1.1%
Winthrop et al 2008	2	124	3	124	0.66	[0.11; 4.03]	1.7%
Zhang et al 2019	0	625	0	625	1.00	[0.02; 50.48]	0.8%
Zhang et al 2022	13	108	6	108	2.33	[0.85; 6.37]	2.3%
**Random effects model**		**50330**		**49575**	**0.88**	**[0.59; 1.32]**	**100.0%**

Heterogeneity: $I^2 = 91\%$, $\tau^2 = 1.6076$, $p < 0.01$

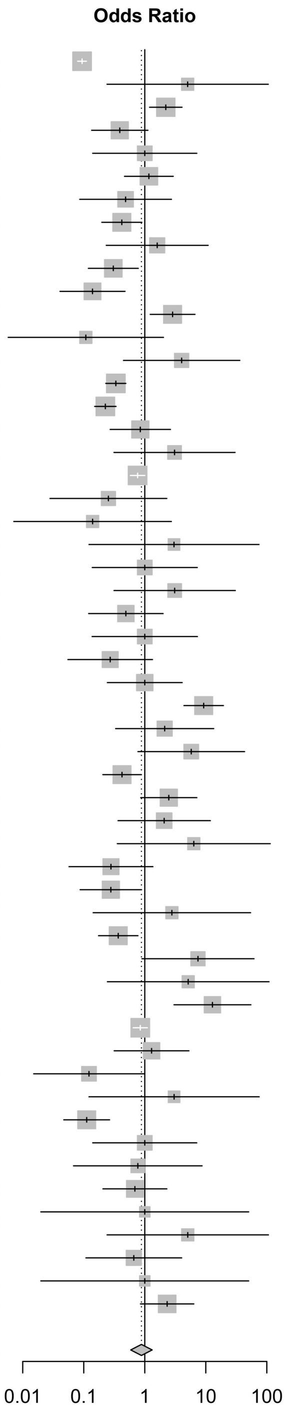

Figure S1. Forest plot of the odds ratio of the indeterminate rate of QFT vs. the indeterminate rate of T-SPOT

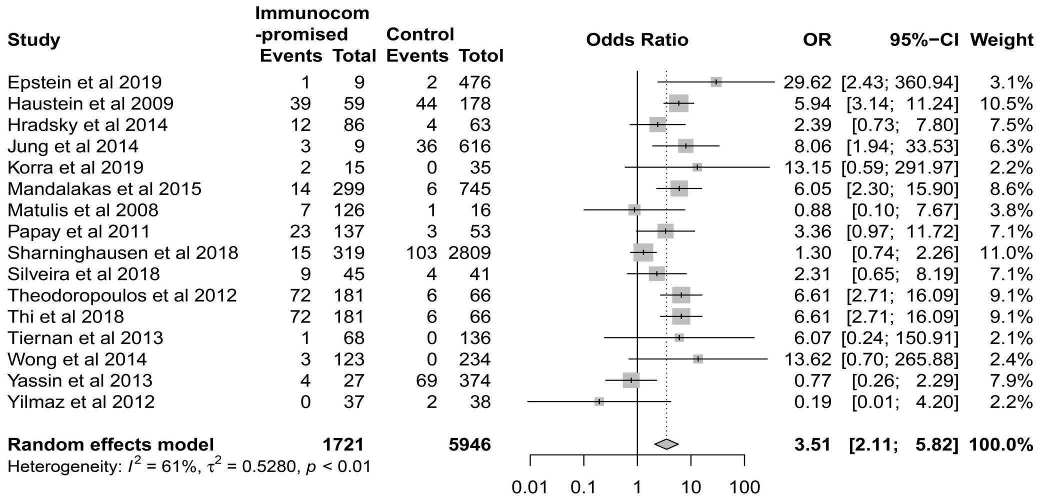

Figure S2. Forest plot of the odds ratio of the indeterminate rate of immunocompromised population vs. the indeterminate rate of healthy control.

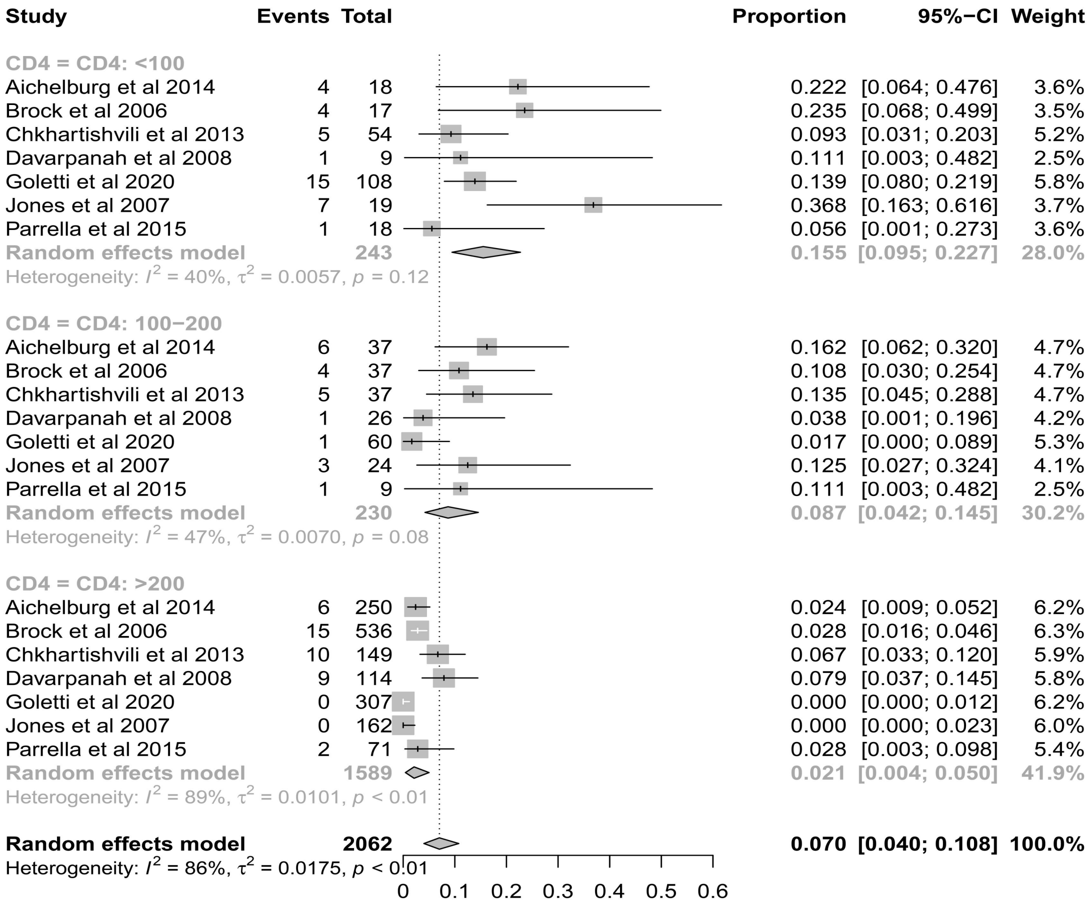

Figure S3. Forest plot of the indeterminate rate of IGRA in HIV-positive patients stratified by CD4+ cell count (subgroup1)

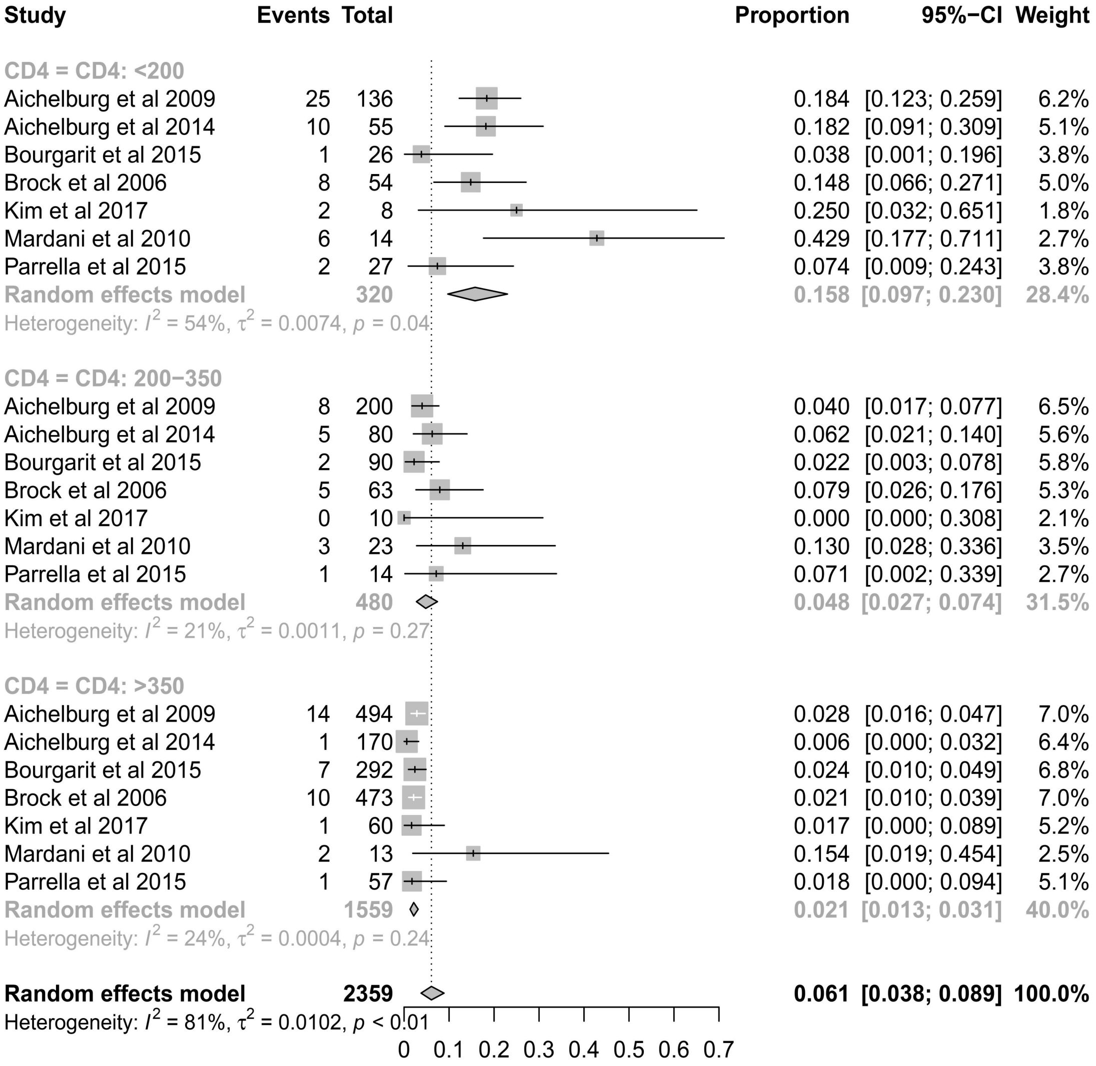

Figure S4. Forest plot of the indeterminate rate of IGRA in HIV-positive patients stratified by CD4+ cell count (subgroup2)

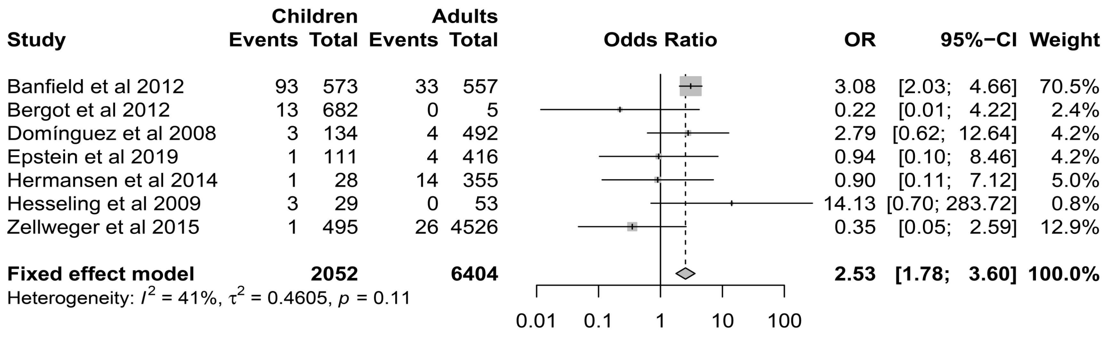

Figure S5. Forest plot of the odds ratio of the indeterminate rate of children vs. the indeterminate rate of adults

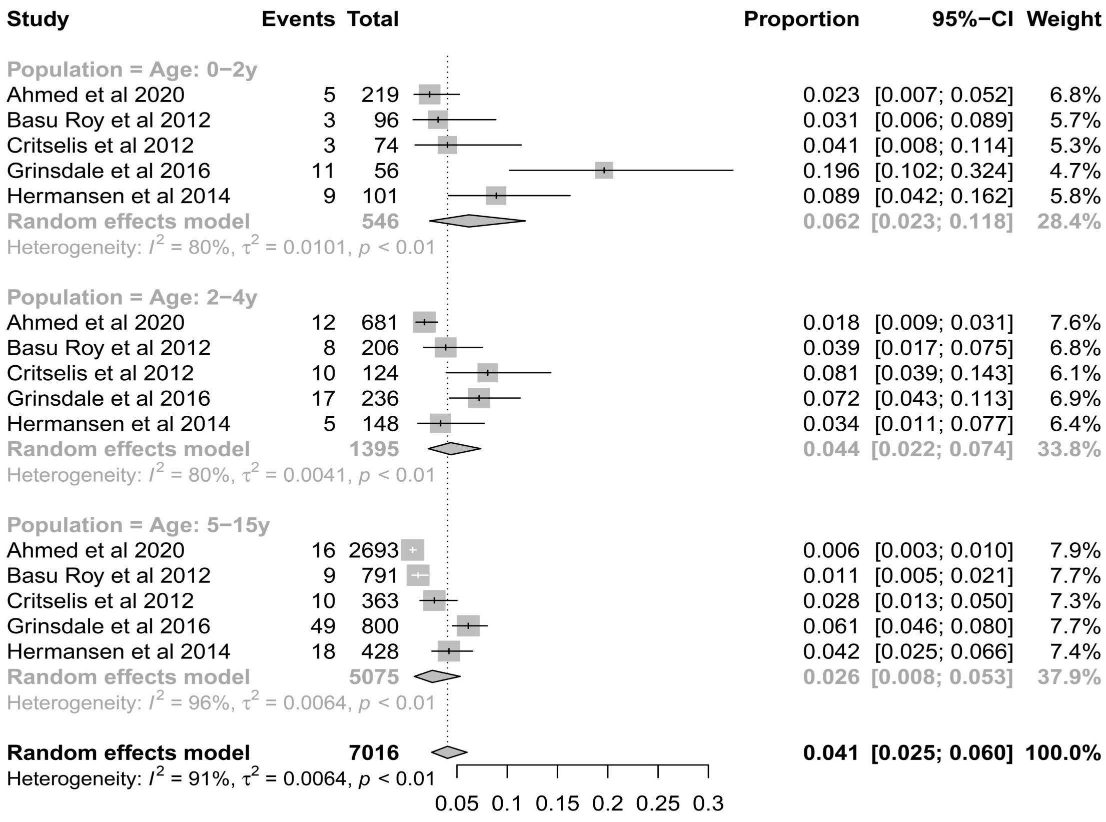

Figure S6. Forest plot of the indeterminate rate of IGRA in children stratified by age

Table S1. Search strategy

Search strategy in PubMed		
#1	(((("Latent Tuberculosis"[Mesh]) OR (Latent Tuberculosis Infections)) OR (Tuberculosis Infection)) OR (Latent Tuberculosis)) OR (LTBI)	283,941
#2	(((((((((("Interferon-gamma Release Tests"[Mesh]) OR (Interferon-gamma Release Tests)) OR (Interferon-gamma Release Assay)) OR (Interferon gamma Release Assays)) OR (Interferon gamma Release Tests)) OR (Interferon-gamma Release Test)) OR (IGRA)) OR (IGRA)) OR (quantiferontb)) OR (QFT)) OR (T-SPOT-TB)) OR (T-SPOT)	6914
#3	Indeterminate	16,801
#4	#1 AND #2 AND #3	419
Search strategy in Embase		
#1	'latent tuberculosis'/exp OR (latent tuberculosis infections) OR (Tuberculosis Infection) OR (latent tuberculosis) OR ltbi	108,456
#2	'interferon gamma release assay'/exp OR (interferon AND gamma release assay) OR interferon gamma release assays OR interferon gamma release tests OR (interferon AND gamma release test) OR igra OR qft OR 't spot'	18,596
	Indeterminate	25,123
#4	#1 AND #2 AND #3	614
Search strategy in Cochrane Library		
#1	(Latent Tuberculosis)[Mesh] OR (Latent Tuberculosis Infections OR Tuberculosis, Latent OR Latent Tuberculoses OR Infections, Latent Tuberculosis OR Latent Tuberculosis Infection OR Tuberculoses, Latent OR Tuberculosis Infection, Latent OR Infection, Latent Tuberculosis OR Tuberculosis Infections, Latent OR LTBI):ti,ab,kw	525
#2	(Interferon-gamma Release Tests)[Mesh] OR (Interferon gamma Release Assays OR Release Assay, Interferon-gamma OR Assays, Interferon-gamma Release OR Release Assays, Interferon-gamma OR Release Test, Interferon-gamma OR Interferon gamma Release Assay OR Interferon-gamma Release Assay OR Test, Interferon-gamma Release OR Tests, Interferon-gamma Release OR Interferon-gamma Release Test OR Assay, Interferon-gamma Release OR Interferon gamma Release Tests OR Interferon-gamma Release Assays OR Release Tests, Interferon-gamma):ti,ab,kw	239
#3	Indeterminate	745
#4	#1 AND #2 AND #3	95

Table S2. Populations considered high-risk

Immunocompetent population	Healthy people, recent contacts and population with the possibility of contact
Recent contacts	Both close/household and casual
With the possibility of contact	1.Occupational risk (e.g. healthcare worker); 2.Immigrants or refugees.
Immunocompromised population	1.People living with HIV; 2.Chronic renal failure and/or haemodialysis; 3.Transplant recipients (organ or hematopoietic stem cell); 4.Drug and/or alcohol abusers; 5.Cancer (all types); 6.Malnourished (BMI ≤ 18.5 kg/m); 7.Silicosis.
With the possibility of immunosuppression	1.IMID; 2. Prisoners; 3.Children; 4.Nursing home residents; 5.Homeless.

Table S3. QUADAS-2 adapted quality assessment criteria

Participants select bias	Q1: Was a consecutive or random sample of participants enrolled?<br>We scored "Yes" if a consecutive or random sample of eligible patients was Enrolled; "No"if patients were selected by convenience; and "Unclear" if the study did not report the manner in which patients were enrolled.<br>Q2: Did the study have appropriate exclusions?<br>We scored "Yes" if current active TB and people with TB symptom were excluded, or they were grouped separately, or the definition of LTBI was stated as asymptomatic; "No" if active TB and people with TB symptom were not excluded; and "Unclear" if this was not stated.
Test conduct bias	Q3: Were the tests adequately described?<br>We scored "Yes" if the tests were conducted and interpreted adequately described, such as the cut-off, time interval of the results were read, and manufacturer information, or "performed as the manufacturer's guidelines" was stated; "Unclear" if those information were not stated, "No" if this was stated inadequately.<br>Q4: Was the definition of indeterminate for IGRA adequately described?<br>We scored "Yes" if the definition of indeterminate for IGRA adequately described or "the definition of indeterminate was refer to the manufacturer's guidelines" was stated; "No" if those information were not stated inadequately; "Unclear" if this was not stated.
Flow and outcome	Q5: Were all participants included in the analysis?<br>We scored "Yes" if more than 80% of participants were included in the analysis; "No" if less than 80% of participants were included in the analysis; and "Unclear" if this was not stated.<br>Q6: Was the reason for the participant's withdrawal from the analysis explained?<br>We scored "Yes" if the reason for participant's withdrawal from the analysis was explained or if no participant dropped from the analysis, "No" if the reason was not stated.

Table S4. Detailed individual study characteristics.

	Area	TB burden	Study design	Prospective or retrospective	Subgroup	Population	Age group	Male (%)	Type of IGRA	Particip -ants (n)	Indeterm -inate (n)	Quadas score
Abdelwahab et al 2021[1]	Egypt	0–30	NS	NS	All	Cancer	Adults	59.4	QFT-GIT	64	8	4
Abdulkareem et al 2020[2]	Iraq	31–100	Cross-sectional	Prospective	All	Contacts	All age	47.8	QFT-plus	403	2	5
Abreu et al 2016[3]	Portugal	0–30	Cohort	NS	All	IMID	Adults	44	QFT-GIT	250	6	6
Abubakar et al 2018[4]	UK	0–30	Cohort	Prospective	All	Contact and migrants	All age	50	QFT-GIT	8695	163	5
Abubakar et al 2018[4]	UK	0–30	Cohort	Prospective	All	Contact and migrants	All age	50	T-SPOT	9610	1625	5
Adetifa et al 2010[5]	Gambia	101–200	NS	NS	All	Contacts	Children	49.1	QFT-GIT	215	2	5
Adetifa et al 2010[5]	Gambia	101–200	NS	NS	All	Contacts	Children	49.1	T-SPOT	215	0	5
Agarwal et al 2016[6]	USA	0–30	Cross-sectional	NS	All	HCWs	Adults	24.83	T-SPOT	2481	42	6
Ahmadinejad et al 2012[7]	Iran	0–30	Cross-sectional	NS	All	Transplant candidates	Adults	59.4	QFT-GIT	64	4	6
Ahmadinejad et al 2013[8]	Iran	0–30	NS	NS	All	Transplant candidates	All age	53.7	QFT-GIT	164	5	5
Ahmed et al 2020[9]	USA	0–30	Cohort	Prospective	All	Contacts	Children	50.5	QFT-GIT	3625	33	6
Ahmed et al 2020[9]	USA	0–30	Cohort	Prospective	All	Contacts	Children	50.5	T-SPOT	3625	15	6
Ahmed et al 2020[9]	USA	0–30	Cohort	Prospective	Age: 0-2y	Contacts	Children	50.5	QFT-GIT	219	5	6
Ahmed et al 2020[9]	USA	0–30	Cohort	Prospective	Age: 2-4y	Contacts	Children	50.5	QFT-GIT	681	12	6
Ahmed et al 2020[9]	USA	0–30	Cohort	Prospective	Age: 5-15y	Contacts	Children	50.5	QFT-GIT	2693	16	6
Aichelburg et al 2009[10]	Austria	0–30	Cohort	Prospective	All	HIV-positive	Adults	70	QFT-GIT	822	47	6
Aichelburg et al 2009[10]	Austria	0–30	Cohort	Prospective	CD4: <200	HIV-positive	Adults	70	QFT-GIT	136	25	6
Aichelburg et al 2009[10]	Austria	0–30	Cohort	Prospective	CD4: 200-350	HIV-positive	Adults	70	QFT-GIT	200	8	6
Aichelburg et al 2009[10]	Austria	0–30	Cohort	Prospective	CD4: ≥350	HIV-positive	Adults	70	QFT-GIT	494	14	6
Aichelburg et al 2014[11]	Austria	0–30	Cohort	Prospective	All	HIV-positive	Adults	71	QFT-GIT	846	52	4
Aichelburg et al 2014[12]	Austria	0–30	Cross-sectional	NS	All	HIV-positive	Adults	73	QFT-GIT	305	16	6
Aichelburg et al 2014[12]	Austria	0–30	Cross-sectional	NS	CD4: <100	HIV-positive	Adults	73	QFT-GIT	18	4	6
Aichelburg et al 2014[12]	Austria	0–30	Cross-sectional	NS	CD4: 100-200	HIV-positive	Adults	73	QFT-GIT	37	6	6
Aichelburg et al 2014[12]	Austria	0–30	Cross-sectional	NS	CD4: 201-200	HIV-positive	Adults	73	QFT-GIT	80	5	6
Aichelburg et al 2014[12]	Austria	0–30	Cross-sectional	NS	CD4: ≥350	HIV-positive	Adults	73	QFT-GIT	170	1	6
Al Hajoj et al 2016[13]	Saudi Arabia	0–30	Cross-sectional	NS	All	HCWs	Adults	32.1	QFT-GIT	1603	8	6

Continue

	Area	TB burden	Study design	Prospective or retrospective	Subgroup	Population	Age group	Male (%)	Type of IGRA	Particip-ants (n)	Indeterm-inate (n)	Quadas score
Al Mekaini et al 2014[14]	United Arab Emirates	0–30	Cross-sectional	Prospective	All	Healthy people	Children	46	QFT-G	666	2	5
Al-Taweel et al 2018[15]	Canada	0–30	Cross-sectional	Prospective	All	IMID	NS	51.7	QFT-GIT	155	6	6
Amorim et al 2019[16]	Brazil	31–100	Cross-sectional	NS	All	IMID	NS	36.13	QFT-GIT	103	4	6
Andrews et al 2015[17]	South Africa	201–	Cohort	Prospective	All	Children	Children	NS	QFT-GIT	2751	13	6
Andrews et al 2017[18]	South Africa	201–	RCT	NS	All	Children	Young children	NS	QFT-GIT	2797	20	6
Andrisani et al 2013[19]	Italy	0–30	Cohort	Prospective	All	IMID	Adults	50	QFT-GIT	92	1	6
Arenas Miras Mdel et al 2014[20]	Spain	0–30	Cross-sectional	NS	All	IMID	All age	92	T-SPOT	92	4	6
Arias-Guillén et al 2014[21]	Spain	0–30	Cohort	Prospective	All	IMID	NS	49.75	QFT-GIT	205	5	5
Arias-Guillén et al 2014[21]	Spain	0–30	Cohort	Prospective	All	IMID	NS	49.75	T-SPOT	199	12	5
Arias-Guillén et al 2018[22]	Spain	0–30	Cohort	Prospective	All	IMID	Adults	37.4	QFT-GIT	393	2	4
Arias-Guillén et al 2018[22]	Spain	0–30	Cohort	Prospective	All	IMID	Adults	37.4	T-SPOT	393	2	4
Atikan et al 2016[23]	Turkey	0–30	Cohort	Retrospective	All	IMID	Children	47.88	QFT-GIT	71	1	4
Balcells et al 2008[24]	Chile	0–30	Cross-sectional	Prospective	All	HIV-positive	Adults	85.7	QFT-GIT	115	0	6
Baldassari et al 2019[25]	USA	0–30	NS	Retrospective	All	IMID	Adults	31.9	QFT-G	1058	65	4
Balkhy et al 2017[26]	Saudi Arabia	0–30	Cross-sectional	NS	All	Healthy people	All age	43.6	QFT-GIT	1443	27	5
Banach et al 2011[27]	USA	0–30	Cross-sectional	Retrospective	All	High risk	All age	46.49	QFT-G	28864	522	5
Bandiara et al 2022[28]	Indonesia	101–200	Cross-sectional	NS	All	Dialysis	Adults	47	IGRA	120	5	5
Banfield et al 2012[29]	Australia	0–30	Cross-sectional	Retrospective	All	Refugees and immigrants	All age	49	IGRA	1130	126	5
Banfield et al 2012[29]	Australia	0–30	Cross-sectional	Retrospective	Adults	Refugees and immigrants	Adults	49	IGRA	557	33	5
Banfield et al 2012[29]	Australia	0–30	Cross-sectional	Retrospective	Children	Children	Children	49	IGRA	573	93	5
Bartalesi et al 2009[30]	Italy	0–30	Cohort	Prospective	All	IMID	All age	65.1	QFT-GIT	398	5	6
Bartalesi et al 2013[31]	Italy	0–30	Cohort	NS	All	IMID	All age	34	QFT-GIT	1485	10	6
Basu Roy et al 2012[32]	European countries	0–30	Cross-sectional	Retrospective	All	Children	Children	50.4	QFT-GIT	1093	20	6
Basu Roy et al 2012[32]	European countries	0–30	Cross-sectional	Retrospective	All	Children	Children	50.4	T-SPOT	382	6	6

Continue

	Area	TB burden	Study design	Prospective or retrospective	Subgroup	Population	Age group	Male (%)	Type of IGRA	Particip -ants (n)	Indeterm -inate (n)	Quadas score
Basu Roy et al 2012[32]	European countries	0–30	Cross-sectional	Retrospective	Age: 0-2y	Children	Children	50.4	QFT-GIT	96	3	6
Basu Roy et al 2012[32]	European countries	0–30	Cross-sectional	Retrospective	Age: 2-5y	Children	Children	50.4	QFT-GIT	206	8	6
Basu Roy et al 2012[32]	European countries	0–30	Cross-sectional	Retrospective	Age: 5-15y	Children	Children	50.4	QFT-GIT	791	9	6
Behar et al 2009[33]	USA	0–30	Cross-sectional	Prospective	All	IMID	Adults	25.7	T-SPOT	179	0	6
Bélard et al 2011[34]	Denmark	0–30	Cohort	Prospective	All	IMID	Adults	33	QFT-GIT	248	12	6
Bergot et al 2012[35]	France	0–30	Cohort	Prospective	All	Contacts	All age	30.3	QFT-GIT	687	13	6
Bergot et al 2012[35]	France	0–30	Cohort	Prospective	Adults	Contacts	All age	30.3	QFT-GIT	5	0	6
Bergot et al 2012[35]	France	0–30	Cohort	Prospective	Children	Contacts	All age	30.3	QFT-GIT	682	13	6
Beshir et al 2016[36]	Egypt	0–30	Cross-sectional	NS	All	Healthy people	Young children	52	QFT-GIT	150	3	6
Bianchi et al 2009[37]	Italy	0–30	Cohort	Prospective	All	Children	Children	59.2	QFT-GIT	320	2	6
Birku et al 2020[38]	Ethiopia	101–200	Cross-sectional	Retrospective	All	HIV-positive	Adults	100	QFT-GIT	193	2	6
Birku et al 2020[38]	Ethiopia	101–200	Cross-sectional	Retrospective	All	HIV-negaitive	Adults	100	QFT-GIT	127	1	6
Bocchino et al 2008[39]	Italy	0–30	NS	Prospective	All	IMID	Adults	56.5	QFT-GIT	69	2	6
Bocchino et al 2008[39]	Italy	0–30	NS	Prospective	All	IMID	Adults	56.5	T-SPOT	69	4	6
Borgia et al 2011[40]	Italy	0–30	NS	NS	All	Contacts	Children	NS	QFT-GIT	1340	3	4
Bouley et al 2021[41]	USA	0–30	NS	Retrospective	All	IMID	Adults	35.1	QFT-GIT	222	28	5
Bourgarit et al 2015[42]	France	0–30	Cohort	Prospective	All	HIV-positive	Adults	74.2	QFT-GIT	415	10	6
Bourgarit et al 2015[42]	France	0–30	Cohort	Prospective	All	HIV-positive	Adults	74.2	T-SPOT	415	23	6
Bourgarit et al 2015[42]	France	0–30	Cohort	Prospective	CD4: <150	HIV-positive	Adults	74.2	QFT-GIT	26	1	6
Bourgarit et al 2015[42]	France	0–30	Cohort	Prospective	CD4: 150-350	HIV-positive	Adults	74.2	QFT-GIT	90	2	6
Bourgarit et al 2015[42]	France	0–30	Cohort	Prospective	CD4: 350-500	HIV-positive	Adults	74.2	QFT-GIT	109	4	6
Bourgarit et al 2015[42]	France	0–30	Cohort	Prospective	CD4: ≥500	HIV-positive	Adults	74.2	QFT-GIT	183	3	6
Bradshaw et al 2011[43]	Northern Ireland	0–30	NS	NS	All	Immunocompromised patients	Adults	54.7	QFT-GIT	423	41	6
Bradshaw et al 2011[43]	Northern Ireland	0–30	NS	NS	All	HCWs	Adults	54.7	QFT-GIT	98	1	6
Brock et al 2006[44]	Denmark	0–30	Cohort	NS	All	HIV-positive	Adults	74	QFT-GIT	590	20	6

Continue

	Area	TB burden	Study design	Prospective or retrospective	Subgroup	Population	Age group	Male (%)	Type of IGRA	Particip -ants (n)	Indeterm -inate (n)	Quadas score
Brock et al 2006[44]	Denmark	0–30	Cohort	NS	CD4: <100	HIV-positive	Adults	74	QFT-GIT	17	4	6
Brock et al 2006[44]	Denmark	0–30	Cohort	NS	CD4: 100-199	HIV-positive	Adults	74	QFT-GIT	37	4	6
Brock et al 2006[44]	Denmark	0–30	Cohort	NS	CD4: 200-299	HIV-positive	Adults	74	QFT-GIT	63	5	6
Brock et al 2006[44]	Denmark	0–30	Cohort	NS	CD4: >300	HIV-positive	Adults	74	QFT-GIT	473	10	6
Brown et al 2017[45]	UK	0–30	NS	Retrospective	All	HCWs	Adults	41.6	QFT-G	3002	208	3
Bua et al 2011[46]	Italy	0–30	NS	NS	All	HIV-positive	Adults	69	QFT-GIT	73	12	3
Buonsenso et al 2020[47]	Italy	0–30	Cross-sectional	Prospective	All	Children	Children	56.1	QFT-plus	155	5	4
Cabriada et al 2018[48]	Spain	0–30	Cohort	Prospective	All	IMID	NS	44	QFT-GIT	138	4	4
Calabrese et al 2015[49]	USA	0–30	NS	Retrospective	All	IMID	Adults	31.5	QFT-GIT	2064	152	6
Calabrese et al 2015[49]	USA	0–30	NS	Retrospective	All	Healthy people	Adults	31.5	QFT-GIT	25670	382	6
Calzada-Hernández et al 2015[50]	Spain	0–30	Cohort	Retrospective	All	IMID	Children	NS	QFT-GIT	75	3	5
Calzada-Hernández et al 2022[51]	Spain	0–30	Cohort	NS	All	IMID	Children	NS	QFT-GIT	270	10	6
Carvalho et al 2013[52]	Italy	0–30	NS	NS	All	Children	Children	61	QFT-GIT	18	3	5
Carvalho et al 2013[52]	Italy	0–30	NS	NS	All	Children	Children	61	T-SPOT	18	2	5
Casas et al 2011[53]	Spain	0–30	Cross-sectional	Prospective	All	Transplant candidates	Adults	75.8	QFT-GIT	95	2	6
Çekiç et al 2015[54]	Turkey	0–30	Cohort	Retrospective	All	IMID	Adults	69.7	IGRA	76	3	3
Chang et al 2011[55]	South Korea	31–100	Cohort	NS	All	IMID	Adults	59	QFT-GIT	107	7	6
Cheallaigh et al 2013[56]	Ireland	0–30	NS	NS	All	HIV-positive	Adults	59	QFT-GIT	256	6	6
Cheallaigh et al 2013[56]	Ireland	0–30	NS	NS	All	HIV-positive	Adults	59	T-SPOT	247	18	6
Cheallaigh et al 2013[56]	Ireland	0–30	NS	NS	CD4: <100	HIV-positive	Adults	59	QFT-GIT	37	2	6
Cheallaigh et al 2013[56]	Ireland	0–30	NS	NS	CD4: >100	HIV-positive	Adults	59	QFT-GIT	219	4	6
Chen et al 2008[57]	USA	0–30	Cohort	Prospective	All	IMID	Adults	77.1	QFT-G	43	2	5
Chen et al 2012[58]	Taiwan, China	31–100	Cohort	Prospective	All	IMID	NS	82	QFT-G	242	9	5
Cheng et al 2015[59]	China, Taiwan	31–100	Cohort	Retrospective	All	IMID	Adults	68.3	QFT-GIT	101	6	5
Chiappini et al 2012[60]	Italy	0–30	Cohort	Prospective	All	Children	Children	NS	QFT-GIT	50	1	6
Chien et al 2018[61]	China, Taiwan	31–100	NS	NS	All	Older people	Adults	51	QFT-plus	229	1	6
Chiu et al 2011[62]	China, Taiwan	31–100	Cohort	Retrospective	All	IMID	All age	73	QFT-G	110	3	5
Chkhartishvili et al 2013[63]	USA	0–30	Cross-sectional	NS	All	HIV-positive	Adults	66	QFT-GIT	240	3	6

Continue

	Area	TB burden	Study design	Prospective or retrospective	Subgroup	Population	Age group	Male (%)	Type of IGRA	Particip -ants (n)	Indeterm -inate (n)	Quadas score
Chkhartishvili et al 2013[63]	USA	0–30	Cross-sectional	NS	All	HIV-positive	Adults	66	QFT-GIT	240	20	6
Chkhartishvili et al 2013[63]	USA	0–30	Cross-sectional	NS	CD4: <100	HIV-positive	Adults	66	QFT-GIT	54	5	6
Chkhartishvili et al 2013[63]	USA	0–30	Cross-sectional	NS	CD4: 100-200	HIV-positive	Adults	66	QFT-GIT	37	5	6
Chkhartishvili et al 2013[63]	USA	0–30	Cross-sectional	NS	CD4: ≥200	HIV-positive	Adults	66	T-SPOT	149	10	6
Cho et al 2016[64]	South Korea	31–100	NS	Prospective	All	IMID	Adults	14.7	QFT-G	202	33	6
Chuke et al 2014[65]	USA	0–30	Cross-sectional	NS	All	Refugees and immigrants	Adults	67.6	QFT-GIT	974	65	5
Chun et al 2008[66]	South Korea	31–100	Cohort	NS	All	Contacts	Children	51.7	QFT-GIT	71	2	5
Chun et al 2008[66]	South Korea	31–100	Cohort	NS	All	Healthy people	Children	51.7	QFT-GIT	65	0	5
Chung et al 2009[67]	South Korea	31–100	NS	NS	All	Dialysis	Adults	57.5	QFT-G	167	21	6
Chung et al 2009[67]	South Korea	31–100	NS	NS	All	Dialysis	Adults	57.5	T-SPOT	167	8	6
Chung et al 2010[68]	South Korea	31–100	Cohort	NS	All	Dialysis	Adults	60.2	QFT-GIT	162	21	5
Cobanoglu et al 2007[69]	Turkey	0–30	NS	Prospective	All	IMID	All age	NS	QFT-GIT	59	7	5
Cobanoglu et al 2007[69]	Turkey	0–30	NS	Prospective	All	Healthy people	All age	NS	QFT-GIT	36	2	5
Compagno et al 2022[70]	Italy	0–30	Cohort	Retrospective	All	Transplant candidates	Adults	55.4	QFT-GIT	256	11	5
Costantino et al 2013[71]	France	0–30	Cross-sectional	Prospective	All	IMID	Adults	50.31	T-SPOT	563	88	6
Critselis et al 2012[72]	Greece	0–30	Cross-sectional	NS	All	Children	Children	49.9	QFT-GIT	761	23	6
Critselis et al 2012[72]	Greece	0–30	Cross-sectional	NS	Age: 0-2y	Children	Children	49.9	QFT-GIT	74	3	6
Critselis et al 2012[72]	Greece	0–30	Cross-sectional	NS	Age: 2-4y	Children	Children	49.9	QFT-GIT	124	10	6
Critselis et al 2012[72]	Greece	0–30	Cross-sectional	NS	Age: 5-9y	Children	Children	49.9	QFT-GIT	92	8	6
Critselis et al 2012[72]	Greece	0–30	Cross-sectional	NS	Age: 10-15y	Children	Children	49.9	QFT-GIT	271	2	6
Cruz et al 2015[73]	USA	0–30	NS	Prospective	All	HIV-positive	Children	42	QFT-G	100	3	4
Cummings et al 2009[74]	USA	0–30	NS	Prospective	All	HCWs	Adults	NS	QFT-GIT	267	16	4
Davarpanah et al 2008[75]	Iran	0–30	NS	NS	All	HIV-positive	Adults	95.6	QFT-GIT	152	13	5
Davarpanah et al 2008[75]	Iran	0–30	NS	NS	CD4: <100	HIV-positive	Adults	95.6	QFT-GIT	9	1	5
Davarpanah et al 2008[75]	Iran	0–30	NS	NS	CD4: 100-200	HIV-positive	Adults	95.6	QFT-GIT	26	1	5
Davarpanah et al 2008[75]	Iran	0–30	NS	NS	CD4: ≥200	HIV-positive	Adults	95.6	QFT-GIT	114	9	5
de Oliveira Rodrigues et al 2021[76]	Brazil	31–100	Cohort	Prospective	All	Transplant candidates	All age	62.4	QFT-GIT	126	3	6

Continue

	Area	TB burden	Study design	Prospective or retrospective	Subgroup	Population	Age group	Male (%)	Type of IGRA	Particip-ants (n)	Indeterm-inate (n)	Quadas score
de Souza-Galvão et al 2014[77]	Spain	0–30	NS	Prospective	All	Contacts	All age	43.6	QFT-GIT	156	0	5
de Souza-Galvão et al 2014[77]	Spain	0–30	NS	Prospective	All	Contacts	All age	43.6	T-SPOT	156	4	5
Delgado et al 2011[78]	Spain	0–30	Cross-sectional	NS	All	Contacts	All age	NS	QFT-GIT	376	3	6
Di Renzi et al 2012[79]	Italy	0–30	Cross-sectional	NS	All	HCWs	Adults	NS	QFT-GIT	64	3	6
Diel et al 2011[80]	Germany	0–30	Cohort	NS	All	Contacts	All age	51.3	QFT-GIT	1417	3	6
Dirix et al 2016[81]	Uganda	101–200	Cohort	Prospective	All	HIV-positive	NS	NS	QFT-GIT	156	11	5
DO et al 2022[82]	South Korea	31–100	Cohort	NS	All	IMID	Adults	41.6	QFT-GIT	113	11	5
do Valle Leone de Oliveira et al 2018[83]	Brazil	31–100	Cross-sectional	NS	All	Drug and alcohol abusers	Adults	8.3	QFT-GIT	372	9	6
Domínguez et al 2008[84]	German	0–30	Cohort	Prospective	All	Contacts	All age	41.3	QFT-GIT	584	4	5
Domínguez et al 2008[84]	German	0–30	Cohort	Prospective	All	Contacts	All age	41.3	T-SPOT	584	1	5
Domínguez et al 2008[84]	German	0–30	Cohort	Prospective	Adults	Contacts	All age	41.3	T-SPOT	492	4	5
Domínguez et al 2008[84]	German	0–30	Cohort	Prospective	Children	Contacts	All age	41.3	T-SPOT	134	3	5
Dorman et al 2014[85]	USA	0–30	Cross-sectional	NS	All	HCWs	Adults	24.8	QFT-GIT	2418	37	6
Dorman et al 2014[85]	USA	0–30	Cross-sectional	NS	All	HCWs	Adults	24.8	T-SPOT	2418	107	6
Doyle et al 2014[86]	Australia	0–30	Cohort	Retrospective	All	HIV-positive	All age	88	QFT-G	917	12	6
Drabe et al 2016[87]	Tanzania	201–	NS	NS	All	HIV-positive	All age	14	QFT-GIT	57	1	5
Edathodu et al 2017[88]	Saudi Arabia	0–30	Cohort	Prospective	All	Transplant candidates	All age	59.7	QFT-GIT	105	8	5
Epstein et al 2019[89]	USA	0–30	Cohort	Prospective	All	Refugees and immigrants	All age	58.2	QFT-GIT	527	5	4
Epstein et al 2019[89]	USA	0–30	Cohort	Prospective	HIV-positive	Refugees and immigrants	All age	58.2	QFT-GIT	9	1	4
Epstein et al 2019[89]	USA	0–30	Cohort	Prospective	HIV-negative	Refugees and immigrants	All age	58.2	QFT-GIT	476	2	4
Epstein et al 2019[89]	USA	0–30	Cohort	Prospective	Adults	Refugees and immigrants	All age	58.2	QFT-GIT	416	4	4
Epstein et al 2019[89]	USA	0–30	Cohort	Prospective	Children	Refugees and immigrants	All age	58.2	QFT-GIT	111	1	4
Erol et al 2018[90]	Turkey	0–30	Cross-sectional	Retrospective	All	Transplant candidates	Adults	47.6	QFT-GIT	111	3	5
Escalante et al 2015[91]	USA	0–30	Cohort	Prospective	All	Refugees and immigrants	Adults	21.8	T-SPOT	99	6	6

Continue

	Area	TB burden	Study design	Prospective or retrospective	Subgroup	Population	Age group	Male (%)	Type of IGRA	Particip-ants (n)	Indeterm-inate (n)	Quadas score
Fan et al 2014[92]	China, Taiwan	31–100	Cross-sectional	Prospective	All	Cancer	Adults	59.2	QFT-GIT	340	30	6
Fernández-Blázquez et al 2022[93]	Spain	0–30	NS	Prospective	All	Immunocompromised patients	All age	51	QFT-GIT	1535	50	6
Fernández-Blázquez et al 2022[93]	Spain	0–30	NS	Prospective	All	Immunocompromised patients	All age	51	QFT-plus	1464	30	6
Fernández-Blázquez et al 2022[93]	Spain	0–30	NS	Prospective	All	Immunocompromised patients	All age	51	T-SPOT	1535	130	6
Ferrarini et al 2016[94]	Brazil	31–100	Cohort	Prospective	All	Contacts	Children	52.5	QFT-GIT	59	2	6
Fong et al 2022[95]	USA	0–30	NS	Retrospective	All	HCWs	Adults	47.7	QFT-GIT	7374	305	5
Fox et al 2009[96]	Israel	0–30	Cross-sectional	Prospective	All	HCWs	Adults	31	QFT-GIT	100	9	6
Freeman et al 2012[97]	New Zealand	0–30	Cross-sectional	Prospective	All	HCWs	Adults	16.9	QFT-GIT	325	8	4
Fujita et al 2022[98]	Japan	0–30	Cohort	Prospective	All	Cancer	Adults	72.4	IGRA	123	4	4
Gabriele et al 2017[99]	Greece	0–30	Cohort	Prospective	All	IMID	Children	45.5	QFT-GIT	79	2	6
Gaensbauer et al 2018[100]	USA	0–30	Cohort	Retrospective	All	Children	Children	49.7	QFT-G	6683	53	5
Gaensbauer et al 2020[101]	USA	0–30	Cohort	Retrospective	All	Young children	Young children	43	QFT-GIT	113	3	6
Galindo et al 2019[102]	Colombia	31–100	Cross-sectional	NS	All	Cancer	Adults	63.1	QFT-GIT	149	1	6
Gao et al 2015[103]	China	31–100	Cohort	Prospective	All	Healthy people	All age	46	QFT-GIT	21022	600	6
Garazzino et al 2014[104]	Italy	0–30	Cohort	Retrospective	All	Young children	Young children	54.6	QFT-GIT	715	32	6
García-Gasalla et al 2013[105]	Spain	0–30	Cross-sectional	Prospective	All	IMID	Adults	50.4	QFT-GIT	123	4	6
Gebreegziabiher et al 2014[106]	Ethiopia	101–200	Cross-sectional	Prospective	All	Immunocompromised patients	Adults	0	QFT-GIT	85	14	6
Goletti et al 2020[107]	Italy	0–30	Cohort	Retrospective	All	HIV-positive	Adults	78.3	QFT-GIT	495	16	5
Goletti et al 2020[107]	Italy	0–30	Cohort	Retrospective	CD4: <100	HIV-positive	Adults	78.3	QFT-GIT	108	15	5
Goletti et al 2020[107]	Italy	0–30	Cohort	Retrospective	CD4: 100-200	HIV-positive	Adults	78.3	QFT-GIT	60	1	5
Goletti et al 2020[107]	Italy	0–30	Cohort	Retrospective	CD4: 200-500	HIV-positive	Adults	78.3	QFT-GIT	170	0	5
Goletti et al 2020[107]	Italy	0–30	Cohort	Retrospective	CD4: ≥500	HIV-positive	Adults	78.3	QFT-GIT	137	0	5
González-Moreno et al 2018[108]	Spain	0–30	Cohort	Retrospective	All	IMID	All age	46.7	QFT-GIT	520	13	6

Continue

	Area	TB burden	Study design	Prospective or retrospective	Subgroup	Population	Age group	Male (%)	Type of IGRA	Particip-ants (n)	Indeterm-inate (n)	Quadas score
Gonzślez-Moreno et al 2015[109]	Spain	0–30	Cohort	Prospective	All	Contacts	All age	44.1	QFT-GIT	272	9	6
Goodwin et al 2014[110]	USA	0–30	NS	NS	All	Healthy people	All age	84.09	QFT-GIT	2135	43	5
Grant et al 2012[111]	UK	0–30	NS	NS	All	Dialysis	Adults	NS	QFT-GIT	79	6	5
Grant et al 2012[111]	UK	0–30	NS	NS	All	Dialysis	Adults	NS	T-SPOT	79	7	5
Gray et al 2012[112]	USA	0–30	NS	Retrospective	All	HIV-positive	NS	NS	QFT-GIT	1364	27	6
Greenberg et al 2008[113]	USA	0–30	Cohort	Prospective	All	IMID	Adults	16.4	QFT	60	7	5
Greenberg et al 2008[113]	USA	0–30	Cohort	Prospective	All	Healthy people	Adults	23.8	QFT	40	1	5
Greveson et al 2013[114]	UK	0–30	Cohort	Retrospective	All	IMID	Adults	51.2	T-SPOT	125	4	5
Grimes et al 2007[115]	USA	0–30	Cross-sectional	NS	All	Drug and alcohol abusers	Adults	76	T-SPOT	119	3	6
Grinsdale et al 2016[116]	USA	0–30	Cohort	Retrospective	All	Children	Children	50	QFT-G	1092	77	6
Grinsdale et al 2016[116]	USA	0–30	Cohort	Retrospective	Age: 0-2y	Children	Children	50	QFT-G	56	11	6
Grinsdale et al 2016[116]	USA	0–30	Cohort	Retrospective	Age: 2-5y	Children	Children	50	QFT-G	236	17	6
Grinsdale et al 2016[116]	USA	0–30	Cohort	Retrospective	Age: 5-15y	Children	Children	50	QFT-G	800	49	6
Gunluoglu et al 2015[117]	Turkey	0–30	Cross-sectional	NS	All	Dialysis	NS	56	QFT-GIT	50	0	6
Hadaya et al 2013[118]	Switzerland	0–30	NS	Prospective	All	Transplant candidates	NS	58	QFT-GIT	205	5	6
Hakimian et al 2018[119]	USA	0–30	NS	Retrospective	All	IMID	Adults	57	QFT-GIT	107	24	4
Hand et al 2018[120]	USA	0–30	Cohort	Retrospective	All	Transplant candidates	Adults	61.48	QFT-G	148	20	5
Haustein et al 2009[121]	UK	0–30	Cohort	Retrospective	All	Children	Children	67	QFT-GIT	237	83	6
Haustein et al 2009[121]	UK	0–30	Cohort	Retrospective	Age: 0-5y	Children	Children	67	QFT-GIT	101	44	6
Haustein et al 2009[121]	UK	0–30	Cohort	Retrospective	Age: 5-15y	Children	Children	67	QFT-GIT	136	39	6
Haustein et al 2009[121]	UK	0–30	Cohort	Retrospective	Immunocompromised	Children	Children	67	QFT-GIT	59	39	6
Haustein et al 2009[121]	UK	0–30	Cohort	Retrospective	Immunocompetent	Children	Children	67	QFT-GIT	178	44	6
He et al 2012[122]	China	31–100	Cross-sectional	NS	All	HCWs	Adults	28	QFT-GIT	1005	6	6
He et al 2015[123]	China	31–100	Cohort	Prospective	All	HCWs	Adults	52.2	QFT-GIT	619	1	6
Helwig et al 2012[124]	Germany	0–30	NS	Prospective	All	IMID	NS	42.2	QFT-GIT	45	13	6
Hermansen et al 2014[125]	Denmark	0–30	NS	Retrospective	All	High risk	All age	47	QFT-GIT	15709	804	6

Continue

	Area	TB burden	Study design	Prospective or retrospective	Subgroup	Population	Age group	Male (%)	Type of IGRA	Particip -ants (n)	Indeterm -inate (n)	Quadas score
Hermansen et al 2014[125]	Denmark	0–30	NS	Retrospective	Age: 0-2y	High risk	All age	47	QFT-GIT	101	9	6
Hermansen et al 2014[125]	Denmark	0–30	NS	Retrospective	Age: 2-4y	High risk	All age	47	QFT-GIT	148	5	6
Hermansen et al 2014[125]	Denmark	0–30	NS	Retrospective	Age: 5-15y	High risk	All age	47	QFT-GIT	428	18	6
Hermansen et al 2014[125]	Denmark	0–30	NS	Retrospective	Adults	High risk	All age	47	QFT-GIT	355	14	6
Hermansen et al 2014[125]	Denmark	0–30	NS	Retrospective	Children	High risk	All age	47	QFT-GIT	28	1	6
Hesseling et al 2009[126]	South Africa	201–	Cross-sectional	NS	All	Contacts	All age	NS	QFT-G	82	3	6
Hesseling et al 2009[126]	South Africa	201–	Cross-sectional	NS	All	Contacts	All age	NS	T-SPOT	82	1	6
Hesseling et al 2009[126]	South Africa	201–	Cross-sectional	NS	Children	Contacts	Young children	NS	QFT-G	29	3	6
Hesseling et al 2009[126]	South Africa	201–	Cross-sectional	NS	Adults	Contacts	Young children	NS	QFT-G	53	0	6
Higuchi et al 2009[127]	Japan	0–30	Cohort	NS	All	Contacts	Children	50.6	QFT-GIT	308	2	6
Ho et al 2021[128]	USA	0–30	Cross-sectional	Prospective	All	High risk	All age	48.7	QFT-GIT	20788	91	6
Ho et al 2021[128]	USA	0–30	Cross-sectional	Prospective	All	High risk	All age	48.7	T-SPOT	20778	119	6
Hoffmann et al 2010[129]	Switzerland	0–30	Cross-sectional	NS	All	Dialysis	Adults	36.26	QFT-GIT	39	1	6
Hoffmann et al 2010[129]	Switzerland	0–30	Cross-sectional	NS	All	Healthy people	Adults	36.26	QFT-GIT	52	0	6
Hotta et al 2007[130]	Japan	0–30	Cross-sectional	NS	All	HCWs	Adults	36	QFT-GIT	207	5	6
Howley et al 2015[131]	USA	0–30	Cohort	Prospective	All	Children	Children	52.1	QFT-GIT	2520	13	6
Hradsky et al 2014[132]	Czech Republic	0–30	NS	NS	All	IMID	Children	49.5	QFT-GIT	165	16	6
Hradsky et al 2014[132]	Czech Republic	0–30	NS	NS	Immunocompromised	IMID	Children	49.5	QFT-GIT	86	12	6
Hradsky et al 2014[132]	Czech Republic	0–30	NS	NS	Immunocompetent	IMID	Children	49.5	QFT-GIT	63	4	6
Hsia et al 2012[133]	USA	0–30	NS	NS	All	IMID	Adults	34.2	QFT-GIT	2282	41	6
Hsiao et al 2017[134]	China, Taiwan	31–100	Cohort	Retrospective	All	IMID	Adults	75.34	QFT-GIT	134	7	6
Huang et al 2018[135]	China, Taiwan	31–100	Cohort	Retrospective	All	IMID	Adults	32.5	IGRA	382	32	4
Huang et al 2021[136]	China, Taiwan	31–100	Cohort	Prospective	All	Cancer	Adults	52.8	QFT-GIT	89	27	5
Huerga et al 2019[137]	Armenia	0–30	Cohort	Prospective	All	Contacts	Children	47	QFT-GIT	150	6	5
Hung et al 2015[138]	China, Taiwan	31–100	Cross-sectional	Prospective	All	HCWs	Adults	22.8	QFT-G	193	15	6
Hwang et al 2018[139]	South Korea	31–100	NS	NS	All	Healthy people	All age	22.4	QFT-GIT	23824	5	6
Igari et al 2017[140]	Japan	0–30	Cross-sectional	NS	All	IMID	Adults	18.2	QFT-plus	138	1	6

Continue

	Area	TB burden	Study design	Prospective or retrospective	Subgroup	Population	Age group	Male (%)	Type of IGRA	Particip -ants (n)	Indeterm -inate (n)	Quadas score
Igari et al 2017[140]	Japan	0–30	Cross-sectional	NS	All	IMID	Adults	18.2	T-SPOT	143	4	6
Igari et al 2019[141]	Japan	0–30	Cross-sectional	Prospective	All	Transplant candidates	Adults	59.6	QFT-plus	136	0	6
Igari et al 2019[141]	Japan	0–30	Cross-sectional	Prospective	All	Transplant candidates	Adults	59.6	T-SPOT	136	3	6
Igari et al 2021[142]	Japan	0–30	Cohort	Prospective	All	HIV-positive	Adults	87	QFT-plus	184	1	5
Igari et al 2021[142]	Japan	0–30	Cohort	Prospective	All	HIV-positive	Adults	87	T-SPOT	184	0	5
Inanc et al 2009[143]	Turkey	0–30	Cross-sectional	NS	All	IMID	NS	22	QFT-G	140	8	5
Ishikawa et al 2017[144]	Japan	0–30	Cross-sectional	Prospective	All	Transplant candidates	All age	60.9	QFT-GIT	92	2	6
Ishikawa et al 2017[144]	Japan	0–30	Cross-sectional	Prospective	All	Transplant candidates	All age	60.9	T-SPOT	92	2	6
Iwagaitsu et al 2016[145]	Japan	0–30	NS	NS	All	IMID	Adults	19.1	QFT-GIT	68	3	6
Iwagaitsu et al 2016[145]	Japan	0–30	NS	NS	All	IMID	Adults	19.1	T-SPOT	68	1	6
Jafri et al 2011[146]	USA	0–30	Cohort	Retrospective	All	Transplant candidates	Adults	59.66	QFT-GIT	119	15	6
Janssens et al 2008[147]	Switzerland	0–30	Cohort	Prospective	All	Contacts	All age	52	T-SPOT	295	15	6
Jones et al 2007[148]	USA	0–30	Cross-sectional	NS	All	HIV-positive	Adults	51.7	QFT-G	201	10	6
Jones et al 2007[148]	USA	0–30	Cross-sectional	Prospective	CD4: <100	HIV-positive	Adults	51.7	QFT-G	19	7	6
Jones et al 2007[148]	USA	0–30	Cross-sectional	Prospective	CD4: 100-199	HIV-positive	Adults	51.7	QFT-G	24	3	6
Jones et al 2007[148]	USA	0–30	Cross-sectional	Prospective	CD4: 200-499	HIV-positive	Adults	51.7	QFT-G	88	0	6
Jones et al 2007[148]	USA	0–30	Cross-sectional	Prospective	CD4: >500	HIV-positive	Adults	51.7	QFT-G	74	0	6
Jonnalagadda et al 2010[149]	USA	0–30	Cohort	NS	All	HIV-positive	All age	0	T-SPOT	333	52	6
Joshi et al 2014[150]	USA	0–30	Cohort	Retrospective	All	HCWs	Adults	53	QFT-GIT	2303	2	6
Jung et al 2012[151]	South Korea	31–100	NS	NS	All	IMID	All age	60.9	T-SPOT	269	11	5
Jung et al 2014[152]	South Korea	31–100	Cross-sectional	Retrospective	All	IMID	All age	50.1	QFT-GIT	631	43	6
Jung et al 2014[152]	South Korea	31–100	Cross-sectional	Retrospective	Immunocompromised	IMID	All age	50.1	QFT-GIT	9	3	6
Jung et al 2014[152]	South Korea	31–100	Cross-sectional	Retrospective	Immunocompetent	IMID	All age	50.1	QFT-GIT	616	36	6
Jung et al 2014[153]	South Korea	31–100	Cohort	NS	All	IMID	All age	51.9	T-SPOT	430	22	5
Jung et al 2015[154]	South Korea	31–100	Cohort	Prospective	All	IMID	All age	61.5	QFT-GIT	156	3	6
Jung et al 2015[154]	South Korea	31–100	Cohort	Prospective	All	IMID	All age	61.5	T-SPOT	156	6	6
Jung et al 2021[155]	South Korea	31–100	NS	NS	All	HCWs	Adults	35.7	IGRA	295	2	6
Juno et al 2016[156]	Canada	0–30	Cross-sectional	Prospective	All	Dialysis	Adults	53.75	QFT-GIT	80	1	6
Kabeer et al 2011[157]	India	101–200	Cohort	Prospective	All	HIV-positive	Adults	62	QFT-GIT	180	17	6

Continue

	Area	TB burden	Study design	Prospective or retrospective	Subgroup	Population	Age group	Male (%)	Type of IGRA	Particip-ants (n)	Indeterm-inate (n)	Quadas score
Kabeer et al 2011[157]	India	101–200	Cohort	Prospective	CD4: <100	HIV-positive	Adults	62	QFT-GIT	17	14	6
Kabeer et al 2011[157]	India	101–200	Cohort	Prospective	CD4: ≥100	HIV-positive	Adults	62	QFT-GIT	17	3	6
Kall et al 2012[158]	UK	0–30	Cohort	NS	All	HIV-positive	All age	43	T-SPOT	520	36	6
Kampmann et al 2018[159]	UK	0–30	Cohort	Prospective	All	Contacts	Children	51.9	IGRA	344	13	6
Kasambira et al 2011[160]	South Africa	201–	Cross-sectional	NS	All	Contacts	Children	48	QFT-GIT	254	18	6
Katsenos et al 2010[161]	Greece	0–30	Cross-sectional	Prospective	All	Healthy people	Adults	100	QFT-GIT	129	0	6
Katyal et al 2018[162]	USA	0–30	Cross-sectional	NS	All	Prisoners	All age	75.8	QFT-GIT	35090	160	6
Kaur et al 2018[163]	USA	0–30	Cohort	Retrospective	All	IMID	Adults	43.3	QFT-GIT	400	46	5
Kay et al 2019[164]	Eswatini	201–	Cohort	Prospective	All	Contacts	Children	59	QFT-GIT	46	2	6
Kay et al 2019[164]	Eswatini	201–	Cohort	Prospective	All	Contacts	Children	59	QFT-plus	46	1	6
Khawcharoenporn et al 2015[165]	Thailand	101–200	Cohort	NS	All	HIV-positive	Adults	53	QFT-GIT	150	0	6
Khawcharoenporn et al 2016[166]	Thailand	101–200	Cross-sectional	NS	All	HCWs	Adults	7.7	QFT-GIT	114	0	6
Kim et al 2009[167]	South Korea	31–100	Cohort	Retrospective	All	Immunocompromised patients	Adults	45.97	QFT-GIT	117	25	6
Kim et al 2009[167]	South Korea	31–100	Cohort	Retrospective	All	Healthy people	Adults	45.97	QFT-GIT	94	9	6
Kim et al 2010[168]	South Korea	31–100	Cohort	Prospective	All	Transplant candidates	Adults	NS	T-SPOT	209	25	6
Kim et al 2012[169]	South Korea	31–100	Cohort	Prospective	All	HIV-positive	Adults	93.3	T-SPOT	120	7	6
Kim et al 2014[170]	South Korea	31–100	Cohort	Retrospective	All	IMID	Adults	56.1	QFT-GIT	426	12	5
Kim et al 2017[171]	South Korea	31–100	Cohort	Prospective	All	HIV-positive	Adults	92	QFT-GIT	78	3	6
Kim et al 2017[171]	South Korea	31–100	Cohort	Prospective	CD4: <200	HIV-positive	Adults	92	QFT-GIT	8	2	6
Kim et al 2017[171]	South Korea	31–100	Cohort	Prospective	CD4: 200-350	HIV-positive	Adults	92	QFT-GIT	10	0	6
Kim et al 2017[171]	South Korea	31–100	Cohort	Prospective	CD4: ≥350	HIV-positive	Adults	92	QFT-GIT	60	1	6
Kim et al 2020[172]	South Korea	31–100	Cohort	Retrospective	All	Transplant candidates	Adults	58.9	QFT-GIT	1150	71	6
Kim et al 2021[173]	South Korea	31–100	NS	Retrospective	All	Children	Children	54.6	QFT-GIT	2037	156	5
Kim EU et al 2009[174]	South Korea	31–100	Cross-sectional	NS	All	Immunocompromised patients	Adults	44.6	QFT-GIT	117	25	6
Kim EU et al 2009[174]	South Korea	31–100	Cross-sectional	NS	All	Healthy people	Adults	44.6	QFT-GIT	94	9	6
Kim JH et al 2013[175]	South Korea	31–100	NS	Retrospective	All	IMID	Adults	14.5	QFT-GIT	745	21	6

Continue

	Area	TB burden	Study design	Prospective or retrospective	Subgroup	Population	Age group	Male (%)	Type of IGRA	Particip -ants (n)	Indeterm -inate (n)	Quadas score
Kim JH et al 2015[176]	South Korea	31–100	Cohort	Retrospective	All	IMID	Adults	65.8	QFT-GIT	582	22	6
Kim JS et al 2013[177]	Huang	31–100	Cohort	Prospective	All	Transplant candidates	Adults	22.6	QFT-GIT	126	6	6
Kim KH et al 2011[178]	South Korea	31–100	NS	NS	All	IMID	Adults	57.6	QFT-G	203	7	6
Kim SH et al 2011[179]	South Korea	31–100	Cohort	Prospective	All	Transplant candidates	Adults	56	T-SPOT	297	32	6
Kim SH et al 2015[180]	South Korea	31–100	Cohort	Prospective	All	Transplant candidates	Adults	58	T-SPOT	784	76	6
Kim SY et al 2013[181]	South Korea	31–100	Cohort	Prospective	All	Transplant candidates	Adults	54.5	QFT-GIT	126	6	6
Klein et al 2013[182]	Czech Republic	0–30	NS	NS	All	IMID	Adults	46.67	QFT-GIT	305	10	6
Kleinert et al 2012[183]	Germany	0–30	Cross-sectional	Prospective	All	IMID	NS	38.7	IGRA	1609	18	5
Kobashi et al 2007[184]	Japan	0–30	NS	Prospective	All	Immunocompromised patients	Adults	38.1	QFT-G	220	27	6
Kobashi et al 2010[185]	Japan	0–30	Cohort	Prospective	All	Contacts	Adults	33.8	QFT-G	130	5	5
Koesoemadinata et al 2017[186]	India	101–200	Cross-sectional	NS	All	Diabetes mellitus	All age	33.5	QFT-GIT	552	22	4
Korra et al 2019[187]	Egypt	0–30	Cohort	Prospective	All	Immunocompromised patients	Adults	78	QFT-GIT	50	2	6
Korra et al 2019[187]	Egypt	0–30	Cohort	Prospective	CD4: <200	Immunocompromised patients	Adults	78	QFT-GIT	4	2	6
Korra et al 2019[187]	Egypt	0–30	Cohort	Prospective	CD4: >200	Immunocompromised patients	Adults	78	QFT-GIT	11	0	6
Korra et al 2019[187]	Egypt	0–30	Cohort	Prospective	HIV-positive	Immunocompromised patients	Adults	78	QFT-GIT	15	2	6
Korra et al 2019[187]	Egypt	0–30	Cohort	Prospective	HIV-negative	Immunocompromised patients	Adults	78	QFT-GIT	35	0	6
Kurti et al 2015[188]	Hungary	0–30	Cross-sectional	Prospective	All	IMID	Adults	30.8	QFT-GIT	166	1	6
Kussen et al 2016[189]	Brazil	31–100	Cohort	Prospective	All	HIV-positive	Adults	47	QFT-GIT	140	0	5
Kwakernaak et al 2011[190]	Netherlands	0–30	Cohort	Prospective		IMID	All age	53.6	QFT-G	56	0	5
LaCourse et al 2017[191]	Kenya	201–	Cohort	Prospective	All	HIV-positive	Adults	0	QFT-GIT	100	16	6
Lai et al 2019[192]	China, Taiwan	31–100	Cohort	Retrospective	All	IMID	Adults	75	QFT-GIT	130	10	4
Lange et al 2012[193]	Germany	0–30	Cohort	Retrospective	All	Immunocompromised patients	Adults	57	QFT-GIT	460	62	6
Latorre et al 2010[194]	Spain	0–30	NS	NS	All	HIV-positive	Adults	71.7	QFT-GIT	75	2	6
Latorre et al 2010[194]	Spain	0–30	NS	NS	All	HIV-positive	Adults	71.7	T-SPOT	75	2	6
Latorre et al 2014[195]	Spain	0–30	Cohort	Prospective	All	IMID	Adults	67	QFT-GIT	103	2	6

Continue

	Area	TB burden	Study design	Prospective or retrospective	Subgroup	Population	Age group	Male (%)	Type of IGRA	Particip -ants (n)	Indeterm -inate (n)	Quadas score
Latorre et al 2014[195]	Spain	0–30	Cohort	Prospective	All	IMID	Adults	67	T-SPOT	103	7	6
Lebina et al 2015[196]	South Africa	201–	Cross-sectional	NS	All	Children	Children	NS	QFT-GIT	2115	10	5
Lee H et al 2015[197]	South Korea	31–100	Cohort	Prospective	All	IMID	Adults	55.6	QFT-GIT	368	13	6
Lee JH et al 2014[198]	South Korea	31–100	NS	NS	All	IMID	Adults	9.4	QFT-G	64	11	6
Lee JH et al 2014[198]	South Korea	31–100	NS	NS	All	Healthy people	Adults	9.4	QFT-G	79	0	6
Lee K et al 2009[199]	South Korea	31–100	Cohort	Prospective	All	HCWs	Adults	0	QFT-GIT	196	0	5
Lee SH et al 2010[200]	South Korea	31–100	Cohort	Prospective	All	Contacts	Adults	65.4	QFT-GIT	75	1	6
Lee SSJ et al 2009[201]	China, Taiwan	31–100	Cohort	Prospective	All	Dialysis	Adults	62.5	QFT-G	32	2	5
Lee SSJ et al 2010[202]	Taiwan, China	31–100	Cross-sectional	NS	All	Dialysis	Adults	NS	QFT-G	93	10	5
Lee SSJ et al 2015[203]	China, Taiwan	31–100	Cohort	Prospective	All	HIV-positive	Adults	96.4	QFT-G	772	31	6
Lee YM et al 2014[204]	South Korea	31–100	Cohort	Prospective	All	Transplant candidates	Adults	53.2	QFT-GIT	391	41	6
Legesse et al 2011[205]	Ethiopia	101–200	Cross-sectional	NS	All	Healthy people	Adults	58.9	QFT-G	570	6	6
Leidl et al 2010[206]	Uganda	101–200	Cohort	NS	All	HIV-positive	Adults	25.8	QFT-GIT	109	4	5
Leidl et al 2010[206]	Uganda	101–200	Cohort	NS	All	HIV-positive	Adults	25.8	T-SPOT	109	4	5
Lempp et al 2017[207]	USA	0–30	Cohort	NS	All	Healthy people	Adults	NS	QFT-GIT	792	5	6
Lempp et al 2017[207]	USA	0–30	Cohort	NS	All	Healthy people	Adults	NS	QFT-G	792	16	6
Lien et al 2009[208]	Viet Nam	101–200	Cross-sectional	NS	All	HCWs	Adults	25.7	QFT-GIT	300	35	6
Lighter et al 2009[209]	USA	0–30	NS	Prospective	All	Children	Children	58	QFT-G	207	3	5
Lighter-Fisher et al 2012[210]	USA	0–30	Cohort	Retrospective	All	Pregnants	Adults	0	QFT-GIT	140	9	6
Lighter-Fisher et al 2012[210]	USA	0–30	Cohort	Retrospective	All	Healthy people	Adults	0	QFT-GIT	140	4	6
Lin et al 2016[211]	China, Taiwan	31–100	Cross-sectional	Prospective	All	HIV-positive	Adults	91.9	QFT-G	86	8	6
Lin et al 2016[211]	China, Taiwan	31–100	Cross-sectional	Prospective	All	HIV-positive	Adults	91.9	QFT-GIT	86	2	6
Lin et al 2016[211]	China, Taiwan	31–100	Cross-sectional	Prospective	CD4: <200	HIV-positive	Adults	91.9	QFT-GIT	22	2	6
Lin et al 2016[211]	China, Taiwan	31–100	Cross-sectional	Prospective	CD4: ≥200	HIV-positive	Adults	91.9	QFT-GIT	64	0	6
Lin et al 2022[212]	China, Taiwan	31–100	Cohort	Retrospective	All	HIV-positive	Adults	96	QFT-GIT	7951	38	6
Lombardi et al 2015[213]	Italy	0–30	Cohort	Retrospective	All	IMID	Adults	64.36	QFT-GIT	188	4	6
Lombardi et al 2018[214]	Italy	0–30	NS	Retrospective	All	Children	Children	55.7	QFT-GIT	159	18	6
Lombardi et al 2018[214]	Italy	0–30	NS	Retrospective	All	Contacts	Children	55.7	QFT-GIT	245	1	6

Continue

	Area	TB burden	Study design	Prospective or retrospective	Subgroup	Population	Age group	Male (%)	Type of IGRA	Particip -ants (n)	Indeterm -inate (n)	Quadas score
Lombardi et al 2018[214]	Italy	0–30	NS	Retrospective	All	Healthy people	Children	55.7	QFT-GIT	30	1	6
Loutet et al 2018[215]	UK	0–30	Cohort	Retrospective	All	Refugees and immigrants	All age	54.2	IGRA	2269	5	3
Lucas et al 2010[216]	Australia	0–30	Cohort	Prospective	All	Children	Children	47.86	QFT-GIT	460	70	6
Lucas et al 2010[216]	Australia	0–30	Cohort	Prospective	All	Children	Children	47.86	T-SPOT	420	8	6
Lucet et al 2015[217]	France	0–30	Cross-sectional	Prospective	All	HCWs	Adults	NS	QFT-GIT	807	7	6
Luetkemeyer et al 2007[218]	USA	0–30	Cohort	Prospective	All	HIV-positive	Adults	78	QFT-GIT	294	15	5
Luetkemeyer et al 2007[218]	USA	0–30	Cohort	Prospective	CD4: <100	HIV-positive	Adults	78	QFT-GIT	31	5	5
Luetkemeyer et al 2007[218]	USA	0–30	Cohort	Prospective	CD4: 100–350	HIV-positive	Adults	78	QFT-GIT	111	4	5
Luetkemeyer et al 2007[218]	USA	0–30	Cohort	Prospective	CD4: ≥350	HIV-positive	Adults	78	QFT-GIT	152	6	5
Machado et al 2009[219]	Brazil	31–100	Cross-sectional	NS	All	Contacts	All age	40	QFT-GIT	301	3	5
Maden et al 2011[220]	Turkey	0–30	Cohort	Prospective	All	Dialysis	Adults	51.04	QFT-G	96	3	5
Mahmoudi et al 2020[221]	Iran	0–30	NS	NS	All	Transplant candidates	Children	51.2	QFT-GIT	84	11	5
Mahomed et al 2011[222]	South Africa	201–	Cohort	Prospective	All	Healthy people	NS	45.8	QFT-GIT	6363	13	5
Mancuso et al 2012[223]	USA	0–30	Cross-sectional	NS	All	Healthy people	Adults	65.5	QFT-GIT	1850	17	6
Mancuso et al 2016[224]	USA	0–30	Cross-sectional	NS	All	Healthy people	NS	50	QFT-GIT	6083	19	5
Mandalakas et al 2008[225]	South Africa	201–	Cross-sectional	NS	All	HIV-positive	Adults	NS	QFT-G	32	3	6
Mandalakas et al 2008[225]	South Africa	201–	Cross-sectional	NS	All	HIV-positive	Adults	NS	T-SPOT	43	2	6
Mandalakas et al 2015[226]	South Africa	201–	Cohort	Prospective	All	Children	Children	51	QFT-GIT	1044	20	6
Mandalakas et al 2015[226]	South Africa	201–	Cohort	Prospective	All	HIV-positive	Children	51	T-SPOT	297	1	6
Mandalakas et al 2015[226]	South Africa	201–	Cohort	Prospective	HIV-positive	HIV-positive	Children	51	QFT-GIT	299	14	6
Mandalakas et al 2015[226]	South Africa	201–	Cohort	Prospective	HIV-negative	HIV-negaitive	Children	51	QFT-GIT	745	6	6
Mantri et al 2021[227]	India	101–200	NS	Prospective	All	IMID	Adults	55	QFT-GIT	131	6	6
Mantri et al 2021[227]	India	101–200	NS	Prospective	All	Healthy people	Adults	65	QFT-GIT	126	5	6
Manuel et al 2007[228]	USA	0–30	NS	Prospective	All	Transplant candidates	Adults	79.73	QFT-G	153	12	6
Mardani et al 2010[229]	Iran	0–30	Cross-sectional	NS	All	HIV-positive	Adults	86	QFT-G	50	11	5
Mardani et al 2010[229]	Iran	0–30	Cross-sectional	NS	CD4: >500	HIV-positive	Adults	86	QFT-G	3	0	5
Mardani et al 2010[229]	Iran	0–30	Cross-sectional	NS	CD4: 350–500	HIV-positive	Adults	86	QFT-G	10	2	5
Mardani et al 2010[229]	Iran	0–30	Cross-sectional	NS	CD4: 200–350	HIV-positive	Adults	86	QFT-G	23	3	5

Continue

	Area	TB burden	Study design	Prospective or retrospective	Subgroup	Population	Age group	Male (%)	Type of IGRA	Particip -ants (n)	Indeterm -inate (n)	Quadas score
Mardani et al 2010[229]	Iran	0–30	Cross-sectional	NS	CD4: <200	HIV-positive	Adults	86	QFT-G	14	6	5
Mariette et al 2012[230]	France	0–30	Cohort	Prospective	All	IMID	Adults	NS	QFT-GIT	392	11	6
Mariette et al 2012[230]	France	0–30	Cohort	Prospective	All	IMID	Adults	NS	T-SPOT	392	25	6
Marino et al 2017[231]	Italy	0–30	NS	NS	All	IMID	Children	NS	QFT-G	120	4	3
Maritsi et al 2011[232]	USA	0–30	Cohort	Retrospective	All	IMID	Children	52.17	QFT-GIT	23	2	5
Marquez et al 2016[233]	Uganda	101–200	Cross-sectional	NS	All	Young children	Young children	NS	QFT-GIT	208	15	5
Martínez-López et al 2021[234]	Spain	0–30	Cross-sectional	NS	All	IMID	Adults	NS	QFT-plus	1117	175	3
Martínez-Morillo et al 2013[235]	Spain	0–30	Cohort	Prospective	All	IMID	Adults	33.96	T-SPOT	53	2	5
Martyn-Simmons et al 2013[236]	UK	0–30	Cross-sectional	Prospective	All	IMID	Adults	58.57	T-SPOT	70	3	6
Mathad et al 2014[237]	USA	0–30	Cross-sectional	Prospective	All	Pregnants	Adults	0	QFT-GIT	154	5	6
Mathad et al 2016[238]	India	101–200	NS	NS	All	HIV-positive	Adults	0	QFT-GIT	149	4	6
Matsumura et al 2016[239]	Japan	0–30	Cohort	Prospective	All	IMID	Adults	25.2	QFT-GIT	230	12	5
Matsumura et al 2016[239]	Japan	0–30	Cohort	Prospective	All	IMID	Adults	25.2	T-SPOT	230	5	5
Matulis et al 2008[240]	Switzerland	0–30	NS	Prospective	All	IMID	All age	50	QFT-GIT	142	8	5
Matulis et al 2008[240]	Switzerland	0–30	NS	Prospective	Immunocompromised	IMID	All age	50	QFT-GIT	126	7	5
Matulis et al 2008[240]	Switzerland	0–30	NS	Prospective	Immunocompetent	IMID	All age	50	QFT-GIT	16	1	5
Mehta et al 2015[241]	USA	0–30	Cohort	Retrospective	All	IMID	Adults	23	QFT-G	134	2	5
Meinerz et al 2021[242]	Brazil	31–100	Cohort	Retrospective	All	Transplant candidates	Adults	34.5	QFT-GIT	122	6	6
Méndez-Echevarría et al 2012[243]	Spain	0–30	Cohort	Prospective	All	Children	Children	15	QFT-GIT	453	20	4
Mensah et al 2017[244]	UK	0–30	NS	NS	All	Contacts	All age	46	QFT-GIT	100	3	5
Milman et al 2011[245]	Denmark	0–30	Cohort	Prospective	All	IMID	Adults	50	QFT-GIT	44	3	5
Mínguez et al 2012[246]	Spain	0–30	Cross-sectional	NS	All	IMID	NS	34	QFT-GIT	53	4	5
Mínguez et al 2012[246]	Spain	0–30	Cross-sectional	NS	All	IMID	NS	34	T-SPOT	53	2	5
Miranda et al 2009[247]	USA	0–30	NS	NS	All	HCWs	NS	NS	QFT-GIT	1528	168	5
Miranda et al 2009[247]	USA	0–30	NS	NS	All	Healthy people	Adults	NS	QFT-GIT	834	51	5
Molina et al 2016[248]	USA	0–30	Cohort	Prospective	All	Pregnants	Adults	0	T-SPOT	138	9	6

Continue

	Area	TB burden	Study design	Prospective or retrospective	Subgroup	Population	Age group	Male (%)	Type of IGRA	Particip -ants (n)	Indeterm -inate (n)	Quadas score
Moon et al 2011[249]	South Korea	31–100	Cross-sectional	NS	All	HCWs	Adults	41	QFT-GIT	173	0	5
Moon et al 2013[250]	South Korea	31–100	Cohort	Prospective	All	Transplant candidates	Adults	56	QFT-GIT	244	34	5
Moon et al 2013[251]	South Korea	31–100	Cross-sectional	Prospective	All	Transplant candidates	Adults	63	T-SPOT	205	15	4
Moon et al 2017[252]	South Korea	31–100	NS	Retrospective	All	Transplant candidates	All age	80.5	QFT-GIT	270	16	5
Moon et al 2017[253]	USA	0–30	Cross-sectional	Prospective	All	HCWs	Adults	30.4	QFT-plus	989	2	5
Moucaut et al 2013[254]	France	0–30	Cross-sectional	Retrospective	All	HCWs	Adults	78.7	QFT-GIT	637	3	6
Mukai et al 2017[255]	Japan	0–30	Cohort	Retrospective	All	HCWs	Adults	21.4	QFT-GIT	140	8	5
Muñoz et al 2015[256]	Spain	0–30	Cohort	Prospective	All	Transplant candidates	Adults	76	QFT-GIT	50	2	4
Nkurunungi et al 2012[257]	Ugandan	101–200	Cross-sectional	Prospective	All	Children	Children	52.4	T-SPOT	546	44	6
Nozawa et al 2016[258]	Japan	0–30	Cohort	Retrospective	All	IMID	All age	37.5	QFT-GIT	81	8	6
Nozawa et al 2016[258]	Japan	0–30	Cohort	Retrospective	All	IMID	All age	37.5	T-SPOT	27	0	6
Ogawa et al 2021[259]	Japan	0–30	Cross-sectional	NS	All	Dialysis	Adults	81	QFT-G	118	7	5
Okada et al 2008[260]	Cambodia	101–200	NS	NS	All	Contacts	Children	57	QFT-G	204	9	4
Oliveira et al 2017[261]	Brazil	31–100	Cross-sectional	NS	All	HIV-positive	Adults	50	QFT-GIT	81	22	5
Oliveira et al 2017[261]	Brazil	31–100	Cross-sectional	NS	CD4: <200	HIV-positive	Adults	50	QFT-GIT	13	7	5
Oliveira et al 2017[261]	Brazil	31–100	Cross-sectional	NS	CD4: ≥200	HIV-positive	Adults	50	QFT-GIT	62	14	5
Pai et al 2005[262]	India	101–200	Cross-sectional	NS	All	HCWs	Adults	48	QFT-GIT	724	1	6
Pai et al 2009[263]	India	101–200	Cohort	Prospective	All	Contacts	All age	43	QFT-GIT	250	0	4
Paluch-Oleś et al 2013[264]	Poland	0–30	Cross-sectional	NS	All	IMID	Adults	25.6	QFT-GIT	90	3	5
Paluch-Oleś et al 2013[264]	Poland	0–30	Cross-sectional	NS	All	Healthy people	Adults	25.6	QFT-GIT	20	0	5
Papay et al 2011[265]	Austria	0–30	Cohort	Prospective	All	IMID	Adults	48.9	QFT-GIT	190	26	6
Papay et al 2011[265]	Austria	0–30	Cohort	Prospective	Immunocompromised	IMID	Adults	48.9	QFT-GIT	137	23	6
Papay et al 2011[265]	Austria	0–30	Cohort	Prospective	Immunocompetent	IMID	Adults	48.9	QFT-GIT	53	3	6
Pareek et al 2013[266]	UK	0–30	Cohort	Prospective	All	Refugees and immigrants	All age	35.5	QFT-GIT	229	2	4
Pareek et al 2013[266]	UK	0–30	Cohort	Prospective	All	Refugees and immigrants	All age	35.5	T-SPOT	229	7	4
Park et al 2009[267]	South Korea	31–100	NS	NS	All	IMID	All age	44.2	QFT-GIT	56	2	5

Continue

	Area	TB burden	Study design	Prospective or retrospective	Subgroup	Population	Age group	Male (%)	Type of IGRA	Particip-ants (n)	Indeterm-inate (n)	Quadas score
Park et al 2010[268]	South Korea	31–100	Cohort	Prospective	All	HCWs	Adults	15	QFT-GIT	322	3	5
Park et al 2020[269]	South Korea	31–100	NS	Retrospective	All	Dialysis	Adults	58	QFT-GIT	1162	70	4
Parrella et al 2015[270]	Italy	0–30	Cohort	NS	All	HIV-positive	Adults	89.8	QFT-GIT	98	4	6
Parrella et al 2015[270]	Italy	0–30	Cohort	NS	All	HIV-positive	Adults	89.8	T-SPOT	98	13	6
Parrella et al 2015[270]	Italy	0–30	Cohort	NS	CD4: <100	HIV-positive	Adults	89.8	QFT-GIT	18	1	6
Parrella et al 2015[270]	Italy	0–30	Cohort	NS	CD4: 100-200	HIV-positive	Adults	89.8	QFT-GIT	9	1	6
Parrella et al 2015[270]	Italy	0–30	Cohort	NS	CD4: 200-300	HIV-positive	Adults	89.8	QFT-GIT	14	1	6
Parrella et al 2015[270]	Italy	0–30	Cohort	NS	CD4: ≥301	HIV-positive	Adults	89.8	QFT-GIT	57	1	6
Passalent et al 2007[271]	Canada	0–30	Cross-sectional	Prospective	All	Dialysis	NS	0	T-SPOT	203	14	5
Pavić et al 2011[272]	Croatia	0–30	NS	NS	All	Contacts	Children	59.9	QFT-GIT	142	1	5
Pavić et al 2015[273]	Croatia	0–30	Cohort	Prospective	All	Young children	Young children	56.1	QFT-GIT	171	2	5
Perez-Porcuna et al 2016[274]	Brazil	31–100	Cross-sectional	NS	All	Young children	Young children	41.9	QFT-GIT	121	16	6
Perifanou et al 2018[275]	Greece	0–30	Cross-sectional	Retrospective	All	IMID	NS	52.5	QFT-GIT	158	4	5
Perry et al 2008[276]	USA	0–30	Cohort	Prospective	All	Contacts	All age	41	QFT-GIT	88	1	6
Person et al 2010[277]	USA	0–30	Cross-sectional	Retrospective	All	Contacts	Adults	43.2	QFT-GIT	313	1	3
Petrucci et al 2008[278]	Nepal	201–	Cross-sectional	NS	All	Contacts	Children	46	QFT-GIT	146	4	5
Petrucci et al 2008[278]	Brazil	31–100	Cross-sectional	NS	All	Contacts	Children	48.7	QFT-GIT	113	2	5
Piana et al 2006[279]	Italy	0–30	Cross-sectional	NS	All	Cancer	Adults	47.8	T-SPOT	138	6	6
Ponce de Leon et al 2008[280]	Peru	101–200	NS	NS	All	IMID	Adults	9.9	QFT-GIT	103	2	6
Porsa et al 2007[281]	USA	0–30	Cross-sectional	NS	All	Prisoners	Adults	84.87	QFT-GIT	412	22	6
Pratt et al 2007[282]	UK	0–30	Cohort	Prospective	All	IMID	Adults	1	QFT-G	101	10	3
Prignano et al 2014[283]	Italy	0–30	Cross-sectional	NS	All	IMID	Adults	59.4	QFT-GIT	267	1	5
Primaturia et al 2020[284]	Indonesia	201–	Cross-sectional	Prospective	All	Immunocompromised patients	Children	47.9	QFT-plus	71	1	5
Pullar et al 2014[285]	Norway	0–30	Cross-sectional	NS	All	HIV-positive	Adults	46	QFT-GIT	298	3	5
Pullar et al 2014[285]	Norway	0–30	Cross-sectional	NS	All	HIV-positive	Adults	46	T-SPOT	117	0	5

Continue

	Area	TB burden	Study design	Prospective or retrospective	Subgroup	Population	Age group	Male (%)	Type of IGRA	Particip -ants (n)	Indeterm -inate (n)	Quadas score
Pullar et al 2014[285]	Norway	0–30	Cross-sectional	NS	CD4: <200	HIV-positive	Adults	46	QFT-GIT	32	3	5
Pullar et al 2014[285]	Norway	0–30	Cross-sectional	NS	CD4: >200	HIV-positive	Adults	46	QFT-GIT	40	0	5
Qumseya et al 2011[286]	USA	0–30	Cohort	Retrospective	All	IMID	Adults	45.59	QFT-G	340	9	5
Rafiza et al 2011[287]	Malaysia	31–100	Cross-sectional	NS	All	HCWs	Adults	12	QFT-GIT	954	1	5
Ramos et al 2012[288]	Spain	0–30	Cohort	Prospective	All	HIV-positive	Adults	76.9	QFT-GIT	373	10	5
Ramos et al 2012[288]	Spain	0–30	Cohort	Prospective	All	HIV-positive	Adults	76.9	T-SPOT	373	26	5
Ramos et al 2013[289]	Spain	0–30	Cross-sectional	Prospective	All	IMID	All age	32.1	QFT-G	153	1	5
Ranaivomanana et al 2015[290]	Madagascar	201–	Cross-sectional	NS	All	Children	Children	50	T-SPOT	142	22	5
Rangaka et al 2015[291]	South Africa	201–	RCT	Prospective	All	HIV-positive	Adults	24.9	QFT-GIT	1329	74	6
Rangeaka et al 2007[292]	South Africa	201–	Cross-sectional	NS	All	HIV-positive	Adults	46	QFT-G	100	7	5
Rangeaka et al 2007[292]	South Africa	201–	Cross-sectional	NS	All	HIV-positive	Adults	46	T-SPOT	100	1	5
Rangeaka et al 2007[292]	South Africa	201–	Cross-sectional	NS	All	Healthy people	Adults	48	QFT-G	99	2	5
Rangeaka et al 2007[292]	South Africa	201–	Cross-sectional	NS	All	Healthy people	Adults	48	T-SPOT	100	0	5
Ribeiro-Rodrigues et al 2014[293]	Brazil	31–100	Cohort	Prospective	All	Contacts	All age	40.6	QFT-GIT	467	1	5
Richeldi et al 2008[294]	Italy	0–30	NS	Retrospective	All	Contacts	Children	50	QFT-G	70	10	3
Richeldi et al 2008[294]	Italy	0–30	NS	Retrospective	All	Contacts	Children	39.5	QFT-GIT	81	10	3
Richeldi et al 2009[295]	Italy	0–30	NS	NS	All	Immunocompromised patients	Adults	54.7	QFT-G	331	24	6
Richeldi et al 2009[295]	Italy	0–30	NS	NS	All	Immunocompromised patients	Adults	54.7	T-SPOT	331	2	6
Ringshausen et al 2009[296]	Germany	0–30	Cross-sectional	Prospective	All	HCWs		30.8	QFT-GIT	144	1	6
Ringshausen et al 2010[297]	German	0–30	Cohort	Prospective	All	HCWs	Adults	29.1	QFT-GIT	182	2	6
Rivas et al 2009[298]	Spain	0–30	Cross-sectional	Prospective	All	Drug and alcohol abusers	NS	83.7	QFT-GIT	57	2	5
Romanowski et al 2020[299]	Canada	0–30	Cohort	Retrospective	All	Dialysis	Adults	62.29	IGRA	1790	37	3
Rose et al 2015[300]	Canada	0–30	Cross-sectional	NS	All	Contacts	Children	52	QFT-GIT	230	5	6
Roth et al 2016[301]	USA	0–30	NS	Retrospective	All	Transplant candidates	Adults	62	IGRA	280	37	6
Rousset et al 2020[302]	France	0–30	NS	Retrospective	All	IMID	Adults	56.7	QFT-GIT	388	33	5

Continue

	Area	TB burden	Study design	Prospective or retrospective	Subgroup	Population	Age group	Male (%)	Type of IGRA	Particip -ants (n)	Indeterm -inate (n)	Quadas score
Ruhwald et al 2008[303]	Denmark	0–30	NS	NS	All	Contacts	Children	39	QFT-GIT	97	19	5
Ruhwald et al 2008[303]	Denmark	0–30	NS	NS	All	Healthy people	Children	52	QFT-GIT	23	6	5
Ruhwald et al 2017[304]	Spain	0–30	RCT	Prospective	All	Contacts	All age	54	QFT-GIT	618	4	6
Ruhwald et al 2017[304]	Spain	0–30	RCT	Prospective	All	Healthy people	All age	28	QFT-GIT	263	0	6
Rutherford et al 2012[305]	Netherlands	0–30	NS	NS	All	Contacts	Children	49	QFT-GIT	371	10	6
Ryu et al 2018[306]	South Korea	31–100	NS	Prospective	All	Immunocompromised patients	Adults	64.7	QFT-GIT	317	10	5
Ryu et al 2018[306]	South Korea	31–100	NS	Prospective	All	Immunocompromised patients	Adults	64.7	QFT-plus	317	10	5
Sabri et al 2019[307]	Morocco	31–100	Cross-sectional	NS	All	HCWs	Adults	54	QFT-GIT	662	31	6
Said et al 2019[308]	Tanzania	201–	Cohort	Prospective	All	Children	Children	48.17	QFT-GIT	301	9	4
Saidenberg-Kermanac'h et al 2012[309]	France	0–30	NS	NS	All	IMID	Adults	57	QFT-GIT	123	3	4
Sandhu et al 2020[310]	UK	0–30	Cohort	Prospective	All	HIV-positive	Adults	31.85	T-SPOT	128	4	4
Santin et al 2011[311]	Spain	0–30	Cross-sectional	Prospective	All	HIV-positive	Adults	77.8	QFT-GIT	135	2	5
Santin et al 2011[311]	Spain	0–30	Cross-sectional	Prospective	CD4: <100	HIV-positive	Adults	77.8	QFT-GIT	21	2	5
Santin et al 2011[311]	Spain	0–30	Cross-sectional	Prospective	CD4: >100	HIV-positive	Adults	77.8	QFT-GIT	114	0	5
Sauzullo et al 2010[312]	Italy	0–30	Cross-sectional	NS	All	IMID	Adults	59	QFT-GIT	195	26	5
Sauzullo et al 2010[312]	Italy	0–30	Cross-sectional	NS	All	HIV-positive	Adults	59	QFT-GIT	207	40	5
Sauzullo et al 2010[312]	Italy	0–30	Cross-sectional	NS	CD4: <200	HIV-positive	Adults	59	QFT-GIT	40	27	5
Sauzullo et al 2010[312]	Italy	0–30	Cross-sectional	NS	CD4: >200	HIV-positive	Adults	59	QFT-GIT	155	13	5
Sauzullo et al 2013[313]	Italy	0–30	NS	Prospective	All	IMID	Adults	34.5	QFT-GIT	148	6	5
Sauzullo et al 2014[314]	Italy	0–30	Cross-sectional	NS	All	HCWs	Adults	49.5	QFT-GIT	196	7	5
Savaj et al 2014[315]	Iran	0–30	Cross-sectional	NS	All	Dialysis	Adults	66	QFT-G	102	18	3
Sayyahfar et al 2020[316]	Iran	0–30	Cross-sectional	NS	All	Transplant candidates	Children	48	QFT-GIT	24	0	4
Schablon et al 2010[317]	Germany	0–30	Cross-sectional	NS	All	HCWs	Adults	38	QFT-GIT	2028	24	6
Schoepfer et al 2018[318]	Switzerland	0–30	Cohort	Prospective	All	IMID	All age	50	QFT-GIT	168	5	4
Scordo et al 2021[319]	USA	0–30	Cross-sectional	NS	All	Contacts	Adults	NS	IGRA	308	9	5
Scrivo et al 2012[320]	Italy	0–30	Cohort	Prospective	All	IMID	Adults	31.1	QFT-GIT	119	21	5

Continue

	Area	TB burden	Study design	Prospective or retrospective	Subgroup	Population	Age group	Male (%)	Type of IGRA	Particip -ants (n)	Indeterm -inate (n)	Quadas score
Scrivo et al 2013[321]	Italy	0–30	Cohort	Prospective	All	IMID	Adults	34.3	QFT-GIT	102	18	6
Scrivo et al 2022[322]	Italy	0–30	NS	Prospective	All	IMID	Adults	20.7	IGRA	29	5	6
Sester et al 2014[323]	European countries	0–30	Cohort	Prospective	All	Immunocompromised patients	Adults	40.73	QFT-GIT	1532	109	4
Sester et al 2014[323]	European countries	0–30	Cross-sectional	Prospective	All	Immunocompromised patients	Adults	40.73	T-SPOT	1503	125	4
Shah et al 2011[324]	South Africa	201–	Cohort	Prospective	All	Contacts	Children	48	QFT-GIT	196	17	5
Shanaube et al 2011[325]	Zambia and South Africa	201–	Cross-sectional	NS	All	Contacts	Adults	30	QFT-GIT	1997	194	6
Sharninghausen et al 2018[326]	USA	0–30	Cohort	Retrospective	All	High risk	Adults	44	QFT-GIT	3128	118	6
Sharninghausen et al 2018[326]	USA	0–30	Cohort	Retrospective	HIV-positive	High risk	Adults	44	QFT-GIT	319	15	6
Sharninghausen et al 2018[326]	USA	0–30	Cohort	Retrospective	HIV-negative	High risk	Adults	44	QFT-GIT	2809	103	6
Shin et al 2017[327]	South Korea	31–100	Cohort	Retrospective	All	Diabetes mellitus	Adults	55	QFT-GIT	3391	266	6
Shovman et al 2009[328]	Israel	0–30	NS	NS	All	IMID	Adults	NS	QFT-G	35	10	6
Shovman et al 2009[328]	Israel	0–30	NS	NS	All	Healthy people	Adults	NS	QFT-G	15	0	6
Shu et al 2012[329]	China, Taiwan	31–100	Cross-sectional	Prospective	All	Dialysis	Adults	53	QFT-GIT	427	20	6
Shu et al 2013[330]	China, Taiwan	31-100	Cohort	Prospective	All	Dialysis	Adults	55	QFT-GIT	204	4	6
Shu et al 2015[331]	China, Taiwan	31–100	Cross-sectional	Prospective	All	Dialysis	Adults	51	QFT-GIT	488	17	6
Shu et al 2016[332]	China, Taiwan	31-100	Cohort	Prospective	All	Dialysis	Adults	53	QFT-GIT	940	34	6
Shu et al 2020[333]	China, Taiwan	31–100	Cohort	Prospective	All	Transplant candidates	Adults	60	QFT-GIT	305	19	6
Silveira et al 2018[334]	Brazil	31–100	Cohort	NS	All	Children	Children	45	T-SPOT	86	13	6

Continue

	Area	TB burden	Study design	Prospective or retrospective	Subgroup	Population	Age group	Male (%)	Type of IGRA	Particip -ants (n)	Indeterm -inate (n)	Quadas score
Silveira et al 2018[334]	Brazil	31–100	Cohort	NS	Immunocompromised	Immunocompromised patients	Children	45	T-SPOT	45	9	6
Silveira et al 2018[334]	Brazil	31–100	Cohort	NS	Immunocompetent	Immunocompetent group	Children	45	T-SPOT	41	4	6
Simpson et al 2013[335]	USA	0–30	Cohort	Retrospective	All	Refugees and immigrants	All age	49	QFT-GIT	541	8	6
Slater et al 2013[336]	USA	0–30	Cohort	Retrospective	All	HCWs	Adults	72	QFT-GIT	9153	113	6
Soborg et al 2010[337]	Denmark	0–30	Cohort	Retrospective	All	Children	Children	50.4	QFT-G	2218	22	5
Soborg et al 2009[338]	Denmark	0–30	Cohort	Prospective	All	IMID	Adults	38	QFT-G	294	13	6
Sollai et al 2017[339]	Italy	0–30	Cross-sectional	Prospective	All	Children	Children	40.4	QFT-GIT	1355	0	6
Song et al 2014[340]	South Korea	31–100	Cohort	Prospective	All	Contacts	All age	54.5	QFT-GIT	2982	16	6
Sosa-Moreno et al 2020[341]	USA	0–30	Cohort	Retrospective	All	Transplant candidates	Adults	61	QFT-GIT	267	52	4
Sousa et al 2019[342]	Portugal	0–30	NS	NS	All	IMID	Adults	44	IGRA	23	7	5
Southern et al 2019[343]	UK	0-30	Cohort	Retrospective	All	Contacts	Adults	97.6	QFT-GIT	75	2	6
Souza et al 2014[344]	Brazil	31–100	Cross-sectional	Prospective	All	HIV-positive	Adults	71.7	QFT-GIT	300	1	6
Stefan et al 2010[345]	South Africa	201–	NS	NS	All	Cancer	Children	50	QFT-G	34	5	6
Stefan et al 2010[345]	South Africa	201–	NS	NS	All	Cancer	Children	50	T-SPOT	34	4	6
Stephan et al 2008[346]	Germany	0–30	Cross-sectional	Prospective	All	HIV-positive	Adults	81	QFT-G	275	1	6
Stephan et al 2008[346]	Germany	0–30	Cross-sectional	Prospective	All	HIV-positive	Adults	81	T-SPOT	275	8	6
Sultan et al 2013[347]	UK	0–30	Cohort	Prospective	All	HIV-positive	NS	78	QFT-GIT	117	1	4
Sultan et al 2013[347]	UK	0–30	Cohort	Prospective	All	HIV-positive	NS	78	T-SPOT	117	0	4
Sun et al 2015[348]	China, Taiwan	31–100	Cohort	Prospective	All	HIV-positive	Adults	81	T-SPOT	608	10	4
Sun et al 2015[348]	China, Taiwan	31–100	Cohort	Prospective	CD4: <200	HIV-positive	Adults	81	T-SPOT	104	1	4
Sun et al 2015[348]	China, Taiwan	31–100	Cohort	Prospective	CD4: ≥200	HIV-positive	Adults	81	T-SPOT	504	9	4
Takeda et al 2011[349]	Japan	0–30	NS	Prospective	All	IMID	Adults	25.8	QFT-G	350	39	6
Takeda et al 2011[349]	Japan	0–30	NS	Prospective	All	Healthy people	Adults	25.7	QFT-G	35	0	6
Talati et al 2009[350]	USA	0–30	Cross-sectional	NS	All	HIV-positive	Adults	35	QFT-GIT	336	6	6
Talati et al 2009[350]	USA	0–30	Cross-sectional	NS	All	HIV-positive	Adults	35	T-SPOT	336	47	6

Continue

	Area	TB burden	Study design	Prospective or retrospective	Subgroup	Population	Age group	Male (%)	Type of IGRA	Particip -ants (n)	Indeterm -inate (n)	Quadas score
Talebi-Taher et al 2011[351]	Iran	0–30	Cross-section al	NS	All	HCWs	Adults	36.5	QFT-GIT	200	2	6
Tanabe et al 2017[352]	Japan	0–30	NS	NS	All	HCWs	Adults	36.8	QFT-GIT	652	2	5
Tanabe et al 2017[352]	Japan	0–30	NS	NS	All	HCWs	Adults	36.8	T-SPOT	652	2	5
Tavast et al 2012[353]	Finland	0–30	NS	Retrospective	All	Immunocompromised patients	Adults	40	QFT-GIT	53	1	6
Tavast et al 2012[353]	Finland	0–30	NS	Retrospective	All	Immunocompromised patients	Adults	40	T-SPOT	82	2	6
Teranishi et al 2020[354]	Japan	0–30	Cohort	Retrospective	All	IMID	All age	51.3	T-SPOT	3837	104	5
Theel et al 2018[355]	USA	0–30	NS	Prospective	All	HCWs	Adults	48	QFT-GIT	97	0	6
Theodoropoulos et al 2012[356]	USA	0–30	Cohort	Retrospective	All	Transplant candidates	Adults	34	QFT-GIT	2392	206	5
Theodoropoulos et al 2012[356]	USA	0–30	Cohort	Retrospective	Immunocompromised	Transplant candidates	Adults	34	QFT-GIT	181	72	5
Theodoropoulos et al 2012[356]	USA	0–30	Cohort	Retrospective	Immunocompetent	Transplant candidates	Adults	34	QFT-GIT	66	6	5
Thi et al 2018[357]	UK	0–30	Cohort	Retrospective	All	IMID	Adults	51	IGRA	252	79	4
Thi et al 2018[357]	UK	0–30	Cohort	Retrospective	Immunocompromised	IMID	Adults	51	IGRA	181	72	4
Thi et al 2018[357]	UK	0–30	Cohort	Retrospective	Immunocompetent	IMID	Adults	51	IGRA	66	6	4
Thomas et al 2010[358]	USA	0–30	Cross-section al	Prospective	All	Children	Children	46.4	QFT-GIT	302	74	5
Thomaset al 2011[359]	UK	0–30	NS	NS	All	Contacts	Children	14.7	QFT-GIT	283	18	6
Thomaset al 2011[359]	UK	0–30	NS	NS	Age: 0-2y	Contacts	Children	14.7	QFT-GIT	69	13	6
Thomaset al 2011[359]	UK	0–30	NS	NS	Age: 2-15y	Contacts	Children	14.7	QFT-GIT	214	5	6
Tiernan et al 2013[360]	UK	0–30	Cohort	Prospective	All	IMID	All age	NS	QFT-GIT	204	1	4
Tiernan et al 2013[360]	UK	0–30	Cohort	Prospective	Immunocompromised	Immunocompromised patients	All age	NS	QFT-GIT	68	1	4
Tiernan et al 2013[360]	UK	0–30	Cohort	Prospective	Immunocompetent	Immunocompetent group	All age	NS	QFT-GIT	136	0	4
Tieu et al 2014[361]	Thailand	101–200	Cohort	Prospective	All	Contacts	Children	50.7	QFT-GIT	158	0	6
Triverio et al 2009[362]	Switzerland	0–30	NS	NS	All	Dialysis	NS	74.2	QFT-G	62	5	4
Triverio et al 2009[362]	Switzerland	0–30	NS	NS	All	Dialysis	NS	74.2	T-SPOT	62	7	4

Continue

	Area	TB burden	Study design	Prospective or retrospective	Subgroup	Population	Age group	Male (%)	Type of IGRA	Particip -ants (n)	Indeterm -inate (n)	Quadas score
Tsou et al 2015[363]	China, Taiwan	31–100	Cohort	Prospective	All	HCWs	Adults	100	QFT-G	147	8	5
Tsuyuzaki et al 2019[364]	Japan	31–100	Cross-sectional	Prospective	All	Contacts	All age	53.4	QFT-plus	412	0	6
Tsuyuzaki et al 2019[364]	Japan		Cross-sectional	Prospective	All	Contacts	All age	53.4	T-SPOT	412	0	6
Tuuminen et al 2012[365]	Finland	0–30	Cohort	Prospective	All	Contacts	Children	40	QFT-GIT	66	1	6
Umekita et al 2020[366]	Japan	0–30	Cohort	Retrospective	All	HIV-positive	Adults	20.7	T-SPOT	29	16	6
Umekita et al 2020[366]	Japan	0–30	Cohort	Retrospective	All	Healthy people	Adults	20.7	T-SPOT	87	1	6
Vajravelu et al 2017[367]	USA	0–30	Cohort	Retrospective	All	IMID	All age	46.67	QFT-GIT	411	80	5
Vassilopoulos et al 2011[368]	Greece	0–30	Cohort	Prospective	All	IMID	NS	42	QFT-GIT	157	2	6
Vassilopoulos et al 2011[368]	Greece	0–30	Cohort	Prospective	All	IMID	NS	42	T-SPOT	157	0	6
Velasco-Arnaiz et al 2018[369]	Spain	0–30	Cohort	Prospective	All	Young children	Young children	51.2	QFT-GIT	249	9	6
Velasco-Arnaiz et al 2020[370]	Australia	0–30	NS	Prospective	All	Young children	Young children	NS	QFT-GIT	112	3	4
Velasco-Arnaiz et al 2022[371]	Spain	0–30	Cohort	NS	All	Young children	Young children	51	QFT-GIT	204	14	6
Venkatappa et al 2019[372]	USA	0–30	NS	NS	All	High risk	All age	49	QFT-plus	520	2	6
Verhagen et al 2012[373]	Venezuela	31–100	Cross-sectional	NS	All	Children	Children	43.6	QFT-GIT	150	12	6
Verhagen et al 2014[374]	USA	0–30	Cohort	Prospective	All	Children	Children	54	QFT-GIT	142	10	5
Verhagen et al 2014[374]	USA	0–30	Cohort	Prospective	Age: 0-4y	Children	Children	54	QFT-GIT	39	5	5
Verhagen et al 2014[374]	USA	0–30	Cohort	Prospective	Age: 5-15y	Children	Children	54	QFT-GIT	112	6	5
Vortia et al 2018[375]	USA	0–30	Cohort	Prospective	All	IMID	Children	65	QFT-GIT	93	1	6
Wang et al 2020[376]	China, Taiwan	31–100	Cohort	Retrospective	All	IMID	Adults	69.3	QFT-GIT	238	14	6
Wassie et al 2013[377]	Ethiopia	101–200	Cross-sectional	NS	All	Healthy people	All age	54.7	QFT-GIT	245	7	6
Wei et al 2013[378]	China	31–100	NS	NS	All	HCWs	Adults	22.4	QFT-GIT	210	9	6
Weinberg et al 2021[379]	USA	0–30	RCT	Prospective	All	Pregnants	Adults	0	QFT-GIT	944	61	6
Weinfurter et al 2011[380]	USA	0–30	Cross-sectional	NS	All	High risk	Adults	73.1	QFT-GIT	1696	36	5
Wendorf et al 2020[381]	USA	0–30	Cohort	Retrospective	All	Children	Children	52	QFT-G	1878	22	5
Wendorf et al 2020[381]	USA	0–30	Cohort	Retrospective	Age: 0-2y	Children	Children	52	QFT-G	434	3	5
Wendorf et al 2020[381]	USA	0–30	Cohort	Retrospective	Age: 2-5y	Children	Children	52	QFT-G	1444	19	5
Wigg et al 2019[382]	Australia	0–30	Cohort	Retrospective	All	Transplant candidates	Adults	72.2	QFT-G	155	39	6
Wikell et al 2021[383]	Sweden	0–30	NS	NS	All	High risk	NS	NS	QFT-plus	58539	927	6

Continue

	Area	TB burden	Study design	Prospective or retrospective	Subgroup	Population	Age group	Male (%)	Type of IGRA	Particip-ants (n)	Indeterm-inate (n)	Quadas score
Wilder-Smith et al 2005[384]	Singapore	31–100	NS	Prospective	All	Healthy people	Adults	38	QFT-GIT	357	7	4
Winthrop et al 2008[385]	USA	0–30	Cohort	Prospective	All	Dialysis	Adults	53	QFT-G	124	2	6
Winthrop et al 2008[385]	USA	0–30	Cohort	Prospective	All	Dialysis	Adults	53	T-SPOT	124	3	6
Wong et al 2014[386]	China, Hong Kong	31–100	Cohort	Prospective	All	IMID	Adults	55	QFT-G	265	4	6
Wong et al 2014[386]	China, Hong Kong	31–100	Cohort	Prospective	Immunocompromised	IMID	Adults	55	QFT-G	123	3	6
Wong et al 2014[386]	China, Hong Kong	31–100	Cohort	Prospective	Immunocompetent	IMID	Adults	55	QFT-G	142	1	6
Wong et al 2014[386]	China, Hong Kong	31–100	Cohort	Prospective	Healthy control	Healthy people	Adults	55	QFT-G	234	0	6
Wu et al 2019[387]	China, Taiwan	31–100	Cohort	Retrospective	All	IMID	Adults	74	QFT-GIT	100	1	6
Xu et al 2022[388]	China	31–100	NS	Prospective	All	Immunocompromised patients	Adults	56.8	QFT-plus	278	31	5
Xu et al 2022[388]	China	31–100	NS	Prospective	All	Healthy people	Adults	56.8	QFT-plus	175	15	5
Yang et al 2013[389]	Taiwan, China	31–100	Cohort	Prospective	All	HIV-positive	Adults	97.6	T-SPOT	1001	18	6
Yap et al 2018[390]	Singapore	31–100	Cross-sectional	NS	All	Healthy people	Adults	44.3	QFT-GIT	1682	8	5
Yassin et al 2011[391]	Ethiopia	101–200	Cross-sectional	NS	All	Contacts	Children	50.3	QFT-GIT	335	52	5
Yassin et al 2011[391]	Ethiopia	101–200	Cross-sectional	NS	All	Healthy people	Children	50.3	QFT-GIT	156	25	5
Yassin et al 2013[392]	Ethiopia	101–200	Cross-sectional	Prospective	All	HIV-positive	Children	50.7	QFT-GIT	398	73	6
Yassin et al 2013[392]	Ethiopia	101–200	Cross-sectional	Prospective	HIV-positive	HIV-positive	Children	50.7	QFT-GIT	27	4	6
Yassin et al 2013[392]	Ethiopia	101–200	Cross-sectional	Prospective	HIV-negative	HIV-negaitive	Children	50.7	QFT-GIT	374	69	6
Yilmaz et al 2012[393]	Turkey	0–30	NS	NS	All	IMID	All age	9	QFT-G	78	2	4
Yilmaz et al 2012[393]	Turkey	0–30	NS	NS	Immunocompromised	IMID	All age	9	QFT-G	37	0	4
Yilmaz et al 2012[393]	Turkey	0–30	NS	NS	Immunocompetent	IMID	All age	9	QFT-G	38	2	4
Yu et al 2015[394]	China	31–100	NS	NS	All	HIV-positive	Adults	87	QFT-GIT	106	5	6
Yu et al 2015[394]	China	31–100	NS	NS	CD4: <200	HIV-positive	Adults	87	QFT-GIT	29	2	6
Yu et al 2015[394]	China	31–100	NS	NS	CD4: 200-500	HIV-positive	Adults	87	QFT-GIT	63	1	6
Yu et al 2015[394]	China	31–100	NS	NS	CD4: >500	HIV-positive	Adults	87	QFT-GIT	14	2	6
Yun et al 2014[395]	South Korea	31–100	NS	Retrospective	All	Immunocompromised patients	Adults	NS	QFT-GIT	683	78	6

Continue

	Area	TB burden	Study design	Prospective or retrospective	Subgroup	Population	Age group	Male (%)	Type of IGRA	Particip-ants (n)	Indeterm-inate (n)	Quadas score
Yun et al 2016[396]	South Korea	31–100	NS	NS	All	Children	Children	NS	QFT-GIT	106	3	6
Zellweger et al 2015[397]	European countries	0–30	Cohort	Prospective	All	Contacts	All age	45.8	QFT-GIT	5020	27	5
Zellweger et al 2015[397]	European countries	0–30	Cohort	Prospective	Adults	Contacts	All age	45.8	QFT-GIT	4526	26	5
Zellweger et al 2015[397]	European countries	0–30	Cohort	Prospective	Children	Contacts	All age	45.8	QFT-GIT	495	1	5
Zhang et al 2017[398]	China	31–100	Cohort	Prospective	Healthy control	Healthy people	Adults	44.97	QFT-GIT	17796	532	4
Zhang et al 2019[399]	China	31–100	Cohort	Prospective	All	HCWs	Adults	68.34	QFT-GIT	625	0	6
Zhang et al 2019[399]	China	31–100	Cohort	Prospective	All	HCWs	Adults	68.34	QFT-plus	625	0	6
Zhang et al 2019[399]	China	31–100	Cohort	Prospective	All	HCWs	Adults	68.34	T-SPOT	625	0	6
Zhang et al 2022[400]	China	31–100	Cohort	Prospective	All	IMID	Children	85.4	QFT-GIT	108	13	6
Zhang et al 2022[400]	China	31–100	Cohort	Prospective	All	IMID	Children	85.4	T-SPOT	108	6	6
Zhao et al 2011[401]	China	31–100	Cross-sectional	NS	All	Healthy people	Adults	48.8	T-SPOT	898	47	4
Zrinski Topić et al 2011[402]	Croatia	0–30	NS	Retrospective	All	Children	Children	51	IGRA	2173	10	6
Zrinski Topić et al 2011[402]	Croatia	0–30	NS	Retrospective	Age: 0-5y	Children	Children	51	IGRA	313	2	6
Zrinski Topić et al 2011[402]	Croatia	0–30	NS	Retrospective	Age: 5-15y	Children	Children	51	IGRA	1860	8	6
Zwerling et al 2014[403]	India	101–200	NS	NS	All	HCWs	Children	8	QFT-GIT	280	1	3

Healthy people included general population and healthy controls for high-risk population in the included study. Abbreviations: HCWs, Health Care Workers; IMID, immune-mediated inflammatory diseases; IGRA, interferon-γ release assays; TST, tuberculin skin tests; QFT, QuantiFERON®-TB; T-SPOT, T-SPOT; OR, odds ratio; RD, risk difference; NS, not specified; RCT, randomized controlled trial.

Table S5. Summary of study quality (items from the modified QUADAS-2).

	Yes n (%)	No n (%)	Unclear n (%)
Participants select bias			
Was a consecutive or random sample of participants enrolled?	372 (92.3)	9 (2.2)	22 (5.5)
Did the study have appropriate exclusions?	365 (90.6)	23 (5.7)	15 (3.7)
Test conduct bias			
Was how the tests were conducted and interpreted adequately described?	377 (93.5)	17 (4.2)	9 (2.2)
Was the definition of indeterminate for IGRA adequately described?	338 (83.9)	35 (8.7)	30 (7.4)
Flow and outcome			
Were all participants included in the analysis?	370 (91.8)	31 (7.7)	2 (0.5)
Was the reason for the participant's withdrawal from the analysis explained?	348 (86.4)	55 (13.6)	0 (0)

Abbreviations: LTBI, latent tuberculosis infection.

Table S6. Meta-regression analysis for the indeterminate rate.

	Univariate analysis			Multivariate analysis	
	*p*val	Estimat (95% CI)	R^2 (%)	*P* value	Estimat (95% CI)
Study design			0.00		
Cohort	ref	ref		ref	ref
Cross-sectional	0.0937	-0.0177 (-0.0383 to 0.0030)		0.8520	-0.0031 (-0.0277 to 0.0215)
RCT	0.1036	-0.0649 (-0.1430 to 0.0132)		0.7503	0.0178 (-0.0759 to 0.1115)
NS	0.0711	0.0210 (-0.0018 to 0.0438)		0.0537	0.0249 (-0.0016 to 0.0514)
Timing of data collection			0.00		
NS	ref	ref		ref	ref
Prospective	0.2143	-0.0124 (-0.0320 to 0.0072)		0.1345	-0.0151 (-0.0389 to 0.0087)
Retrospective	** 0.0054	0.0335 (0.0099 to 0.0570)		* 0.0192	0.0420 (0.0124 to 0.0716)
Quality			1.84		
High	ref	ref		ref	ref
Moderate	0.3749	0.0121 (-0.0146 to 0.0387)		0.8201	0.0028 (-0.0250 to 0.0307)
Participants			11.52		
101-200	ref	ref		ref	ref
15-100	0.2140	0.0131 (-0.0076 to 0.0338)		** 0.0069	0.0414 (0.0110 to 0.0709)
201-500	0.8818	-0.0027 (-0.0387 to 0.0332)		0.3665	0.0142 (-0.0107 to 0.0391)
More than 500	0.7721	0.0062 (-0.0357 to 0.0481)		** 0.0026	-0.0402 (-0.0674 to -0.0129)
TB burden			0.00		
0–30 per 100, 000	ref	ref		ref	ref
31–100 per 100, 000	0.2140	0.0131 (-0.0076 to 0.0338)		0.2799	0.0133 (-0.0093 to 0.0359)
101–200 per 100, 000	0.8818	-0.0027 (-0.0387 to 0.0332)		0.7581	0.0018 (-0.0378 to 0.0414)
≥201 per 100, 000	0.7721	0.0062 (-0.0357 to 0.0481)		0.7257	0.0227 (-0.0243 to 0.0698)

Continue

	Univariate analysis			Multivariate analysis	
	*p*val	Estimat (95% CI)	R^2 (%)	*P* value	Estimat (95% CI)
Population			0.00		
Contacts	ref	ref		ref	ref
Healthy controls	0.6767	-0.0084 (-0.0477 to 0.0310)		0.5081	-0.0069 (-0.0605 to 0.0467)
High risk	0.8512	-0.0065 (-0.0750 to 0.0619)		0.3159	0.0382(-0.0315 to 0.1080)
Immunosuppressed patients	*** <0.0001	0.0954 (0.0649 to 0.1260)		*** <0.0002	0.0751 (0.0382 to 0.1120)
With possibility of contact	0.5885	-0.0100 (-0.0461 to 0.0262)		0.3009	-0.0119 (-0.0548to 0.0310)
With possibility of immunosuppression	*** <0.0001	0.0759 (0.0460 to 0.1059)		** 0.0036	0.0519 (0.0188 to 0.0849)
Age			0.00		
Adults	ref	ref		ref	ref
All age	*** <0.0001	-0.0668 (-0.0917 to -0.0419)		** 0.0040	-0.0400 (-0.0680 to -0.0120)
Children	0.2896	-0.0128 (-0.0365 to 0.0109)		0.5301	-0.0069 (-0.0357 to 0.02418)
NS	0.1052	-0.0331 (-0.0732 to 0.0069)		0.3334	-0.0225 (-0.0640 to 0.0190)
Men (%)			0.00		
0–25	ref	ref		ref	ref
25.1–50	0.2055	-0.0209 (-0.0531 to 0.0114)		0.8068	0.0042 (-0.0297 to 0.0380)
50.1–75	0.7376	0.0054 (-0.0262 to 0.0370)		0.7439	0.0030 (-0.0307 to 0.0368)
75.1–100	0.1345	-0.0306 (-0.0706 to 0.0095)		** 0.0080	-0.0518 (-0.0941 to -0.0095)
NS	0.7467	-0.0069 (-0.0491 to 0.0352)		0.9150	0.0126 (-0.0314 to 0.0566)
IGRA type			0.00		
IGRA (combine)	ref	ref		ref	ref
QFT	0.3064	0.1168 (-0.1070 to 0.3405)		0.4181	0.0900 (-0.1279 to 0.3079)
QFT-G	0.8203	0.0081 (-0.0473 to 0.0635)		0.9112	-0.0049 (-0.0489 to 0.0587)
QFT-GIT	0.0964	-0.0421 (-0.0917 to 0.0075)		0.4690	-0.0225 (-0.0707 to 0.0257)
QFT-plus	** 0.0011	-0.1009 (-0.1776 to -0.0442)		* 0.0265	-0.0783 (-0.1440 to -0.0125)
T-SPOT	0.4874	0.0210 (-0.0384 to 0.0805)		0.1264	0.0427 (-0.0158 to 0.1013)
Area			0.00		
Africa	ref	ref		ref	ref
Asia	0.7505	-0.0062 (-0.0441 to 0.0318)		0.1664	-0.0400 (-0.0967 to 0.0166)
Europe	0.2911	-0.0206 (-0.0587 to 0.0176)		0.1778	-0.0433 (-0.1063 to 0.0197)
Oceania	0.0882	-0.0182 (-0.0587 to 0.0222)		0.1954	-0.0780 (-0.1526 to -0.0034)
North America	0.3775	0.0711 (-0.0106 to 0.1529)		0.2652	-0.0365 (-0.1008 to 0.0277)
South America	0.4726	-0.0213 (-0.0793 to 0.0368)		* 0.0404	-0.0780 (-0.1526 to -0.0034)

Abbreviations: IGRA, interferon-γ release assays; QFT, QuantiFERON®-TB; QFT-G, QuantiFERON®-TB Gold, the second generation of QFT; QFT-GIT, QuantiFERON®-TB Gold In Tube, the third generation of QFT; QFT-plus, QuantiFERON®-TB PLUS, the forth generation of QFT; T-SPOT, T-SPOT.TB.

Table S7. Subgroup analysis of the indeterminate rate.

	Studies (n)	Participants (n)	Indeterminate events (%)	I^2 (%)
Type of study				
Cohort	189	166,196	3.9 (3.4–4.4)	96
Cross-sectional	135	146,076	3.3 (2.7–3.9)	97
RCTs	5	5951	1.8 (0.2–4.9)	98
NS	103	168,663	4.8 (4.0–5.6)	98
Timing of data collection				
Prospective	185	141,412	3.3 (2.9–3.8)	95
Retrospective	89	157,634	5.2 (4.4–6.0)	98
NS	158	187,840	3.8 (3.2–4.4)	97
TB burden of the areas				
0–30	276	349,370	3.7 (3.3–4.1)	97
31–100	107	110,576	4.3 (3.6–5.1)	97
101–200	28	6884	3.6 (1.9–5.8)	95
More than 200	21	20,056	4.1 (2.4–6.2)	97
Age group				
Adults	257	168,970	4.4 (4.0–5.0)	95
Children	85	48,408	4.1 (3.2–5.1)	96
All age	67	191,776	2.1 (1.6–2.7)	98
NS	23	77,732	3.2 (2.1–4.4)	97
Proportion of men				
0–25	45	38,659	4.4 (2.9–6.0)	97
25.1–50	169	212,194	3.4 (2.9–3.9)	97
50.1–75	138	102,607	4.4 (3.8–5.1)	96
75.1–100	44	54,619	2.9 (2.2–3.7)	92
NS	36	78,807	3.9 (2.8–5.2)	97
Number of participants				
15–100	91	5886	5.4 (4.1–6.8)	82
101–200	120	16,596	4.2 (3.4–5.2)	88
201–500	112	33,905	4.6 (3.7–5.7)	95
More than 501	109	430,499	2.3 (1.9–2.7)	99
Type of IGRA				
QFT	1	60	11.7 (4.9–20.9)	-
QFT-G	55	53,161	5.5 (4.4–6.7)	95
QFT-GIT	283	311,766	3.6 (3.2–4.0)	97
QFT-plus	17	65,623	1.5 (0.5–2.9)	97
T-SPOT	31	14,997	6.3 (4.7–8.1)	94
IGRA	16	11,203	5.6 (2.9–9.0)	98
Quality				
Middle	53	41,482	4.3 (3.3–5.5)	96
High	350	415,328	3.8 (3.5–4.2)	97

Continue

	Studies (n)	Participants (n)	Indeterminate events (%)	I^2 (%)
Area				
Asia	142	123,354	4.2 (3.5–4.9)	97
Africa	30	22,963	4.5 (2.9–6.3)	97
Europe	133	123,9264	3.6 (3.0–4.2)	96
Oceania	6	3099	3.7 (1.6–6.6)	92
North America	75	165,250	3.7 (3.0–4.4)	98
South America	17	2880	3.7 (1.6–6.6)	92

*Healthy people included general population and healthy controls for high-risk population in the included study. Abbreviations: IGRA, interferon-γ release assays; QFT, QuantiFERON®-TB; QFT-G, QuantiFERON®-TB Gold, the second generation of QFT; QFT-GIT, QuantiFERON®-TB Gold In Tube, the third generation; QFT-plus, QuantiFERON®-TB PLUS, the forth generation; T-SPOT, T-SPOT.TB.

Table S8.Comparison of IGRA indeterminate rates between QFT and T-SPOT in head-to-head studies.

	Studies (n)	Participants (n)	Indeterminate rate (%)	I^2 (%)	OR	I^2 (%)
All QFT vs. T-SPOT						
All QFT	55	50,330	2.4 (1.8–3.1)	93	0.88 (0.59–1.32)	91
T-SPOT	55	49,575	2.8 (1.5–4.4)	99		
QFT-G vs. T-SPOT						
QFT-G	8	913	4.9 (1.7–9.7)	86	1.68 (0.76–3.70)	39
T-SPOT	8	925	2.4 (0.9–4.5)	66		
QFT-GIT vs. T-SPOT						
QFT-GIT	41	46,458	2.6 (1.9–3.4)	94	0.86 (0.54–1.37)	93
T-SPOT	41	46,615	3.2 (1.6–5.3)	99		
QFT-plus vs. T-SPOT						
QFT-plus	6	2959	0.3 (0.0–1.4)	91	0.24 (0.16–0.35)	0
T-SPOT	6	3035	1.0 (0.0–5.6)	98		

Table S9. Comparison of the indeterminate rates among three generation of QFT.

	Studies (n)	Participants (n)	Indeterminate rate (%)	I^2 (%)	OR	I^2 (%)
QFT-GIT VS QFT-G						
QFT-GIT	4	1081	3.9 (0.6–10.0)	90	0.64 (0.23–1.75)	62
QFT-G	4	1081	5.3 (1.4–11.3)	87		
QFT-GIT VS QFT-plus						
QFT-GIT	4	2523	1.9 (0.1–5.8)	95	1.49 (0.98–2.17)	0
QFT-plus	4	2452	1.3 (0.1–4.1)	93		

Abbreviations: QFT, QuantiFERON®-TB; QFT-G, QuantiFERON®-TB Gold, the second generation of QFT; QFT-GIT, QuantiFERON®-TB Gold In Tube, the third generation of QFT; QFT-plus, QuantiFERON®-TB PLUS, the forth generation of QFT; T-SPOT, T-SPOT.TB.

Table S10. Comparison of IGRA indeterminate rates among different immune status.

	Studies (n)	Participants (n)	Indeterminate rate (%)	I^2 (%)	OR	I^2 (%)
Immunocompromised patients vs. controls						
Immunocompromised patients	16	1721	14.4 (7.0–23.8)	96	3.51 (2.11–5.82)	61
Controls	16	5946	4.6 (2.1–7.9)	95		
HIV-positive patients						
CD4+ cell less than 100 cells/mm^3	9	375	17.1 (9.0–27.1)	80	5.22 (2.66–10.25)	52
CD4+ cell more than 100 cells/mm^3	9	2483	3.3 (2.0–4.9)	71		
HIV-positive patients						
CD4+ cell less than 200 cells/mm^3	18	901	16.5 (10.0–24.1)	87	5.26 (3.04–9.10)	58
CD4+ cell more than 200 cells/mm^3	18	3610	2.8 (1.5–4.6)	85		
HIV-positive patients						
CD4+ cell less than 350 cells/mm^3	8	942	9.1 (6.0–12.7)	64	3.36 (1.95–5.8)	31
CD4+ cell more than 350 cells/mm^3	8	1711	2.3 (1.5–3.3)	25		
HIV-positive patients						
CD4+ cell less than 500 cells/mm^3	5	833	6.4 (3.1–10.9)	79	2.23 (0.44–11.36)	62
CD4+ cell more than 500 cells/mm^3	5	411	0.7 (0.0–3.4)	68		

Table S11. Comparison of the indeterminate rates in HIV-positive patients stratified by CD4+ cell count.

	Studies (n)	Participants (n)	Indeterminate events (%)	I^2 (%)
CD4+ cell count threshold: 100 and 200				
CD4+ cell less than 100 cells/mm^3	7	243	15.5 (9.5–22.7)	40
CD4+ cell 100-200 cells/mm^3	7	230	8.7 (4.2–14.5)	47
CD4+ cell more than 200 cells/mm^3	7	1589	2.1 (0.4–5.0)	89
CD4+ cell count threshold: 200 and 350				
CD4+ cell less than 200 cells/mm^3	7	320	15.8 (9.7–23.0)	54
CD4+ cell 200-350 cells/mm^3	7	480	4.8 (2.7–7.4)	21
CD4+ cell more than 350 cells/mm^3	7	1559	2.1 (1.3–3.1)	24

Table S12. Comparison of IGRA indeterminate rates among different age group.

	Studies (n)	Participants (n)	Indeterminate rate (%)	I^2 (%)	OR	I^2 (%)
Children vs. adults						
Children	7	2052	3.6 (0.4–9.9)	96	2.53 (1.78–3.60)	41
Adults	7	6404	1.5 (0.4–3.5)	92		
0–2 years vs. 2–15 years						
0–2 years	6	615	7.9 (3.2–14.5)	85	2.88 (1.70–4.87)	44
2–15 years	6	6684	2.9 (1.2–5.4)	95		
0–5 years vs. 5–15 years						
0–5 years	8	2394	7.8 (3.2–14.2)	96	2.03 (1.57–2.61)	0
5–15 years	8	7183	4.0 (1.7–7.3)	97		
0–2 years vs. 2–5 years						
0–2 years	6	980	4.8 (1.5–9.7)	87	1.26 (0.64–2.49)	55
2–5 years	6	2839	3.6 (1.8–6.0)	85		

Table S13. Comparison of indeterminate rates of IGRA caused by failed positive control and failed negative control among different population.

	Low mitogen response				High nil response			
	Studies (n)	Indeterminate (n)	Proportion (%)	I^2 (%)	Studies (n)	Indeterminate (n)	Proportion (%)	I^2 (%)
All populations	71	2599	94.6 (89.6–98.0)	95	66	2317	4.0 (1.4–12.4)	93
Immunocompetent population	13	430	99.4 (97.3–100.0)	51	13	430	1.0 (0.0–3.5)	52
Healthy people*	2	66	96.6 (65.4–100.0)	74	2	48	5.5 (0.0–23.6)	42
Recent contacts	6	56	98.5 (86.7–100.0)	66	6	56	1.5 (0.0–13.3)	66
HCWs	4	200	99.6 (98.2–100.0)	0	4	200	0.4 (0.0–1.8)	0
Immigrants or refugees	1	126	100.0 (99.2–10.00)	-	1	126	0.0 (0.0–0.8)	-
Immunocompromised patients (all)	25	588	95.7 (85.6–99.9)	95	23	343	2.6 (0.2–7.2)	72
Immunocompromised patients*	4	66	100.0 (98.6–100.0)	0	4	66	0.0 (0.0–1.4)	0
HIV-positive	14	184	95.2 (85.0–99.8)	78	14	184	4.8 (0.2–15.0)	78
Hemodialysis	2	49	89.1 (74.1–98.1)	49	2	49	10.9 (1.9–25.9)	99
Transplant recipients	4	259	90.7 (46.9–100.0)	97	2	14	0.0 (0.0–6.7)	0
Cancer	1	30	100.0 (96.8–100.0)	-	1	30	0.0(0.0–3.2)	-
With possibility of immunosuppression	30	939	91.7 (83.2–97.5)	93	27	902	5.6 (1.4–12.4)	90
IMID	14	254	94.4 (79.8–100.0)	90	14	265	5.4 (0.2–17.3)	87
Prisoners	1	160	96.9 (93.6–99.0)	-	1	160	7.5 (3.9–12.1)	-
Children	10	410	86.8 (71.4–96.8)	0	7	362	4.4 (0.0–15.4)	90
Young children	3	66	100.0 (98.6–100.0)	0	3	32	18.9 (0.0–98.0)	96
Pregnant women	1	61	100.0 (98.4–100.0)	-	1	61	0.0 (0.0–1.6)	-
Diabetes	1	22	100.0 (95.7–100.0)	-	1	22	0.0 (0.0–4.3)	-
High risk (combined)	3	642	72.5 (24.0–99.8)	99	3	642	27.5 (0.2–76.0)	99

*The target population of these studies was a mixed group of immunocompromised individuals, including HIV-positive patients, cancer, hemodialysis, and/or transplant recipients. Abbreviations: HCWs, health care workers; IMID, immune-mediated inflammatory diseases; IGRA, interferon-γ release assays.

References

1 Abdelwahab HW, Elmaria MO, Abdelghany DA, et al. Screening of latent TB infection in patients with recently diagnosed bronchogenic carcinoma. *Asian Cardiovasc Thorac Ann* 2021; **29**: 208-13.

2 Abdulkareem FN, Merza MA, Salih AM. First insight into latent tuberculosis infection among household contacts of tuberculosis patients in Duhok, Iraqi Kurdistan: using tuberculin skin test and QuantiFERON-TB Gold Plus test. *Int J Infect Dis* 2020; **96**: 97-104.

3 Abreu C, Almeida F, Ferraz R, et al. The tuberculin skin test still matters for the screening of latent Tuberculosis infections among Inflammatory Bowel Disease patients. *Dig Liver Dis* 2016; **48**: 1438-43.

4 Abubakar I, Drobniewski F, Southern J, et al. Prognostic value of interferon-γ release assays and tuberculin skin test in predicting the development of active tuberculosis (UK PREDICT TB): a prospective cohort study. *Lancet Infect Dis* 2018; **18**: 1077-87.

5 Adetifa IM, Ota MO, Jeffries DJ, et al. Commercial interferon gamma release assays compared to the tuberculin skin test for diagnosis of latent Mycobacterium tuberculosis infection in childhood contacts in the Gambia. *Pediatr Infect Dis J* 2010; **29**: 439-43.

6 Agarwal S, Nguyen DT, Lew JD, et al. Comparing TSPOT assay results between an Elispot reader and manual counts. *Tuberculosis (Edinb)* 2016; **101s**: S92-92s98.

7 Ahmadinejad Z, Azmoudeh Ardalan F, Safy S, et al. Diagnosis of latent tuberculosis infection in candidates for kidney transplantation (comparison of two tests). *Acta Med Iran* 2012; **50**: 305-10.

8 Ahmadinejad Z, Azmoudeh Ardalan F, Razzaqi M, et al. QuantiFERON-TB Gold In-Tube test for diagnosis of latent tuberculosis (TB) infection in solid organ transplant candidates: a single-center study in an area endemic for TB. *Transpl Infect Dis* 2013; **15**: 90-95.

9 Ahmed A, Feng PI, Gaensbauer JT, et al. Interferon-γ Release Assays in Children <15 Years of Age. *Pediatrics* 2020; **145** .

10 Aichelburg MC, Rieger A, Breitenecker F, et al. Detection and prediction of active tuberculosis disease by a whole-blood interferon-gamma release assay in HIV-1-infected individuals. *Clin Infect Dis* 2009; **48**: 954-62.

11 Aichelburg MC, Reiberger T, Breitenecker F, et al. Reversion and conversion of interferon-γ release assay results in HIV-1-infected individuals. *J Infect Dis* 2014; **209**: 729-33.

12 Aichelburg MC, Mandorfer M, Tittes J, et al. The association of smoking with IGRA and TST results in HIV-1-infected subjects. *Int J Tuberc Lung Dis* 2014; **18**: 709-16.

13 Al Hajoj S, Varghese B, Datijan A, et al. Interferon Gamma Release Assay versus Tuberculin Skin Testing among Healthcare Workers of Highly Diverse Origin in a Moderate Tuberculosis Burden Country. *PLoS One* 2016; **11**: e0154803.

14 Al Mekaini LA, Al Jabri ON, Narchi H, et al. The use of an interferon-gamma release assay to screen for pediatric latent tuberculosis infection

in the eastern region of the Emirate of Abu Dhabi. *Int J Infect Dis* 2014; **23**: 4-7.

15 Al-Taweel T, Strohl M, Pai M, et al. A Study of Optimal Screening for Latent Tuberculosis in Patients with Inflammatory Bowel Disease. *Dig Dis Sci* 2018; **63**: 2695-702.

16 Amorim RF, Viegas ERC, Carneiro AJV, et al. Superiority of Interferon Gamma Assay Over Tuberculin Skin Test for Latent Tuberculosis in Inflammatory Bowel Disease Patients in Brazil. *Dig Dis Sci* 2019; **64**: 1916-22.

17 Andrews JR, Hatherill M, Mahomed H, et al. The dynamics of QuantiFERON-TB gold in-tube conversion and reversion in a cohort of South African adolescents. *Am J Respir Crit Care Med* 2015; **191**: 584-91.

18 Andrews JR, Nemes E, Tameris M, et al. Serial QuantiFERON testing and tuberculosis disease risk among young children: an observational cohort study. *Lancet Respir Med* 2017; **5**: 282-90.

19 Andrisani G, Armuzzi A, Papa A, et al. Comparison of Quantiferon-TB Gold versus tuberculin skin test for tuberculosis screening in inflammatory bowel disease patients. *J Gastrointestin Liver Dis* 2013; **22**: 21-25.

20 Arenas Miras Mdel M, Hidalgo-Tenorio C, Jimenez-Gamiz P, et al. Diagnosis of latent tuberculosis in patients with systemic lupus erythematosus: T.SPOT.TB versus tuberculin skin test. *Biomed Res Int* 2014; **2014**: 291031.

21 Arias-Guillén M, Riestra S, de Francisco R, et al. T-cell profiling and the immunodiagnosis of latent tuberculosis infection in patients with inflammatory bowel disease. *Inflamm Bowel Dis* 2014; **20**: 329-38.

22 Arias-Guillén M, Sánchez Menéndez MM, Alperi M, et al. High rates of tuberculin skin test positivity due to methotrexate therapy: False positive results. *Semin Arthritis Rheum* 2018; **48**: 538-46.

23 Atikan BY, Cavusoglu C, Dortkardesler M, et al. Assessment of tuberculosis infection during treatment with biologic agents in a BCG-vaccinated pediatric population. *Clin Rheumatol* 2016; **35**: 427-31.

24 Balcells ME, Pérez CM, Chanqueo L, et al. A comparative study of two different methods for the detection of latent tuberculosis in HIV-positive individuals in Chile. *Int J Infect Dis* 2008; **12**: 645-52.

25 Baldassari LE, Feng J, Macaron G, et al. Tuberculosis screening in multiple sclerosis: effect of disease-modifying therapies and lymphopenia on the prevalence of indeterminate TB screening results in the clinical setting. *Mult Scler J Exp Transl Clin* 2019; **5**: 2055217319875467.

26 Balkhy HH, El Beltagy K, El-Saed A, et al. Prevalence of Latent Mycobacterium Tuberculosis Infection (LTBI) in Saudi Arabia; Population based survey. *Int J Infect Dis* 2017; **60**: 11-16.

27 Banach DB, Harris TG. Indeterminate QuantiFERON®-TB Gold results in a public health clinic setting. *Int J Tuberc Lung Dis* 2011; **15**: 1623-30.

28 Bandiara R, Indrasari A, Dewi Rengganis A, et al. Risk factors of latent tuberculosis among chronic kidney disease with routine haemodialysis patients. *J Clin Tuberc Other Mycobact Dis* 2022; **27**: 100302.

29 Banfield S, Pascoe E, Thambiran A, et al. Factors associated with the performance of a blood-based interferon-γ release assay in diagnosing tuberculosis. *PLoS One* 2012; **7**: e38556.

30 Bartalesi F, Vicidomini S, Goletti D, et al. QuantiFERON-TB Gold and the TST are both useful for latent tuberculosis infection screening in autoimmune diseases. *Eur Respir J* 2009; **33**: 586-93.

31 Bartalesi F, Goletti D, Spinicci M, et al. Serial QuantiFERON TB-gold in-tube testing during LTBI therapy in candidates for TNFi treatment. *J Infect* 2013; **66**: 346-56.

32 Basu Roy R, Sotgiu G, Altet-Gómez N, et al. Identifying predictors of interferon-γ release assay results in pediatric latent tuberculosis: a protective role of bacillus Calmette-Guerin?: a pTB-NET collaborative study. *Am J Respir Crit Care Med* 2012; **186**: 378-84.

33 Behar SM, Shin DS, Maier A, et al. Use of the T-SPOT.TB assay to detect latent tuberculosis infection among rheumatic disease patients on immunosuppressive therapy. *J Rheumatol* 2009; **36**: 546-51.

34 Bélard E, Semb S, Ruhwald M, et al. Prednisolone treatment affects the performance of the QuantiFERON gold in-tube test and the tuberculin skin test in patients with autoimmune disorders screened for latent tuberculosis infection. *Inflamm Bowel Dis* 2011; **17**: 2340-49.

35 Bergot E, Haustraete E, Malbruny B, et al. Observational study of QuantiFERON®-TB gold in-tube assay in tuberculosis contacts in a low incidence area. *PLoS One* 2012; **7**: e43520.

36 Beshir MR, Zidan AE, El-Saadny HF, et al. Evaluation of the Immune Response to Interferon Gamma Release Assay and Tuberculin Skin Test Among BCG Vaccinated Children in East of Egypt: A Cross-Sectional Study. *Medicine (Baltimore)* 2016; **95**: e3470.

37 Bianchi L, Galli L, Moriondo M, et al. Interferon-gamma release assay improves the diagnosis of tuberculosis in children. *Pediatr Infect Dis J* 2009; **28**: 510-14.

38 Birku M, Desalegn G, Kassa G, et al. Effect of pregnancy and HIV infection on detection of latent TB infection by Tuberculin Skin Test and QuantiFERON-TB Gold In-Tube assay among women living in a high TB and HIV burden setting. *Int J Infect Dis* 2020; **101**: 235-42.

39 Bocchino M, Matarese A, Bellofiore B, et al. Performance of two commercial blood IFN-gamma release assays for the detection of Mycobacterium tuberculosis infection in patient candidates for anti-TNF-alpha treatment. *Eur J Clin Microbiol Infect Dis* 2008; **27**: 907-13.

40 Borgia P, Cambieri A, Chini F, et al. Suspected transmission of tuberculosis in a maternity ward from a smear-positive nurse: preliminary results of clinical evaluations and testing of neonates potentially exposed, Rome, Italy, 1 January to 28 July 2011. *Euro Surveill* 2011; **16:** 19984.

41 Bouley AJ, Baber U, Egnor E, et al. Prevalence of Latent Tuberculosis in the Multiple Sclerosis Clinic and Effect of Multiple Sclerosis Treatment on Tuberculosis Testing. *Int J MS Care* 2021; **23**: 26-30.

42 Bourgarit A, Baron G, Breton G, et al. Latent Tuberculosis Infection Screening and 2-Year Outcome in Antiretroviral-Naive HIV-Infected Patients in a Low-Prevalence Country. *Ann Am Thorac Soc* 2015; **12**: 1138-45.

43 Bradshaw L, Davies E, Devine M, et al. The role of the interferon gamma release assay in assessing recent tuberculosis transmission in a hospital incident. *PLoS One* 2011; **6**: e20770.

44 Brock I, Ruhwald M, Lundgren B, et al. Latent tuberculosis in HIV positive, diagnosed by the M. tuberculosis specific interferon-gamma test. *Respir Res* 2006; **7**: 56.

45 Brown J, Kumar K, Reading J, et al. Frequency and significance of indeterminate and borderline Quantiferon Gold TB IGRA results. *Eur*

Respir J 2017; **50**: 1701267.

46 Bua A, Molicotti P, Ruggeri M, et al. Interferon-γ release assay in people infected with immunodeficiency virus. *Clin Microbiol Infect* 2011; **17**: 402-04.

47 Buonsenso D, Delogu G, Perricone C, et al. Accuracy of QuantiFERON-TB Gold Plus Test for Diagnosis of Mycobacterium tuberculosis Infection in Children. *J Clin Microbiol* 2020; **58**:e00272-20.

48 Cabriada JL, Ruiz-Zorrilla R, Barrio J, et al. Screening for latent tuberculosis infection in patients with inflammatory bowel disease: Can interferon-gamma release assays replace the tuberculin skin test. *Turk J Gastroenterol* 2018; **29**: 292-98.

49 Calabrese C, Overman RA, Dusetzina SB, et al. Evaluating Indeterminate Interferon-γ-Release Assay Results in Patients With Chronic Inflammatory Diseases Receiving Immunosuppressive Therapy. *Arthritis Care Res (Hoboken)* 2015; **67**: 1063-69.

50 Calzada-Hernández J, Anton-López J, Bou-Torrent R, et al. Tuberculosis in pediatric patients treated with anti-TNFα drugs: a cohort study. *Pediatr Rheumatol Online J* 2015; **13**: 54.

51 Calzada-Hernández J, Anton J, Martín de Carpi J, et al. Dual latent tuberculosis screening with tuberculin skin tests and QuantiFERON-TB assays before TNF-α inhibitor initiation in children in Spain. *Eur J Pediatr* 2022 .

52 Carvalho AC, Schumacher RF, Bigoni S, et al. Contact investigation based on serial interferon-gamma release assays (IGRA) in children from the hematology-oncology ward after exposure to a patient with pulmonary tuberculosis. *Infection* 2013; **41**: 827-31.

53 Casas S, Muñoz L, Moure R, et al. Comparison of the 2-step tuberculin skin test and the quantiFERON-TB Gold In-Tube Test for the screening of tuberculosis infection before liver transplantation. *Liver Transpl* 2011; **17**: 1205-11.

54 Çekiç C, Aslan F, Vatansever S, et al. Latent tuberculosis screening tests and active tuberculosis infection rates in Turkish inflammatory bowel disease patients under anti-tumor necrosis factor therapy. *Ann Gastroenterol* 2015; **28**: 241-46.

55 Chang B, Park HY, Jeon K, et al. Interferon-γ release assay in the diagnosis of latent tuberculosis infection in arthritis patients treated with tumor necrosis factor antagonists in Korea. *Clin Rheumatol* 2011; **30**: 1535-41.

56 Cheallaigh CN, Fitzgerald I, Grace J, et al. Interferon gamma release assays for the diagnosis of latent TB infection in HIV-infected individuals in a low TB burden country. *PLoS One* 2013; **8**: e53330.

57 Chen DY, Shen GH, Hsieh TY, et al. Effectiveness of the combination of a whole-blood interferon-gamma assay and the tuberculin skin test in detecting latent tuberculosis infection in rheumatoid arthritis patients receiving adalimumab therapy. *Arthritis Rheum* 2008; **59**: 800-06.

58 Chen DY, Shen GH, Chen YM, et al. Biphasic emergence of active tuberculosis in rheumatoid arthritis patients receiving TNFα inhibitors: the utility of IFNγ assay. *Ann Rheum Dis* 2012; **71**: 231-37.

59 Cheng CY, Hui RCY, Hu S, et al. Serial QuantiFERON-TB Gold In-Tube testing for psoriatic patients receiving antitumor necrosis factor-alpha therapy. *Dermatologica Sinica* 2015; **33**: 124-29.

60 Chiappini E, Della Bella C, Bonsignori F, et al. Potential role of M. tuberculosis specific IFN-γ and IL-2 ELISPOT assays in discriminating children with active or latent tuberculosis. *PLoS One* 2012; **7**: e46041.

61 Chien JY, Chiang HT, Lu MC, et al. QuantiFERON-TB Gold Plus Is a More Sensitive Screening Tool than QuantiFERON-TB Gold In-Tube for Latent Tuberculosis Infection among Older Adults in Long-Term Care Facilities. *J Clin Microbiol* 2018; **56**: e00427-18.

62 Chiu HY, Hsueh PR, Tsai TF. Clinical experience of QuantiFERON(®) -TB Gold testing in patients with psoriasis treated with tumour necrosis factor blockers in Taiwan. *Br J Dermatol* 2011; **164**: 553-59.

63 Chkhartishvili N, Kempker RR, Dvali N, et al. Poor agreement between interferon-gamma release assays and the tuberculin skin test among HIV-infected individuals in the country of Georgia. *BMC Infect Dis* 2013; **13**: 513.

64 Cho H, Kim YW, Suh CH, et al. Concordance between the tuberculin skin test and interferon gamma release assay (IGRA) for diagnosing latent tuberculosis infection in patients with systemic lupus erythematosus and patient characteristics associated with an indeterminate IGRA. *Lupus* 2016; **25**: 1341-48.

65 Chuke SO, Yen NT, Laserson KF, et al. Tuberculin Skin Tests versus Interferon-Gamma Release Assays in Tuberculosis Screening among Immigrant Visa Applicants. *Tuberc Res Treat* 2014; **2014**: 217969.

66 Chun JK, Kim CK, Kim HS, et al. The role of a whole blood interferon-gamma assay for the detection of latent tuberculosis infection in Bacille Calmette-Guérin vaccinated children. *Diagn Microbiol Infect Dis* 2008; **62**: 389-94.

67 Chung WK, Zheng ZL, Sung JY, et al. Validity of interferon-γ-release assays for the diagnosis of latent tuberculosis in haemodialysis patients. *Clin Microbiol Infect* 2010; **16**: 960-65.

68 Chung WK, Zheng ZL, Kim HS, et al. Serial testing of interferon-gamma-release assays for the diagnosis of latent tuberculosis in hemodialysis patients. *J Infect* 2010; **61**: 144-49.

69 Cobanoglu N, Ozcelik U, Kalyoncu U, et al. Interferon-gamma assays for the diagnosis of tuberculosis infection before using tumour necrosis factor-alpha blockers. *Int J Tuberc Lung Dis* 2007; **11**: 1177-82.

70 Compagno M, Navarra A, Campogiani L, et al. Latent Tuberculosis Infection in Haematopoietic Stem Cell Transplant Recipients: A Retrospective Italian Cohort Study in Tor Vergata University Hospital, Rome. *Int J Environ Res Public Health* 2022; **19**: 10693.

71 Costantino F, de Carvalho Bittencourt M, Rat AC, et al. Screening for latent tuberculosis infection in patients with chronic inflammatory arthritis: discrepancies between tuberculin skin test and interferon-γ release assay results. *J Rheumatol* 2013; **40**: 1986-93.

72 Critselis E, Amanatidou V, Syridou G, et al. The effect of age on whole blood interferon-gamma release assay response among children investigated for latent tuberculosis infection. *J Pediatr* 2012; **161**: 632-38.

73 Cruz AT, Marape M, Graviss EA, et al. Performance of the QuantiFERON-TB gold interferon gamma release assay among HIV-infected children in Botswana. *J Int Assoc Provid AIDS Care* 2015; **14**: 4-7.

74 Cummings KJ, Smith TS, Shogren ES, et al. Prospective comparison of tuberculin skin test and QuantiFERON-TB Gold In-Tube assay for the detection of latent tuberculosis infection among healthcare workers in a low-incidence setting. *Infect Control Hosp Epidemiol* 2009; **30**: 1123-26.

75 Davarpanah MA, Rasti M, Mehrabani D, et al. Association between PPD and quantiFERON gold TB Test in TB infection and disease among

HIV-Infected individuals in Southern Iran. *Iranian Red Crescent Medical JournalIran. Red Crescent Med.J.* 2009; **11**: 71.

76 de Oliveira Rodrigues M, de Almeida Testa LH, Dos Santos ACF, et al. Latent and active tuberculosis infection in allogeneic hematopoietic stem cell transplant recipients: a prospective cohort study. *Bone Marrow Transplant* 2021; **56**: 2241-47.

77 de Souza-Galvão ML, Latorre I, Altet-Gómez N, et al. Correlation between tuberculin skin test and IGRAs with risk factors for the spread of infection in close contacts with sputum smear positive in pulmonary tuberculosis. *BMC Infect Dis* 2014; **14**: 258.

78 Delgado Naranjo J, Castells Carrillo C, García CMÁ, et al. [Comparative performance of QuantiFERON(®)-TB Gold IT versus tuberculin skin test among contact investigations for latent tuberculosis infection]. *Med Clin (Barc)* 2011; **137**: 289-96.

79 Di Renzi S, Tomao P, Martini A, et al. Screening for tuberculosis among homeless shelter staff. *Am J Infect Control* 2012; **40**: 459-61.

80 Diel R, Loddenkemper R, Niemann S, et al. Negative and positive predictive value of a whole-blood interferon-γ release assay for developing active tuberculosis: an update. *Am J Respir Crit Care Med* 2011; **183**: 88-95.

81 Dirix V, Schepers K, Massinga-Loembe M, et al. Added Value of Long-Term Cytokine Release Assays to Detect Mycobacterium tuberculosis Infection in HIV-Infected Subjects in Uganda. *J Acquir Immune Defic Syndr* 2016; **72**: 344-52.

82 DO H, Ahn SS, Pyo JY, Song JJ, et al. Interferon-gamma Release Assay Results Are Reliable for Screening for Latent Tuberculosis in Antineutrophil Cytoplasmic Antibody-associated Vasculitis. *In Vivo* 2022; **36**: 2884-89.

83 do Valle Leone de Oliveira SM, Ferreira da Silva E, Coimbra Motta-Castro AR, et al. Tuberculosis infection among cocaine crack users in Brazil. *Int J Drug Policy* 2018; **59**: 24-27.

84 Domínguez J, Ruiz-Manzano J, De Souza-Galvão M, et al. Comparison of two commercially available gamma interferon blood tests for immunodiagnosis of tuberculosis. *Clin Vaccine Immunol* 2008; **15**: 168-71.

85 Dorman SE, Belknap R, Graviss EA, et al. Interferon-γ release assays and tuberculin skin testing for diagnosis of latent tuberculosis infection in healthcare workers in the United States. *Am J Respir Crit Care Med* 2014; **189**: 77-87.

86 Doyle JS, Bissessor M, Denholm JT, et al. Latent Tuberculosis screening using interferon-gamma release assays in an Australian HIV-infected cohort: is routine testing worthwhile. *J Acquir Immune Defic Syndr* 2014; **66**: 48-54.

87 Drabe CH, Vestergaard LS, Helleberg M, et al. Performance of Interferon-Gamma and IP-10 Release Assays for Diagnosing Latent Tuberculosis Infections in Patients with Concurrent Malaria in Tanzania. *Am J Trop Med Hyg* 2016; **94**: 728-35.

88 Edathodu J, Varghese B, Alrajhi AA, et al. Diagnostic potential of interferon-gamma release assay to detect latent tuberculosis infection in kidney transplant recipients. *Transpl Infect Dis* 2017; **19**(2).

89 Epstein RL, Bhagavathula M, Saag LA, et al. QuantiFERON(®)-TB Gold In-Tube reliability for immigrants with parasitic infections in Boston, USA. *Int J Tuberc Lung Dis* 2019; **23**: 482-90.

90 Erol S, Ciftci FA, Ciledag A, et al. Do higher cut-off values for tuberculin skin test increase the specificity and diagnostic agreement with interferon gamma release assays in immunocompromised Bacillus Calmette-Guérin vaccinated patients. *Adv Med Sci* 2018; **63**: 237-41.

91 Escalante P, Kooda KJ, Khan R, et al. Diagnosis of latent tuberculosis infection with T-SPOT(®).TB in a predominantly immigrant population

with rheumatologic disorders. *Lung* 2015; **193**: 3-11.

92 Fan WC, Ting WY, Lee MC, et al. Latent TB infection in newly diagnosed lung cancer patients - A multicenter prospective observational study. *Lung Cancer* 2014; **85**: 472-78.

93 Fernández-Blázquez A, Argüelles Menéndez P, Sabater-Cabrera C, et al. Diagnosis of Tuberculous Infection in Immunosuppressed Patients and/or Candidates for Biologics Using a Combination of 2 IGRA Tests: T-SPOT.TB/QuantiFERON TB Gold In-Tube vs. T-SPOT.TB/QuantiFERON TB Gold Plus. *Arch Bronconeumol* 2022; **58**: 305-10.

94 Ferrarini MA, Spina FG, Weckx LY, et al. Rate of tuberculosis infection in children and adolescents with household contact with adults with active pulmonary tuberculosis as assessed by tuberculin skin test and interferon-gamma release assays. *Epidemiol Infect* 2016; **144**: 712-23.

95 Fong KS, Tomford JW, Teixeira L, et al. Challenges of interferon-γ release assay conversions in serial testing of health-care workers in a TB control program. *Chest* 2012; **142**: 55-62.

96 Fox BD, Kramer MR, Mor Z, et al. The QuantiFERON-TB-GOLD assay for tuberculosis screening in healthcare workers: a cost-comparison analysis. *Lung* 2009; **187**: 413-19.

97 Freeman JT, Marshall RJ, Newton S, et al. Screening for Mycobacterium tuberculosis infection among healthcare workers in New Zealand: prospective comparison between the tuberculin skin test and the QuantiFERON-TB Gold In-Tube assay. *N Z Med J* 2012; **125**: 21-29.

98 Fujita K, Elkington P, Redelman-Sidi G, et al. Serial interferon-gamma release assay in lung cancer patients receiving immune checkpoint inhibitors: a prospective cohort study. *Cancer Immunol Immunother* 2022; **71**(11):2757-2764..

99 Gabriele F, Trachana M, Simitsopoulou M, et al. Performance of QuantiFERON®-TB Gold In-Tube assay in children receiving disease modifying anti-rheumatic drugs. *World J Pediatr* 2017; **13**: 472-78.

100 Gaensbauer J, Gonzales B, Belknap R, et al. Interferon-Gamma Release Assay-Based Screening for Pediatric Latent Tuberculosis Infection in an Urban Primary Care Network. *J Pediatr* 2018; **200**: 202-09.

101 Gaensbauer J, Young J, Harasaki C, et al. Interferon-Gamma Release Assay Testing in Children Younger Than 2 Years in a US-Based Health System. *Pediatr Infect Dis J* 2020; **39**: 803-07.

102 Galindo JL, Galeano AC, Suarez-Zamora DA, et al. Comparison of the QuantiFERON-TB and tuberculin skin test for detection of latent tuberculosis infection in cancer patients in a developing country. *ERJ Open Res* 2019; **5**(4):00258-2018.

103 Gao L, Lu W, Bai L, et al. Latent tuberculosis infection in rural China: baseline results of a population-based, multicentre, prospective cohort study. *Lancet Infect Dis* 2015; **15**: 310-19.

104 Garazzino S, Galli L, Chiappini E, et al. Performance of interferon-γ release assay for the diagnosis of active or latent tuberculosis in children in the first 2 years of age: a multicenter study of the Italian Society of Pediatric Infectious Diseases. *Pediatr Infect Dis J* 2014; **33**: e226-31.

105 García-Gasalla M, Fernández-Baca V, Juan-Mas A, et al. Use of Quantiferon-TB-Gold in Tube(®) test for detecting latent tuberculosis in patients considered as candidates for anti-TNF therapy in routine clinical practice. *Enferm Infecc Microbiol Clin* 2013; **31**: 76-81.

106 Gebreegziabiher D, Desta K, Howe R, et al. Helminth infection increases the probability of indeterminate QuantiFERON gold in tube results in

pregnant women. *Biomed Res Int* 2014; **2014**: 364137.

107 Goletti D, Navarra A, Petruccioli E, et al. Latent tuberculosis infection screening in persons newly-diagnosed with HIV infection in Italy: A multicentre study promoted by the Italian Society of Infectious and Tropical Diseases. *Int J Infect Dis* 2020; **92**: 62-68.

108 González-Moreno J, García-Gasalla M, Losada-López I, et al. IGRA testing in patients with immune-mediated inflammatory diseases: which factors influence the results. *Rheumatol Int* 2018; **38**: 267-73.

109 Gonzślez-Moreno J, García-Gasalla M, Gállego-Lezaun C, et al. Role of QuantiFERON(®)-TB Gold In-Tube in tuberculosis contact investigation: experience in a tuberculosis unit. *Infect Dis (Lond)* 2015; **47**: 244-51.

110 Goodwin DJ, Mazurek GH, Campbell BH, et al. Automation of an interferon-γ release assay and comparison to the tuberculin skin test for screening basic military trainees for Mycobacterium tuberculosis infection. *Mil Med* 2014; **179**: 333-41.

111 Grant J, Jastrzebski J, Johnston J, et al. Interferon-gamma release assays are a better tuberculosis screening test for hemodialysis patients: A study and review of the literature. *Can J Infect Dis Med Microbiol* 2012; **23**: 114-16.

112 Gray J, Reves R, Johnson S, et al. Identification of false-positive QuantiFERON-TB Gold In-Tube assays by repeat testing in HIV-infected patients at low risk for tuberculosis. *Clin Infect Dis* 2012; **54**: e20-23.

113 Greenberg JD, Reddy SM, Schloss SG, et al. Comparison of an in vitro tuberculosis interferon-gamma assay with delayed-type hypersensitivity testing for detection of latent Mycobacterium tuberculosis: a pilot study in rheumatoid arthritis. *J Rheumatol* 2008; **35**: 770-75.

114 Greveson K, Goodhand J, Capocci S, et al. Yield and cost effectiveness of mycobacterial infection detection using a simple IGRA-based protocol in UK subjects with inflammatory bowel disease suitable for anti-TNFα therapy. *J Crohns Colitis* 2013; **7**: 412-18.

115 Grimes CZ, Hwang LY, Williams ML, et al. Tuberculosis infection in drug users: interferon-gamma release assay performance. *Int J Tuberc Lung Dis* 2007; **11**: 1183-89.

116 Grinsdale JA, Islam S, Tran OC, et al. Interferon-Gamma Release Assays and Pediatric Public Health Tuberculosis Screening: The San Francisco Program Experience 2005 to 2008. *J Pediatric Infect Dis Soc* 2016; **5**: 122-30.

117 Gunluoglu G, Seyhan EC, Kazancioglu R, et al. Diagnosing latent tuberculosis in immunocompromised patients measuring blood IP-10 production capacity: an analysis of chronic renal failure patients. *Intern Med* 2015; **54**: 465-72.

118 Hadaya K, Bridevaux PO, Roux-Lombard P, et al. Contribution of interferon-γ release assays (IGRAs) to the diagnosis of latent tuberculosis infection after renal transplantation. *Transplantation* 2013; **95**: 1485-90.

119 Hakimian S, Popov Y, Rupawala AH, et al. The conundrum of indeterminate QuantiFERON-TB Gold results before anti-tumor necrosis factor initiation. *Biologics* 2018; **12**: 61-67.

120 Hand J, Sigel K, Huprikar S, et al. Tuberculosis after liver transplantation in a large center in New York City: QuantiFERON(®) -TB Gold-based pre-transplant screening performance and active tuberculosis post-transplant. *Transpl Infect Dis* 2018; **20**: e12845.

121 Haustein T, Ridout DA, Hartley JC, et al. The likelihood of an indeterminate test result from a whole-blood interferon-gamma release assay for the diagnosis of Mycobacterium tuberculosis infection in children correlates with age and immune status. *Pediatr Infect Dis J* 2009; **28**: 669-73.

122 He GX, Wang LX, Chai SJ, et al. Risk factors associated with tuberculosis infection among health care workers in Inner Mongolia, China. *Int J Tuberc Lung Dis* 2012; **16**: 1485-91.

123 He G, Li Y, Zhao F, et al. The Prevalence and Incidence of Latent Tuberculosis Infection and Its Associated Factors among Village Doctors in China. *PLoS One* 2015; **10**: e0124097.

124 Helwig U, Müller M, Hedderich J, et al. Corticosteroids and immunosuppressive therapy influence the result of QuantiFERON TB Gold testing in inflammatory bowel disease patients. *J Crohns Colitis* 2012; **6**: 419-24.

125 Hermansen T, Lillebaek T, Hansen AB, et al. QuantiFERON–TB Gold In-Tube test performance in Denmark. *Tuberculosis (Edinb)* 2014; **94**: 616-21.

126 Hesseling AC, Mandalakas AM, Kirchner HL, et al. Highly discordant T cell responses in individuals with recent exposure to household tuberculosis. *Thorax* 2009; **64**: 840-46.

127 Higuchi K, Kondo S, Wada M, et al. Contact investigation in a primary school using a whole blood interferon-gamma assay. *J Infect* 2009; **58**: 352-57.

128 Ho CS, Feng PI, Narita M, et al. Comparison of three tests for latent tuberculosis infection in high-risk people in the USA: an observational cohort study. *Lancet Infect Dis* 2022; **22**: 85-96.

129 Hoffmann M, Tsinalis D, Vernazza P, et al. Assessment of an Interferon-gamma release assay for the diagnosis of latent tuberculosis infection in haemodialysis patient. *Swiss Med Wkly* 2010; **140**: 286-92.

130 Hotta K, Ogura T, Nishii K, et al. Whole blood interferon-gamma assay for baseline tuberculosis screening among Japanese healthcare students. *PLoS One* 2007; **2**: e803.

131 Howley MM, Painter JA, Katz DJ, et al. Evaluation of QuantiFERON-TB gold in-tube and tuberculin skin tests among immigrant children being screened for latent tuberculosis infection. *Pediatr Infect Dis J* 2015; **34**: 35-39.

132 Hradsky O, Ohem J, Zarubova K, et al. Disease activity is an important factor for indeterminate interferon-γ release assay results in children with inflammatory bowel disease. *J Pediatr Gastroenterol Nutr* 2014; **58**: 320-24.

133 Hsia EC, Schluger N, Cush JJ, et al. Interferon-γ release assay versus tuberculin skin test prior to treatment with golimumab, a human anti-tumor necrosis factor antibody, in patients with rheumatoid arthritis, psoriatic arthritis, or ankylosing spondylitis. *Arthritis Rheum* 2012; **64**: 2068-77.

134 Hsiao CY, Chiu HY, Wang TS, et al. Serial QuantiFERON-TB Gold testing in patients with psoriasis treated with ustekinumab. *PLoS One* 2017; **12**: e0184178.

135 Huang SF, Chen MH, Wang FD, et al. Efficacy of isoniazid salvage therapy for latent tuberculosis infection in patients with immune-mediated inflammatory disorders - A retrospective cohort study in Taiwan. *J Microbiol Immunol Infect* 2018; **51**: 784-93.

136 Huang CC, Jerry Teng CL, Wu MF, et al. Features of indeterminate results of QuantiFERON-TB Gold In-Tube test in patients with haematological malignancies. *Ther Adv Hematol* 2021; **12**: 20406207211028437.

137 Huerga H, Sanchez-Padilla E, Melikyan N, et al. High prevalence of infection and low incidence of disease in child contacts of patients with drug-resistant tuberculosis: a prospective cohort study. *Arch Dis Child* 2019; **104**: 622-28.

138 Hung WT, Lee SS, Sy CL, et al. Prevalence of latent tuberculosis infection in BCG-vaccinated healthcare workers by using an interferon-gamma release assay and the tuberculin skin test in an intermediate tuberculosis burden country. *J Microbiol Immunol Infect* 2015; **48**: 147-52.

139 Hwang HJ, Park JH, Nam S, et al. Characteristics of a large latent tuberculous infection screening programme using QuantiFERON(®)-TB Gold in Korea. *Int J Tuberc Lung Dis* 2018; **22**: 504-09.

140 Igari H, Ishikawa S, Nakazawa T, et al. Lymphocyte subset analysis in QuantiFERON-TB Gold Plus and T-Spot.TB for latent tuberculosis infection in rheumatoid arthritis. *J Infect Chemother* 2018; **24**: 110-16.

141 Igari H, Akutsu N, Ishikawa S, et al. Positivity rate of interferon-γ release assays for estimating the prevalence of latent tuberculosis infection in renal transplant recipients in Japan. *J Infect Chemother* 2019; **25**: 537-42.

142 Igari H, Takayanagi S, Yahaba M, et al. Prevalence of positive IGRAs and innate immune system in HIV-infected individuals in Japan. *J Infect Chemother* 2021; **27**: 592-97.

143 Inanc N, Aydin SZ, Karakurt S, et al. Agreement between Quantiferon-TB gold test and tuberculin skin test in the identification of latent tuberculosis infection in patients with rheumatoid arthritis and ankylosing spondylitis. *J Rheumatol* 2009; **36**: 2675-81.

144 Ishikawa S, Igari H, Akutsu N, et al. Comparison of interferon-γ release assays, QuantiFERON TB-GIT and T-Spot.TB, in renal transplantation. *J Infect Chemother* 2017; **23**: 468-73.

145 Iwagaitsu S, Naniwa T, Maeda S, et al. A comparative analysis of two interferon-γ releasing assays to detect past tuberculosis infections in Japanese rheumatoid arthritis patients. *Mod Rheumatol* 2016; **26**: 690-95.

146 Jafri SM, Singal AG, Kaul D, et al. Detection and management of latent tuberculosis in liver transplant patients. *Liver Transpl* 2011; **17**: 306-14.

147 Janssens JP, Roux-Lombard P, Perneger T, et al. Contribution of a IFN-gamma assay in contact tracing for tuberculosis in a low-incidence, high immigration area. *Swiss Med Wkly* 2008; **138**: 585-93.

148 Jones S, de Gijsel D, Wallach FR, et al. Utility of QuantiFERON-TB Gold in-tube testing for latent TB infection in HIV-infected individuals. *Int J Tuberc Lung Dis* 2007; **11**: 1190-95.

149 Jonnalagadda S, Lohman Payne B, Brown E, et al. Latent tuberculosis detection by interferon γ release assay during pregnancy predicts active tuberculosis and mortality in human immunodeficiency virus type 1-infected women and their children. *J Infect Dis* 2010; **202**: 1826-35.

150 Joshi M, Monson TP, Joshi A, et al. IFN-γ release assay conversions and reversions. Challenges with serial testing in U.S. health care workers. *Ann Am Thorac Soc* 2014; **11**: 296-302.

151 Jung YJ, Lyu J, Yoo B, et al. Combined use of a TST and the T-SPOT®.TB assay for latent tuberculosis infection diagnosis before anti-TNF-α treatment. *Int J Tuberc Lung Dis* 2012; **16**: 1300-06.

152 Jung HJ, Kim TJ, Kim HS, et al. Analysis of predictors influencing indeterminate whole-blood interferon-gamma release assay results in

patients with rheumatic diseases. *Rheumatol Int* 2014; **34**: 1711-20.

153 Jung YJ, Lee JY, Jo KW, et al. The 'either test positive' strategy for latent tuberculous infection before anti-tumour necrosis factor treatment. *Int J Tuberc Lung Dis* 2014; **18**: 428-34.

154 Jung YJ, Woo HI, Jeon K, et al. The Significance of Sensitive Interferon Gamma Release Assays for Diagnosis of Latent Tuberculosis Infection in Patients Receiving Tumor Necrosis Factor-α Antagonist Therapy. *PLoS One* 2015; **10**: e0141033.

155 Jung J, Jhun BW, Jeong M, et al. Is the New Interferon-Gamma Releasing Assay Beneficial for the Diagnosis of Latent and Active Mycobacterium tuberculosis Infections in Tertiary Care Setting. *J Clin Med* 2021; **10**: 1376.

156 Juno JA, Waruk JLM, Mesa C, et al. Maintenance of Mycobacterium tuberculosis-specific T cell responses in End Stage Renal Disease (ESRD) and implications for diagnostic efficacy. *Clin Immunol* 2016; **168**: 55-63.

157 Kabeer BS, Sikhamani R, Raja A. Comparison of interferon gamma-inducible protein-10 and interferon gamma-based QuantiFERON TB Gold assays with tuberculin skin test in HIV-infected subjects. *Diagn Microbiol Infect Dis* 2011; **71**: 236-43.

158 Kall MM, Coyne KM, Garrett NJ, et al. Latent and subclinical tuberculosis in HIV infected patients: a cross-sectional study. *BMC Infect Dis* 2012; **12**: 107.

159 Kampmann B, Seddon JA, Paton J, et al. Evaluating UK National Guidance for Screening of Children for Tuberculosis. A Prospective Multicenter Study. *Am J Respir Crit Care Med* 2018; **197**: 1058-64.

160 Kasambira TS, Shah M, Adrian PV, et al. QuantiFERON-TB Gold In-Tube for the detection of Mycobacterium tuberculosis infection in children with household tuberculosis contact. *Int J Tuberc Lung Dis* 2011; **15**: 628-34.

161 Katsenos S, Nikolopoulou M, Konstantinidis AK, et al. Interferon-gamma release assay clarifies the effect of bacille Calmette-Guérin vaccination in Greek army recruits. *Int J Tuberc Lung Dis* 2010; **14**: 545-50.

162 Katyal M, Leibowitz R, Venters H. IGRA-Based Screening for Latent Tuberculosis Infection in Persons Newly Incarcerated in New York City Jails. *J Correct Health Care* 2018; **24**: 156-70.

163 Kaur M, Singapura P, Kalakota N, et al. Factors That Contribute to Indeterminate Results From the QuantiFERON-TB Gold In-Tube Test in Patients With Inflammatory Bowel Disease. *Clin Gastroenterol Hepatol* 2018; **16**: 1616-21.e1.

164 Kay AW, DiNardo AR, Dlamini Q, et al. Evaluation of the QuantiFERON-Tuberculosis Gold Plus Assay in Children with Tuberculosis Disease or Following Household Exposure to Tuberculosis. *Am J Trop Med Hyg* 2019; **100**: 540-43.

165 Khawcharoenporn T, Apisarnthanarak A, Phetsuksiri B, et al. Tuberculin skin test and QuantiFERON-TB Gold In-tube Test for latent tuberculosis in Thai HIV-infected adults. *Respirology* 2015; **20**: 340-47.

166 Khawcharoenporn T, Apisarnthanarak A, Sangkitporn S, et al. Tuberculin Skin Test and QuantiFERON(®)-TB Gold In-Tube Test for Diagnosing Latent Tuberculosis Infection among Thai Healthcare Workers. *Jpn J Infect Dis* 2016; **69**: 224-30.

167 Kim EY, Lim JE, Jung JY, et al. Performance of the tuberculin skin test and interferon-gamma release assay for detection of tuberculosis infection in immunocompromised patients in a BCG-vaccinated population. *BMC Infect Dis* 2009; **9**: 207.

168 Kim SH, Lee SO, Park IA, et al. Diagnostic usefulness of a T cell-based assay for latent tuberculosis infection in kidney transplant candidates before transplantation. *Transpl Infect Dis* 2010; **12**: 113-19.

169 Kim YJ, Kim SI, Kim YR, et al. Predictive value of interferon-γ ELISPOT assay in HIV 1-infected patients in an intermediate tuberculosis-endemic area. *AIDS Res Hum Retroviruses* 2012; **28**: 1038-43.

170 Kim HC, Jo KW, Jung YJ, et al. Diagnosis of latent tuberculosis infection before initiation of anti-tumor necrosis factor therapy using both tuberculin skin test and QuantiFERON-TB Gold In Tube assay. *Scand J Infect Dis* 2014; **46**: 763-69.

171 Kim MJ, Nam EY, Park KU, et al. Limited usefulness of the interferon-gamma release assay in patients with HIV infections and negative tuberculin skin test results in a country with an intermediate tuberculosis burden. *Infect Dis (Lond)* 2017; **49**: 420-22.

172 Kim H, Kim SH, Jung JH, et al. The usefulness of quantitative interferon-gamma releasing assay response for predicting active tuberculosis in kidney transplant recipients: A quasi-experimental study. *J Infect* 2020; **81**: 403-10.

173 Kim KH, Kang JM, Ahn JG. Low-dose steroids are associated with indeterminate QuantiFERON-TB Gold In-Tube assay results in immunocompetent children. *Sci Rep* 2021; **11**: 6468.

174 Kim EY, Lim JE, Jung JY, et al. Performance of the tuberculin skin test and interferon-gamma release assay for detection of tuberculosis infection in immunocompromised patients in a BCG-vaccinated population. *BMC Infect Dis* 2009; **9**: 207.

175 Kim JH, Cho SK, Han M, et al. Factors influencing discrepancies between the QuantiFERON-TB gold in tube test and the tuberculin skin test in Korean patients with rheumatic diseases. *Semin Arthritis Rheum* 2013; **42**: 424-32.

176 Kim JH, Won S, Choi CB, et al. Evaluation of the usefulness of interferon-gamma release assays and the tuberculin skin test for the detection of latent Mycobacterium tuberculosis infections in Korean rheumatic patients who are candidates for biologic agents. *Int J Rheum Dis* 2015; **18**: 315-22.

177 Kim JS, Cho JH, Park GY, et al. Comparison of QuantiFERON-TB Gold with tuberculin skin test for detection of latent tuberculosis infection before kidney transplantation. *Transplant Proc* 2013; **45**: 2899-902.

178 Kim KH, Lee SW, Chung WT, et al. Serial interferon-gamma release assays for the diagnosis of latent tuberculosis infection in patients treated with immunosuppressive agents. *Korean J Lab Med* 2011; **31**: 271-78.

179 Kim SH, Lee SO, Park JB, et al. A prospective longitudinal study evaluating the usefulness of a T-cell-based assay for latent tuberculosis infection in kidney transplant recipients. *Am J Transplant* 2011; **11**: 1927-35.

180 Kim SH, Lee SO, Park IA, et al. Isoniazid treatment to prevent TB in kidney and pancreas transplant recipients based on an interferon-γ-releasing assay: an exploratory randomized controlled trial. *J Antimicrob Chemother* 2015; **70**: 1567-72.

181 Kim SY, Jung GS, Kim SK, et al. Comparison of the tuberculin skin test and interferon-γ release assay for the diagnosis of latent tuberculosis infection before kidney transplantation. *Infection* 2013; **41**: 103-10.

182 Klein M, Jarosová K, Forejtová S, et al. Quantiferon TB Gold and tuberculin skin tests for the detection of latent tuberculosis infection in patients treated with tumour necrosis factor alpha blocking agents. *Clin Exp Rheumatol* 2013; **31**: 111-17.

183 Kleinert S, Tony HP, Krueger K, et al. Screening for latent tuberculosis infection: performance of tuberculin skin test and interferon-γ release assays under real-life conditions. *Ann Rheum Dis* 2012; **71**: 1791-95.

184 Kobashi Y, Mouri K, Obase Y, et al. Clinical evaluation of QuantiFERON TB-2G test for immunocompromised patients. *Eur Respir J* 2007; **30**: 945-50.

185 Kobashi Y, Shimizu H, Ohue Y, et al. Comparison of T-cell interferon-gamma release assays for Mycobacterium tuberculosis-specific antigens in patients with active and latent tuberculosis. *Lung* 2010; **188**: 283-87.

186 Koesoemadinata RC, McAllister SM, Soetedjo NNM, et al. Latent TB infection and pulmonary TB disease among patients with diabetes mellitus in Bandung, Indonesia. *Trans R Soc Trop Med Hyg* 2017; **111**: 81-89.

187 Korra E, Dwedar I, Abdelfattah E, et al. Role of Quantiferon gold assay in the detection and follow-up of treatment of latent tuberculosis infection in some acquired immune-compromised patients. *Egyptian Journal of Chest Diseases and Tuberculosis* 2019; **68**: 498-504.

188 Kurti Z, Lovasz BD, Gecse KB, et al. Tuberculin Skin Test and Quantiferon in BCG Vaccinated, Immunosuppressed Patients with Moderate-to-Severe Inflammatory Bowel Disease. *J Gastrointestin Liver Dis* 2015; **24**: 467-72.

189 Kussen GM, Dalla-Costa LM, Rossoni A, et al. Interferon-gamma release assay versus tuberculin skin test for latent tuberculosis infection among HIV patients in Brazil. *Braz J Infect Dis* 2016; **20**: 69-75.

190 Kwakernaak AJ, Houtman PM, Weel JF, et al. A comparison of an interferon-gamma release assay and tuberculin skin test in refractory inflammatory disease patients screened for latent tuberculosis prior to the initiation of a first tumor necrosis factor α inhibitor. *Clin Rheumatol* 2011; **30**: 505-10.

191 LaCourse SM, Cranmer LM, Matemo D, et al. Effect of Pregnancy on Interferon Gamma Release Assay and Tuberculin Skin Test Detection of Latent TB Infection Among HIV-Infected Women in a High Burden Setting. *J Acquir Immune Defic Syndr* 2017; **75**: 128-36.

192 Lai HC, Chang CH, Cheng KS, et al. QuantiFERON-TB Gold Test Conversion Is Associated with Active Tuberculosis Development in Inflammatory Bowel Disease Patients Treated with Biological Agents: An Experience of a Medical Center in Taiwan. *Gastroenterol Res Pract* 2019; **2019**: 7132875.

193 Lange B, Vavra M, Kern WV, et al. Development of tuberculosis in immunocompromised patients with a positive tuberculosis-specific IGRA. *Int J Tuberc Lung Dis* 2012; **16**: 492-95.

194 Latorre I, Martínez-Lacasa X, Font R, et al. IFN-γ response on T-cell based assays in HIV-infected patients for detection of tuberculosis infection. *BMC Infect Dis* 2010; **10**: 348.

195 Latorre I, Carrascosa JM, Vilavella M, et al. Diagnosis of tuberculosis infection by interferon-gamma release assays in patients with psoriasis. *J Infect* 2014; **69**: 600-06.

196 Lebina L, Abraham PM, Milovanovic M, et al. Latent tuberculous infection in schoolchildren and contact tracing in Matlosana, North West Province, South Africa. *Int J Tuberc Lung Dis* 2015; **19**: 1290-92.

197 Lee H, Park HY, Jeon K, et al. QuantiFERON-TB Gold In-Tube assay for screening arthritis patients for latent tuberculosis infection before

starting anti-tumor necrosis factor treatment. *PLoS One* 2015; **10**: e0119260.

198 Lee JH, Sohn HS, Chun JH, et al. Poor agreement between QuantiFERON-TB Gold test and tuberculin skin test results for the diagnosis of latent tuberculosis infection in rheumatoid arthritis patients and healthy controls. *Korean J Intern Med* 2014; **29**: 76-84.

199 Lee K, Han MK, Choi HR, et al. Annual incidence of latent tuberculosis infection among newly employed nurses at a tertiary care university hospital. *Infect Control Hosp Epidemiol* 2009; **30**: 1218-22.

200 Lee SH, Lew WJ, Kim HJ, et al. Serial interferon-gamma release assays after rifampicin prophylaxis in a tuberculosis outbreak. *Respir Med* 2010; **104**: 448-53.

201 Lee SS, Chou KJ, Su IJ, et al. High prevalence of latent tuberculosis infection in patients in end-stage renal disease on hemodialysis: Comparison of QuantiFERON-TB GOLD, ELISPOT, and tuberculin skin test. *Infection* 2009; **37**: 96-102.

202 Lee SS, Chou KJ, Dou HY, et al. High prevalence of latent tuberculosis infection in dialysis patients using the interferon-gamma release assay and tuberculin skin test. *Clin J Am Soc Nephrol* 2010; **5**: 1451-57.

203 Lee SS, Lin HH, Tsai HC, et al. A Clinical Algorithm to Identify HIV Patients at High Risk for Incident Active Tuberculosis: A Prospective 5-Year Cohort Study. *PLoS One* 2015; **10**: e0135801.

204 Lee YM, Lee SO, Choi SH, et al. A prospective longitudinal study evaluating the usefulness of the interferon-gamma releasing assay for predicting active tuberculosis in allogeneic hematopoietic stem cell transplant recipients. *J Infect* 2014; **69**: 165-73.

205 Legesse M, Ameni G, Mamo G, et al. Community-based cross-sectional survey of latent tuberculosis infection in Afar pastoralists, Ethiopia, using QuantiFERON-TB Gold In-Tube and tuberculin skin test. *BMC Infect Dis* 2011; **11**: 89.

206 Leidl L, Mayanja-Kizza H, Sotgiu G, et al. Relationship of immunodiagnostic assays for tuberculosis and numbers of circulating CD4+ T-cells in HIV infection. *Eur Respir J* 2010; **35**: 619-26.

207 Lempp JM, Zajdowicz MJ, Hankinson AL, et al. Assessment of the QuantiFERON-TB Gold In-Tube test for the detection of Mycobacterium tuberculosis infection in United States Navy recruits. *PLoS One* 2017; **12**: e0177752.

208 Lien LT, Hang NT, Kobayashi N, et al. Prevalence and risk factors for tuberculosis infection among hospital workers in Hanoi, Viet Nam. *PLoS One* 2009; **4**: e6798.

209 Lighter J, Rigaud M, Eduardo R, et al. Latent tuberculosis diagnosis in children by using the QuantiFERON-TB Gold In-Tube test. *Pediatrics* 2009; **123**: 30-37.

210 Lighter-Fisher J, Surette AM. Performance of an interferon-gamma release assay to diagnose latent tuberculosis infection during pregnancy. *Obstet Gynecol* 2012; **119**: 1088-95.

211 Lin WC, Lin HH, Lee SS, et al. Prevalence of latent tuberculosis infection in persons with and without human immunodeficiency virus infection using two interferon-gamma release assays and tuberculin skin test in a low human immunodeficiency virus prevalence, intermediate tuberculosis-burden country. *J Microbiol Immunol Infect* 2016; **49**: 729-36.

212 Lin KY, Yang CJ, Sun HY, et al. Care cascade of tuberculosis infection treatment for people living with HIV in the era of antiretroviral therapy

scale-up. *Sci Rep* 2022; **12**: 16136.

213 Lombardi G, Tengattini V, Dal Monte P, et al. Does biological therapy affect interferon-γ release assay response? A long-term follow-up of patients with psoriasis using QuantiFERON-TB. *Br J Dermatol* 2015; **172**: 798-800.

214 Lombardi G, Petrucci R, Corsini I, et al. Quantitative Analysis of Gamma Interferon Release Assay Response in Children with Latent and Active Tuberculosis. *J Clin Microbiol* 2018; **56** .

215 Loutet MG, Burman M, Jayasekera N, et al. National roll-out of latent tuberculosis testing and treatment for new migrants in England: a retrospective evaluation in a high-incidence area. *Eur Respir J* 2018; **51**: e01360-17.

216 Lucas M, Nicol P, McKinnon E, et al. A prospective large-scale study of methods for the detection of latent Mycobacterium tuberculosis infection in refugee children. *Thorax* 2010; **65**: 442-48.

217 Lucet JC, Abiteboul D, Estellat C, et al. Interferon-γ release assay vs. tuberculin skin test for tuberculosis screening in exposed healthcare workers: a longitudinal multicenter comparative study. *Infect Control Hosp Epidemiol* 2015; **36**: 569-74.

218 Luetkemeyer AF, Charlebois ED, Flores LL, et al. Comparison of an interferon-gamma release assay with tuberculin skin testing in HIV-infected individuals. *Am J Respir Crit Care Med* 2007; **175**: 737-42.

219 Machado A Jr, Emodi K, Takenami I, et al. Analysis of discordance between the tuberculin skin test and the interferon-gamma release assay. *Int J Tuberc Lung Dis* 2009; **13**: 446-53.

220 Maden E, Bekci TT, Kesli R, et al. Evaluation of performance of quantiferon assay and tuberculin skin test in end stage renal disease patients receiving hemodialysis. *New Microbiol* 2011; **34**: 351-56.

221 Mahmoudi S, Pourakbari B, Sadeghi RH, et al. High prevalence of latent tuberculosis in hematopoietic stem cell transplant recipients: A First Report. *Pediatr Transplant* 2020; **24**: e13770.

222 Mahomed H, Hawkridge T, Verver S, et al. Predictive factors for latent tuberculosis infection among adolescents in a high-burden area in South Africa. *Int J Tuberc Lung Dis* 2011; **15**: 331-36.

223 Mancuso JD, Mazurek GH, Tribble D, et al. Discordance among commercially available diagnostics for latent tuberculosis infection. *Am J Respir Crit Care Med* 2012; **185**: 427-34.

224 Mancuso JD, Diffenderfer JM, Ghassemieh BJ, et al. The Prevalence of Latent Tuberculosis Infection in the United States. *Am J Respir Crit Care Med* 2016; **194**: 501-09.

225 Mandalakas AM, Hesseling AC, Chegou NN, et al. High level of discordant IGRA results in HIV-infected adults and children. *Int J Tuberc Lung Dis* 2008; **12**: 417-23.

226 Mandalakas AM, Kirchner HL, Walzl G, et al. Optimizing the detection of recent tuberculosis infection in children in a high tuberculosis-HIV burden setting. *Am J Respir Crit Care Med* 2015; **191**: 820-30.

227 Mantri AK, Meena P, Puri AS, et al. Comparison of Interferon-Gamma Release Assay and Tuberculin Skin Test for the Screening of Latent Tuberculosis in Inflammatory Bowel Disease Patients: Indian Scenario. *Tuberc Res Treat* 2021; **2021**: 6682840.

228 Manuel O, Humar A, Preiksaitis J, et al. Comparison of quantiferon-TB gold with tuberculin skin test for detecting latent tuberculosis infection prior to liver transplantation. *Am J Transplant* 2007; **7**: 2797-801.

229 Mardani M, Tabarsi P, Mohammadtaheri Z, et al. Performance of QuantiFERON-TB Gold test compared to tuberculin skin test in detecting latent tuberculosis infection in HIV- positive individuals in Iran. *Ann Thorac Med* 2010; **5**: 43-46.

230 Mariette X, Baron G, Tubach F, et al. Influence of replacing tuberculin skin test with ex vivo interferon γ release assays on decision to administer prophylactic antituberculosis antibiotics before anti-TNF therapy. *Ann Rheum Dis* 2012; **71**: 1783-90.

231 Marino A, Chiappini E, Cimaz R, et al. Prebiologic Therapy Tuberculosis Screening Experience in a Pediatric Rheumatology Center: TST and IGRA Are Both Necessary. *Pediatr Infect Dis J* 2017; **36**: 440-41.

232 Maritsi D, Al-Obadi M, Brogan PA, et al. The Performance of Quantiferon TB Gold In-Tube as a Screening Tool in Paediatric Rheumatology prior to Initiation of Infliximab: A Single Centre's Experience. *ISRN Rheumatol* 2011; **2011**: 505171.

233 Marquez C, Chamie G, Achan J, et al. Tuberculosis Infection in Early Childhood and the Association with HIV-exposure in HIV-uninfected Children in Rural Uganda. *Pediatr Infect Dis J* 2016; **35**: 524-29.

234 Martinez-Lopez D, Osorio-Chávez J, Álvarez-Reguera C, et al. Epidemiology of latent tuberculosis infection in patients with rheumatic immune-mediated diseases: Single university study of 1117 patients. *Arthritis and RheumatologyArthritis Rheum.* 2021; **73**: 2140.

235 Martínez-Morillo M, Mínguez S, Mateo L, et al. Interferon-gamma release assays in rheumatic patients: Baseline study and in the course of anti-tumor necrosis factor-alpha agents. *Annals of the Rheumatic Disease* 2013; **71**(7).

236 Martyn-Simmons CL, Mee JB, Kirkham BW, et al. Evaluating the use of the interferon-γ response to Mycobacterium tuberculosis-specific antigens in patients with psoriasis prior to antitumour necrosis factor-α therapy: a prospective head-to-head cross-sectional study. *Br J Dermatol* 2013; **168**: 1012-18.

237 Mathad JS, Bhosale R, Sangar V, et al. Pregnancy differentially impacts performance of latent tuberculosis diagnostics in a high-burden setting. *PLoS One* 2014; **9**: e92308.

238 Mathad JS, Bhosale R, Balasubramanian U, et al. Quantitative IFN-γ and IL-2 Response Associated with Latent Tuberculosis Test Discordance in HIV-infected Pregnant Women. *Am J Respir Crit Care Med* 2016; **193**: 1421-28.

239 Matsumura R, Igari H, Nakazawa T, et al. Comparative utility of interferon-γ release assay, QuantiFERON(®) TB-GIT and T-SPOT(®).TB in rheumatoid arthritis. *Int J Tuberc Lung Dis* 2016; **20**: 1546-53.

240 Matulis G, Jüni P, Villiger PM, et al. Detection of latent tuberculosis in immunosuppressed patients with autoimmune diseases: performance of a Mycobacterium tuberculosis antigen-specific interferon gamma assay. *Ann Rheum Dis* 2008; **67**: 84-90.

241 Mehta B, Zapantis E, Petryna O, et al. Screening Optimization of Latent Tuberculosis Infection in Rheumatoid Arthritis Patients. *Arthritis* 2015; **2015**: 569620.

242 Meinerz G, Silva C, Dorsdt D, et al. Latent tuberculosis screening before kidney transplantation in the South of Brazil. *J Bras Nefrol* 2021; **43**: 520-29.

243 Méndez-Echevarría A, González-Muñoz M, Mellado MJ, et al. Interferon-γ release assay for the diagnosis of tuberculosis in children. *Arch Dis Child* 2012; **97**: 514-16.

244 Mensah GI, Sowah SA, Yeboah NY, et al. Utility of QuantiFERON tuberculosis gold-in-tube test for detecting latent tuberculosis infection among close household contacts of confirmed tuberculosis patients in Accra, Ghana. *Int J Mycobacteriol* 2017; **6**: 27-33.

245 Milman N, Søborg B, Svendsen CB, et al. Quantiferon test for tuberculosis screening in sarcoidosis patients. *Scand J Infect Dis* 2011; **43**: 728-35.

246 Mínguez S, Latorre I, Mateo L, et al. Interferon-gamma release assays in the detection of latent tuberculosis infection in patients with inflammatory arthritis scheduled for anti-tumour necrosis factor treatment. *Clin Rheumatol* 2012; **31**: 785-94.

247 Miranda C, Yen-Lieberman B, Terpeluk P, et al. Reducing the rates of indeterminate results of the QuantiFERON-TB Gold In-Tube test during routine preemployment screening for latent tuberculosis infection among healthcare personnel. *Infect Control Hosp Epidemiol* 2009; **30**: 296-98.

248 Molina R, Venkatesh K, Schantz-Dunn J, et al. Comparing an Interferon Gamma Release Assay with the Tuberculin Skin Test During Pregnancy: Implications for Tuberculosis Screening During Prenatal Care. *Matern Child Health J* 2016; **20**: 1314-20.

249 Moon HW, Kim H, Hur M, et al. Latent tuberculosis infection screening for laboratory personnel using interferon-γ release assay and tuberculin skin test in Korea: an intermediate incidence setting. *J Clin Lab Anal* 2011; **25**: 382-88.

250 Moon SM, Lee SO, Choi SH, et al. Comparison of the QuantiFERON-TB Gold In-Tube test with the tuberculin skin test for detecting latent tuberculosis infection prior to hematopoietic stem cell transplantation. *Transpl Infect Dis* 2013; **15**: 104-09.

251 Moon SM, Park IA, Kim SM, et al. Living donor and recipient screening for latent tuberculosis infection by tuberculin skin test and interferon-gamma releasing assay in a country with an intermediate burden of tuberculosis. *J Infect Chemother* 2013; **19**: 1009-13.

252 Moon HH, Park SY, Kim JM, et al. Isoniazid Prophylaxis for Latent Tuberculosis Infections in Liver Transplant Recipients in a Tuberculosis-Endemic Area. *Ann Transplant* 2017; **22**: 338-45.

253 Moon HW, Gaur RL, Tien SS, et al. Evaluation of QuantiFERON-TB Gold-Plus in Health Care Workers in a Low-Incidence Setting. *J Clin Microbiol* 2017; **55**: 1650-57.

254 Moucaut A, Nienhaus A, Courtois B, et al. The effect of introducing IGRA to screen French healthcare workers for tuberculosis and potential conclusions for the work organisation. *J Occup Med Toxicol* 2013; **8**: 12.

255 Mukai S, Shigemura K, Yamamichi F, et al. Comparison of cost-effectiveness between the quantiFERON-TB Gold-In-Tube and T-Spot tests for screening health-care workers for latent tuberculosis infection. *Int J Mycobacteriol* 2017; **6**: 83-86.

256 Muñoz L, Gomila A, Casas S, et al. Immunodiagnostic Tests' Predictive Values for Progression to Tuberculosis in Transplant Recipients: A Prospective Cohort Study. *Transplant Direct* 2015; **1**: e12.

257 Nkurunungi G, Lutangira JE, Lule SA, et al. Determining Mycobacterium tuberculosis infection among BCG-immunised Ugandan children by T-SPOT.TB and tuberculin skin testing. *PLoS One* 2012; **7**: e47340.

258 Nozawa T, Mori M, Nishimura K, et al. Usefulness of two interferon-γ release assays for rheumatic disease. *Pediatr Int* 2016; **58**: 347-52.

259 Ogawa Y, Harada M, Hashimoto K, et al. Prevalence of latent tuberculosis infection and its risk factors in Japanese hemodialysis patients. *Clin Exp Nephrol* 2021; **25**: 1255-65.

260 Okada K, Mao TE, Mori T, et al. Performance of an interferon-gamma release assay for diagnosing latent tuberculosis infection in children. *Epidemiol Infect* 2008; **136**: 1179-87.

261 Oliveira S, Trajman A, Paniago AMM, et al. Frequency of indeterminate results from an interferon-gamma release assay among HIV-infected individuals. *J Bras Pneumol* 2017; **43**: 215-18.

262 Pai M, Gokhale K, Joshi R, et al. Mycobacterium tuberculosis infection in health care workers in rural India: comparison of a whole-blood interferon gamma assay with tuberculin skin testing. *JAMA* 2005; **293**: 2746-55.

263 Pai M, Joshi R, Dogra S, et al. T-cell assay conversions and reversions among household contacts of tuberculosis patients in rural India. *Int J Tuberc Lung Dis* 2009; **13**: 84-92.

264 Paluch-Oleś J, Magryś A, Kozioł-Montewka M, et al. Identification of latent tuberculosis infection in rheumatic patients under consideration for treatment with anti-TNF-α agents. *Arch Med Sci* 2013; **9**: 112-17.

265 Papay P, Eser A, Winkler S, et al. Predictors of indeterminate IFN-γ release assay in screening for latent TB in inflammatory bowel diseases. *Eur J Clin Invest* 2011; **41**: 1071-76.

266 Pareek M, Bond M, Shorey J, et al. Community-based evaluation of immigrant tuberculosis screening using interferon γ release assays and tuberculin skin testing: observational study and economic analysis. *Thorax* 2013; **68**: 230-39.

267 Park JH, Seo GY, Lee JS, et al. Positive conversion of tuberculin skin test and performance of interferon release assay to detect hidden tuberculosis infection during anti-tumor necrosis factor agent trial. *J Rheumatol* 2009; **36**: 2158-63.

268 Park HY, Jeon K, Suh GY, et al. Interferon-γ release assay for tuberculosis screening of healthcare workers at a Korean tertiary hospital. *Scand J Infect Dis* 2010; **42**: 943-45.

269 Park JH, Choi EJ, Park HS, et al. Treatment of Latent Tuberculosis Infection Based on the Interferon-γ Release Assay in Allogeneic Stem Cell Transplant Recipients. *Clin Infect Dis* 2020; **71**: 1977-79.

270 Parrella R, Esposito V, Onofrio M, et al. Interferon gamma release assays and tubercolin skin test performance in different settings of HIV immunodeficiency. *In Vivo* 2015; **29**: 137-40.

271 Passalent L, Khan K, Richardson R, et al. Detecting latent tuberculosis infection in hemodialysis patients: a head-to-head comparison of the T-SPOT.TB test, tuberculin skin test, and an expert physician panel. *Clin J Am Soc Nephrol* 2007; **2**: 68-73.

272 Pavić I, Topić RZ, Raos M, et al. Interferon-γ release assay for the diagnosis of latent tuberculosis in children younger than 5 years of age. *Pediatr Infect Dis J* 2011; **30**: 866-70.

273 Pavić I, Katalinić-Janković V, Čepin-Bogović J, et al. Discordance Between Tuberculin Skin Test and Interferon-γ Release Assay in Children Younger Than 5 Years Who Have Been Vaccinated With Bacillus Calmette-Guérin. *Lab Med* 2015; **46**: 200-06.

274 Perez-Porcuna TM, Pereira-da-Silva HD, Ascaso C, et al. Prevalence and Diagnosis of Latent Tuberculosis Infection in Young Children in the Absence of a Gold Standard. *PLoS One* 2016; **11**: e0164181.

275 Perifanou D, Zoe D, Petinaki E, et al. Screening for Latent Tuberculosis Infection in Patients with Autoimmune Diseases Before Initiating TNF-α Inhibitors Therapy. *Mater Sociomed* 2018; **30**: 32-37.

276 Perry S, Sanchez L, Yang S, et al. Reproducibility of QuantiFERON-TB gold in-tube assay. *Clin Vaccine Immunol* 2008; **15**: 425-32.

277 Person AK, Goswami ND, Bissette DJ, et al. Pairing QuantiFERON gold in-tube with opt-out HIV testing in a tuberculosis contact investigation in the Southeastern United States. *AIDS Patient Care STDS* 2010; **24**: 539-43.

278 Petrucci R, Abu Amer N, Gurgel RQ, et al. Interferon gamma, interferon-gamma-induced-protein 10, and tuberculin responses of children at high risk of tuberculosis infection. *Pediatr Infect Dis J* 2008; **27**: 1073-77.

279 Piana F, Codecasa LR, Cavallerio P, et al. Use of a T-cell-based test for detection of tuberculosis infection among immunocompromised patients. *Eur Respir J* 2006; **28**: 31-34.

280 Ponce de Leon D, Acevedo-Vasquez E, Alvizuri S, et al. Comparison of an interferon-gamma assay with tuberculin skin testing for detection of tuberculosis (TB) infection in patients with rheumatoid arthritis in a TB-endemic population. *J Rheumatol* 2008; **35**: 776-81.

281 Porsa E, Cheng L, Graviss EA. Comparison of an ESAT-6/CFP-10 peptide-based enzyme-linked immunospot assay to a tuberculin skin test for screening of a population at moderate risk of contracting tuberculosis. *Clin Vaccine Immunol* 2007; **14**: 714-19.

282 Pratt A, Nicholl K, Kay L. Use of the QuantiFERON TB Gold test as part of a screening programme in patients with RA under consideration for treatment with anti-TNF-alpha agents: the Newcastle (UK) experience. *Rheumatology (Oxford)* 2007; **46**: 1035-36.

283 Prignano F, Bartoloni A, Bartalesi F, et al. Latent tuberculosis infection in psoriasis and other dermatological immunomediated diseases: a combined approach by QuantiFERON-TB Gold and tuberculin skin tests. *Int J Dermatol* 2014; **53**: e372-74.

284 Primaturia C, Reniarti L, Nataprawira H. Comparison between the Interferon γ Release Assay-QuantiFERON Gold Plus (QFT-Plus)-and Tuberculin Skin Test (TST) in the Detection of Tuberculosis Infection in Immunocompromised Children. *Pulm Med* 2020; **2020**: 7159485.

285 Pullar ND, Steinum H, Tonby K, et al. Low prevalence of positive interferon-gamma tests in HIV-positive long-term immigrants in Norway. *Int J Tuberc Lung Dis* 2014; **18**: 180-87.

286 Qumseya BJ, Ananthakrishnan AN, Skaros S, et al. QuantiFERON TB gold testing for tuberculosis screening in an inflammatory bowel disease cohort in the United States. *Inflamm Bowel Dis* 2011; **17**: 77-83.

287 Rafiza S, Rampal KG, Tahir A. Prevalence and risk factors of latent tuberculosis infection among health care workers in Malaysia. *BMC Infect Dis* 2011; **11**: 19.

288 Ramos JM, Robledano C, Masiá M, et al. Contribution of interferon gamma release assays testing to the diagnosis of latent tuberculosis infection in HIV-infected patients: a comparison of QuantiFERON-TB Gold In Tube, T-SPOT.TB and tuberculin skin test. *BMC Infect Dis* 2012; **12**: 169.

289 Ramos JM, Masiá M, Rodríguez JC, et al. Negative effect of immunosuppressive therapy in the performance of the QuantiFERON gold in-tube

test in patients with immune-mediated inflammatory diseases. *Clin Exp Med* 2013; **13**: 177-86.

290 Ranaivomanana P, Raharimanga V, Dubois PM, et al. Study of the BCG Vaccine-Induced Cellular Immune Response in Schoolchildren in Antananarivo, Madagascar. *PLoS One* 2015; **10**: e0127590.

291 Rangaka MX, Wilkinson RJ, Boulle A, et al. Isoniazid plus antiretroviral therapy to prevent tuberculosis: a randomised double-blind, placebo-controlled trial. *Lancet* 2014; **384**: 682-90.

292 Rangaka MX, Wilkinson KA, Seldon R, et al. Effect of HIV-1 infection on T-Cell-based and skin test detection of tuberculosis infection. *Am J Respir Crit Care Med* 2007; **175**: 514-20.

293 Ribeiro-Rodrigues R, Kim S, Coelho da Silva FD, et al. Discordance of tuberculin skin test and interferon gamma release assay in recently exposed household contacts of pulmonary TB cases in Brazil. *PLoS One* 2014; **9**: e96564.

294 Richeldi L, Bergamini BM, Vaienti F. Prior tuberculin skin testing does not boost QuantiFERON-TB results in paediatric contacts. *Eur Respir J* 2008; **32**: 524-25.

295 Richeldi L, Losi M, D'Amico R, et al. Performance of tests for latent tuberculosis in different groups of immunocompromised patients. *Chest* 2009; **136**: 198-204.

296 Ringshausen FC, Schlösser S, Nienhaus A, et al. In-hospital contact investigation among health care workers after exposure to smear-negative tuberculosis. *J Occup Med Toxicol* 2009; **4**: 11.

297 Ringshausen FC, Nienhaus A, Schablon A, et al. Predictors of persistently positive Mycobacterium-tuberculosis-specific interferon-gamma responses in the serial testing of health care workers. *BMC Infect Dis* 2010; **10**: 220.

298 Rivas I, Latorre I, Sanvisens A, et al. Prospective evaluation of latent tuberculosis with interferon-gamma release assays in drug and alcohol abusers. *Epidemiol Infect* 2009; **137**: 1342-47.

299 Romanowski K, Rose C, Cook VJ, et al. Effectiveness of Latent TB Screening and Treatment in People Initiating Dialysis in British Columbia, Canada. *Can J Kidney Health Dis* 2020; **7**: 2054358120937104.

300 Rose W, Read SE, Bitnun A, et al. Relating Tuberculosis (TB) Contact Characteristics to QuantiFERON-TB-Gold and Tuberculin Skin Test Results in the Toronto Pediatric TB Clinic. *J Pediatric Infect Dis Soc* 2015; **4**: 96-103.

301 Roth PJ, Grim SA, Gallitano S, et al. Serial testing for latent tuberculosis infection in transplant candidates: a retrospective review. *Transpl Infect Dis* 2016; **18**: 14-21.

302 Rousset S, Treiner E, Moulis G, et al. High rate of indeterminate results of the QuantiFERON-TB Gold in-tube test, third generation, in patients with systemic vasculitis. *Rheumatology (Oxford)* 2020; **59**: 1006-10.

303 Ruhwald M, Petersen J, Kofoed K, et al. Improving T-cell assays for the diagnosis of latent TB infection: potential of a diagnostic test based on IP-10. *PLoS One* 2008; **3**: e2858.

304 Ruhwald M, Aggerbeck H, Gallardo RV, et al. Safety and efficacy of the C-Tb skin test to diagnose Mycobacterium tuberculosis infection, compared with an interferon γ release assay and the tuberculin skin test: a phase 3, double-blind, randomised, controlled trial. *Lancet Respir*

Med 2017; **5**: 259-68.

305 Rutherford ME, Nataprawira M, Yulita I, et al. QuantiFERON®-TB Gold In-Tube assay vs. tuberculin skin test in Indonesian children living with a tuberculosis case. *Int J Tuberc Lung Dis* 2012; **16**: 496-502.

306 Ryu MR, Park MS, Cho EH, et al. Comparative Evaluation of QuantiFERON-TB Gold In-Tube and QuantiFERON-TB Gold Plus in Diagnosis of Latent Tuberculosis Infection in Immunocompromised Patients. *J Clin Microbiol* 2018; **56**: e00438-18.

307 Sabri A, Quistrebert J, Naji Amrani H, et al. Prevalence and risk factors for latent tuberculosis infection among healthcare workers in Morocco. *PLoS One* 2019; **14**: e0221081.

308 Said K, Hella J, Ruzegea M, et al. Immunologic-based Diagnosis of Latent Tuberculosis Among Children Younger Than 5 Years of Age Exposed and Unexposed to Tuberculosis in Tanzania. *Pediatr Infect Dis J* 2019; **38**: 333-39.

309 Saidenberg-Kermanac'h N, Semerano L, Naccache JM, et al. Screening for latent tuberculosis in anti-TNF-α candidate patients in a high tuberculosis incidence setting. *Int J Tuberc Lung Dis* 2012; **16**: 1307-14.

310 Sandhu P, Taylor C, Miller RF, Post FA. Implementation of routine interferon-gamma release assay testing in a South London HIV cohort. *Int J STD AIDS* 2020; **31**: 264-67.

311 Santin M, Casas S, Saumoy M, et al. Detection of latent tuberculosis by the tuberculin skin test and a whole-blood interferon-γ release assay, and the development of active tuberculosis in HIV-seropositive persons. *Diagn Microbiol Infect Dis* 2011; **69**: 59-65.

312 Sauzullo I, Mengoni F, Scrivo R, et al. Evaluation of QuantiFERON-TB Gold In-Tube in human immunodeficiency virus infection and in patient candidates for anti-tumour necrosis factor-alpha treatment. *Int J Tuberc Lung Dis* 2010; **14**: 834-40.

313 Sauzullo I, Mengoni F, Marocco R, et al. Interferon-γ release assay for tuberculosis in patients with psoriasis treated with tumour necrosis factor antagonists: in vivo and in vitro analysis. *Br J Dermatol* 2013; **169**: 1133-40.

314 Sauzullo I, Mastroianni CM, Mengoni F, et al. Long-term IFN-γ and IL-2 response for detection of latent tuberculosis infection in healthcare workers with discordant immunologic results. *J Immunol Methods* 2014; **414**: 51-57.

315 Savaj S, Savoj J, Ranjbar M, et al. Interferon-gamma release assay agreement with tuberculin skin test in pretransplant screening for latent tuberculosis in a high-prevalence country. *Iran J Kidney Dis* 2014; **8**: 329-32.

316 Sayyahfar S, Mahdavi M, Seyedi Arani S, et al. Comparison of tuberculin skin test and interferon gamma release assay in pediatric candidates of heart transplantation and a 2-year follow-up. *Transpl Infect Dis* 2020; **22**: e13268.

317 Schablon A, Harling M, Diel R, Nienhaus A. Risk of latent TB infection in individuals employed in the healthcare sector in Germany: a multicentre prevalence study. *BMC Infect Dis* 2010; **10**: 107.

318 Aberra FN. Comparison of interferon-gamma release assay versus tuberculin skin test for tuberculosis screening in inflammatory bowel disease. *Gastroenterology* 2009; **136**: 1453-55; discussion 1455.

319 Scordo JM, Aguillón-Durán GP, Ayala D, et al. Interferon gamma release assays for detection of latent Mycobacterium tuberculosis in older Hispanic people. *Int J Infect Dis* 2021; **111**: 85-91.

320 Scrivo R, Sauzullo I, Mengoni F, et al. Serial interferon-γ release assays for screening and monitoring of tuberculosis infection during treatment with biologic agents. *Clin Rheumatol* 2012; **31**: 1567-75.

321 Scrivo R, Sauzullo I, Mengoni F, et al. Mycobacterial interferon-γ release variations during longterm treatment with tumor necrosis factor blockers: lack of correlation with clinical outcome. *J Rheumatol* 2013; **40**: 157-65.

322 Scrivo R, Molteni E, Castellani C, et al. Are interferon-gamma release assays reliable to detect tuberculosis infection in patients with rheumatoid arthritis treated with Janus kinase inhibitors. *PLoS One* 2022; **17**: e0275329.

323 Sester M, van Leth F, Bruchfeld J, et al. Risk assessment of tuberculosis in immunocompromised patients. A TBNET study. *Am J Respir Crit Care Med* 2014; **190**: 1168-76.

324 Shah M, Kasambira TS, Adrian PV, Madhi SA, et al. Longitudinal analysis of QuantiFERON-TB Gold In-Tube in children with adult household tuberculosis contact in South Africa: a prospective cohort study. *PLoS One* 2011; **6**: e26787.

325 Shanaube K, Hargreaves J, Fielding K, et al. Risk factors associated with positive QuantiFERON-TB Gold In-Tube and tuberculin skin tests results in Zambia and South Africa. *PLoS One* 2011; **6**: e18206.

326 Sharninghausen JC, Shapiro AE, Koelle DM, et al. Risk Factors for Indeterminate Outcome on Interferon Gamma Release Assay in Non-US-Born Persons Screened for Latent Tuberculosis Infection. *Open Forum Infect Dis* 2018; **5**: ofy184.

327 Shin HJ, Kim TO, Oh HJ, et al. Impact of diabetes mellitus on indeterminate results of the QuantiFERON TB Gold In-Tube test: A propensity score matching analysis. *PLoS One* 2017; **12**: e0181887.

328 Shovman O, Anouk M, Vinnitsky N, et al. QuantiFERON-TB Gold in the identification of latent tuberculosis infection in rheumatoid arthritis: a pilot study. *Int J Tuberc Lung Dis* 2009; **13**: 1427-32.

329 Shu CC, Wu VC, Yang FJ, et al. Predictors and prevalence of latent tuberculosis infection in patients receiving long-term hemodialysis and peritoneal dialysis. *PLoS One* 2012; **7**: e42592.

330 Shu CC, Wu VC, Yang FJ, et al. Dynamic changes in positive interferon-gamma release assay in a dialysis population: An observational cohort study. *J Infect* 2013; **67**: 529-35.

331 Shu CC, Hsu CL, Lee CY, et al. Comparison of the Prevalence of Latent Tuberculosis Infection among Non-Dialysis Patients with Severe Chronic Kidney Disease, Patients Receiving Dialysis, and the Dialysis-Unit Staff: A Cross-Sectional Study. *PLoS One* 2015; **10**: e0124104.

332 Shu CC, Hsu CL, Wei YF, et al. Risk of Tuberculosis Among Patients on Dialysis: The Predictive Value of Serial Interferon-Gamma Release Assay. *Medicine (Baltimore)* 2016; **95**: e3813.

333 Shu CC, Tsai MK, Lin SW, et al. Latent Tuberculosis Infection Increases in Kidney Transplantation Recipients Compared With Transplantation Candidates: A Neglected Perspective in Tuberculosis Control. *Clin Infect Dis* 2020; **71**: 914-23.

334 Silveira MBV, Ferrarini MAG, Viana PO, et al. Contribution of the interferon-gamma release assay to tuberculosis diagnosis in children and adolescents. *Int J Tuberc Lung Dis* 2018; **22**: 1172-78.

335 Simpson T, Tomaro J, Jobb C. Implementation of an interferon-gamma release assay to screen for tuberculosis in refugees and immigrants. *J*

Immigr Minor Health 2013; **15**: 686-92.

336 Slater ML, Welland G, Pai M, et al. Challenges with QuantiFERON-TB Gold assay for large-scale, routine screening of U.S. healthcare workers. *Am J Respir Crit Care Med* 2013; **188**: 1005-10.

337 Soborg B, Koch A, Thomsen VØ, et al. Ongoing tuberculosis transmission to children in Greenland. *Eur Respir J* 2010; **36**: 878-84.

338 Soborg B, Ruhwald M, Hetland ML, et al. Comparison of screening procedures for Mycobacterium tuberculosis infection among patients with inflammatory diseases. *J Rheumatol* 2009; **36**: 1876-84.

339 Sollai S, Ghetti F, Bianchi L, et al. Infectious diseases prevalence, vaccination coverage, and diagnostic challenges in a population of internationally adopted children referred to a Tertiary Care Children's Hospital from 2009 to 2015. *Medicine (Baltimore)* 2017; **96**: e6300.

340 Song SE, Yang J, Lee KS, et al. Comparison of the tuberculin skin test and interferon gamma release assay for the screening of tuberculosis in adolescents in close contact with tuberculosis TB patients. *PLoS One* 2014; **9**: e100267.

341 Sosa-Moreno A, Narita M, Spitters C, et al. A Targeted Screening Program for Latent Tuberculosis Infection Among Hematopoietic Cell Transplant Recipients. *Open Forum Infect Dis* 2020; **7**: ofaa224.

342 Sousa M, Ladeira I, Ponte A, et al. Screening for latent tuberculosis in patients with inflammatory bowel disease under antitumor necrosis factor: data from a Portuguese center. *Eur J Gastroenterol Hepatol* 2019; **31**: 1099-102.

343 Southern J, Sridhar S, Tsou CY, et al. Discordance in latent tuberculosis (TB) test results in patients with end-stage renal disease. *Public Health* 2019; **166**: 34-39.

344 Souza JM, Evangelista Mdo S, Trajman A. Added value of QuantiFERON TB-gold in-tube for detecting latent tuberculosis infection among persons living with HIV/AIDS. *Biomed Res Int* 2014; **2014**: 294963.

345 Stefan DC, Dippenaar A, Detjen AK, et al. Interferon-gamma release assays for the detection of Mycobacterium tuberculosis infection in children with cancer. *Int J Tuberc Lung Dis* 2010; **14**: 689-94.

346 Stephan C, Wolf T, Goetsch U, et al. Comparing QuantiFERON-tuberculosis gold, T-SPOT tuberculosis and tuberculin skin test in HIV-infected individuals from a low prevalence tuberculosis country. *AIDS* 2008; **22**: 2471-79.

347 Sultan B, Benn P, Mahungu T, et al. Comparison of two interferon-gamma release assays (QuantiFERON-TB Gold In-Tube and T-SPOT.TB) in testing for latent tuberculosis infection among HIV-infected adults. *Int J STD AIDS* 2013; **24**: 775-79.

348 Sun HY, Hsueh PR, Liu WC, et al. Risk of Active Tuberculosis in HIV-Infected Patients in Taiwan with Free Access to HIV Care and a Positive T-Spot.TB Test. *PLoS One* 2015; **10**: e0125260.

349 Takeda N, Nojima T, Terao C, et al. Interferon-gamma release assay for diagnosing Mycobacterium tuberculosis infections in patients with systemic lupus erythematosus. *Lupus* 2011; **20**: 792-800.

350 Talati NJ, Seybold U, Humphrey B, et al. Poor concordance between interferon-gamma release assays and tuberculin skin tests in diagnosis of latent tuberculosis infection among HIV-infected individuals. *BMC Infect Dis* 2009; **9**: 15.

351 Talebi-Taher M, Javad-Moosavi SA, Entezari AH, et al. Comparing the performance of QuantiFERON-TB Gold and Mantoux test in detecting

latent tuberculosis infection among Iranian health care workers. *Int J Occup Med Environ Health* 2011; **24**: 359-66.

352 Tanabe M, Nakamura A, Arai A, et al. The Direct Comparison of Two Interferon-gamma Release Assays in the Tuberculosis Screening of Japanese Healthcare Workers. *Intern Med* 2017; **56**: 773-79.

353 Tavast E, Tuuminen T, Pakkanen SH, et al. Immunosuppression Adversely Affects TST but Not IGRAs in Patients with Psoriasis or Inflammatory Musculoskeletal Diseases. *Int J Rheumatol* 2012; **2012**: 381929.

354 Teranishi S, Kobayashi N, Aoki A, et al. Reproducibility of the T-SPOT.TB test for screening Mycobacterium tuberculosis infection in Japan. *J Infect Chemother* 2020; **26**: 194-98.

355 Theel ES, Hilgart H, Breen-Lyles M, et al. Comparison of the QuantiFERON-TB Gold Plus and QuantiFERON-TB Gold In-Tube Interferon Gamma Release Assays in Patients at Risk for Tuberculosis and in Health Care Workers. *J Clin Microbiol* 2018; **56**: e00614-18.

356 Theodoropoulos N, Lanternier F, Rassiwala J, et al. Use of the QuantiFERON-TB Gold interferon-gamma release assay for screening transplant candidates: a single-center retrospective study. *Transpl Infect Dis* 2012; **14**: 1-8.

357 Thi AA, Abbara A, Bouri S, et al. Challenges in screening for latent tuberculosis in inflammatory bowel disease prior to biologic treatment: a UK cohort study. *Frontline Gastroenterol* 2018; **9**: 234-40.

358 Thomas TA, Mondal D, Noor Z, et al. Malnutrition and helminth infection affect performance of an interferon gamma-release assay. *Pediatrics* 2010; **126**: e1522-29.

359 Thomas B, Pugalenthi A, Patel H, et al. Concordance between tuberculin skin test and interferon-γ assay and interferon-γ response to mitogen in pediatric tuberculosis contacts. *Pediatr Pulmonol* 2011; **46**: 1225-32.

360 Tiernan JF, Gilhooley S, Jones ME, et al. Does an interferon-gamma release assay change practice in possible latent tuberculosis. *QJM* 2013; **106**: 139-46.

361 Tieu HV, Suntarattiwong P, Puthanakit T, et al. Comparing interferon-gamma release assays to tuberculin skin test in Thai children with tuberculosis exposure. *PLoS One* 2014; **9**: e105003.

362 Triverio PA, Bridevaux PO, Roux-Lombard P, et al. Interferon-gamma release assays versus tuberculin skin testing for detection of latent tuberculosis in chronic haemodialysis patients. *Nephrol Dial Transplant* 2009; **24**: 1952-56.

363 Tsou PH, Huang WC, Huang CC, et al. Quantiferon TB-Gold conversion can predict active tuberculosis development in elderly nursing home residents. *Geriatr Gerontol Int* 2015; **15**: 1179-84.

364 Tsuyuzaki M, Igari H, Okada N, et al. Variation in interferon-γ production between QFT-Plus and QFT-GIT assays in TB contact investigation. *Respir Investig* 2019; **57**: 561-65.

365 Tuuminen T, Salo E, Kotilainen H, et al. Evaluation of the filter paper IP-10 tests in school children after exposure to tuberculosis: a prospective cohort study with a 4-year follow-up. *BMJ Open* 2012; **2**: e001751.

366 Umekita K, Hashiba Y, Iwao K, et al. Human T-cell leukemia virus type 1 may invalidate T-SPOT.TB assay results in rheumatoid arthritis patients: A retrospective case-control observational study. *PLoS One* 2020; **15**: e0233159.

367 Vajravelu RK, Osterman MT, Aberra FN, et al. Indeterminate QuantiFERON-TB Gold Increases Likelihood of Inflammatory Bowel Disease Treatment Delay and Hospitalization. *Inflamm Bowel Dis* 2017; **24**: 217-26.

368 Vassilopoulos D, Tsikrika S, Hatzara C, et al. Comparison of two gamma interferon release assays and tuberculin skin testing for tuberculosis screening in a cohort of patients with rheumatic diseases starting anti-tumor necrosis factor therapy. *Clin Vaccine Immunol* 2011; **18**: 2102-08.

369 Velasco-Arnaiz E, Soriano-Arandes A, Latorre I, et al. Performance of Tuberculin Skin Tests and Interferon-γ Release Assays in Children Younger Than 5 Years. *Pediatr Infect Dis J* 2018; **37**: 1235-41.

370 Velasco-Arnaiz E, Soriano-Arandes A, Espiau M, et al. Impact of Baseline Tuberculin Skin Test and Isoniazid Chemoprophylaxis on Subsequent Quantiferon-TB Gold In-Tube Performance in Young Children Assessed After Tuberculosis Contact in Catalonia. *Pediatr Infect Dis J* 2020; **39**: e22-22e25.

371 Velasco-Arnaiz E, Batllori M, Monsonís M, et al. Host, technical, and environmental factors affecting QuantiFERON-TB Gold In-Tube performance in children below 5 years of age. *Sci Rep* 2022; **12**: 19908.

372 Venkatappa TK, Punnoose R, Katz DJ, et al. Comparing QuantiFERON-TB Gold Plus with Other Tests To Diagnose Mycobacterium tuberculosis Infection. *J Clin Microbiol* 2019; **57**: e00985-19.

373 Verhagen LM, Hermans PW, Warris A, et al. Helminths and skewed cytokine profiles increase tuberculin skin test positivity in Warao Amerindians. *Tuberculosis (Edinb)* 2012; **92**: 505-12.

374 Verhagen LM, Maes M, Villalba JA, et al. Agreement between QuantiFERON®-TB Gold In-Tube and the tuberculin skin test and predictors of positive test results in Warao Amerindian pediatric tuberculosis contacts. *BMC Infect Dis* 2014; **14**: 383.

375 Vortia E, Uko VE, Yen-Lieberman B, et al. Low Indeterminate Rates Associated With Use of the QuantiFERON-TB Gold In-Tube Test in Children With Inflammatory Bowel Disease on Long-term Infliximab. *Inflamm Bowel Dis* 2018; **24**: 877-82.

376 Wang PH, Lin CH, Chang TH, et al. Chest roentgenography is complementary to interferon-gamma release assay in latent tuberculosis infection screening of rheumatic patients. *BMC Pulm Med* 2020; **20**: 232.

377 Wassie L, Aseffa A, Abebe M, et al. Parasitic infection may be associated with discordant responses to QuantiFERON and tuberculin skin test in apparently healthy children and adolescents in a tuberculosis endemic setting, Ethiopia. *BMC Infect Dis* 2013; **13**: 265.

378 Wei Z, Yang M, Quan B, et al. Prevalence of latent tuberculosis infection among healthcare workers in China as detected by two interferon-gamma release assays. *J Hosp Infect* 2013; **84**: 323-25.

379 Weinberg A, Aaron L, Montepiedra G, et al. Effects of Pregnancy and Isoniazid Preventive Therapy on Mycobacterium tuberculosis Interferon Gamma Response Assays in Women With HIV. *Clin Infect Dis* 2021; **73**: e3555-3555e3562.

380 Weinfurter P, Blumberg HM, Goldbaum G, et al. Predictors of discordant tuberculin skin test and QuantiFERON®-TB Gold In-Tube results in various high-risk groups. *Int J Tuberc Lung Dis* 2011; **15**: 1056-61.

381 Wendorf KA, Lowenthal P, Feraud J, et al. Interferon-γ Release Assays for Tuberculosis Infection Diagnosis in Refugees <5 Years Old. *Pediatrics* 2020; **146**: e20200715.

382 Wigg AJ, Narayana SK, Anwar S, et al. High rates of indeterminate interferon-gamma release assays for the diagnosis of latent tuberculosis infection in liver transplantation candidates. *Transpl Infect Dis* 2019; **21**: e13087.

383 Wikell A, Jonsson J, Dyrdak R, et al. The Impact of Borderline Quantiferon-TB Gold Plus Results for Latent Tuberculosis Screening under Routine Conditions in a Low-Endemicity Setting. *J Clin Microbiol* 2021; **59**: e0137021.

384 Wilder-Smith A, Foo W, Earnest A, et al. High risk of Mycobacterium tuberculosis infection during the Hajj pilgrimage. *Trop Med Int Health* 2005; **10**: 336-39.

385 Winthrop KL, Nyendak M, Calvet H, et al. Interferon-gamma release assays for diagnosing mycobacterium tuberculosis infection in renal dialysis patients. *Clin J Am Soc Nephrol* 2008; **3**: 1357-63.

386 Wong SH, Ip M, Tang W, et al. Performance of interferon-gamma release assay for tuberculosis screening in inflammatory bowel disease patients. *Inflamm Bowel Dis* 2014; **20**: 2067-72.

387 Wu CY, Chiu HY, Tsai TF. The seroconversion rate of QuantiFERON-TB Gold In-Tube test in psoriatic patients receiving secukinumab and ixekizumab, the anti-interleukin-17A monoclonal antibodies. *PLoS One* 2019; **14**: e0225112.

388 Xu Y, Yang Q, Zhou J, et al. Comparison of QuantiFERON-TB Gold In-Tube and QuantiFERON-TB Gold-Plus in the Diagnosis of Mycobacterium tuberculosis Infections in Immunocompromised Patients: a Real-World Study. *Microbiol Spectr* 2022; **10**: e0187021.

389 Yang CH, Chan PC, Liao ST, et al. Strategy to better select HIV-infected individuals for latent TB treatment in BCG-vaccinated population. *PLoS One* 2013; **8**: e73069.

390 Yap P, Tan K, Lim WY, et al. Prevalence of and risk factors associated with latent tuberculosis in Singapore: A cross-sectional survey. *Int J Infect Dis* 2018; **72**: 55-62.

391 Yassin MA, Petrucci R, Garie KT, et al. Can interferon-gamma or interferon-gamma-induced-protein-10 differentiate tuberculosis infection and disease in children of high endemic areas. *PLoS One* 2011; **6**: e23733.

392 Yassin MA, Petrucci R, Garie KT, et al. Use of tuberculin skin test, IFN-γ release assays and IFN-γ-induced protein-10 to identify children with TB infection. *Eur Respir J* 2013; **41**: 644-48.

393 Yilmaz N, Zehra Aydin S, Inanc N, et al. Comparison of QuantiFERON-TB Gold test and tuberculin skin test for the identification of latent Mycobacterium tuberculosis infection in lupus patients. *Lupus* 2012; **21**: 491-95.

394 Yu L, Mo P, Wei Z, et al. Development and evaluation of a new interferon-gamma release assay for the diagnosis of tuberculosis infection in HIV-infected individuals in China. *Infect Dis (Lond)* 2015; **47**: 237-43.

395 Yun JW, Chung HS, Koh WJ, et al. Significant reduction in rate of indeterminate results of the QuantiFERON-TB Gold In-Tube test by shortening incubation delay. *J Clin Microbiol* 2014; **52**: 90-94.

396 Yun KW, Kim YK, Kim HR, et al. Usefulness of interferon-γ release assay for the diagnosis of latent tuberculosis infection in young children. *Korean J Pediatr* 2016; **59**: 256-61.

397 Zellweger JP, Sotgiu G, Block M, et al. Risk Assessment of Tuberculosis in Contacts by IFN-γ Release Assays. A Tuberculosis Network

European Trials Group Study. *Am J Respir Crit Care Med* 2015; **191**: 1176-84.

398 Zhang H, Li X, Xin H, et al. Association of Body Mass Index with the Tuberculosis Infection: a Population-based Study among 17796 Adults in Rural China. *Sci Rep* 2017; **7**: 41933.

399 Zhang H, Xin H, Wang D, et al. Serial testing of Mycobacterium tuberculosis infection in Chinese village doctors by QuantiFERON-TB Gold Plus, QuantiFERON-TB Gold in-Tube and T-SPOT.TB. *J Infect* 2019; **78**: 305-10.

400 Zhang L, Zhang J, Yang S, et al. Performance of LTBI Screening in Patients with Rheumatic Diseases Using Two Different Interferon-Gamma Releasing Assays. *Front Biosci (Landmark Ed)* 2022; **27**: 282.

401 Zhao J, Wang Y, Wang H, et al. Low agreement between the T-SPOT®.TB assay and the tuberculin skin test among college students in China. *Int J Tuberc Lung Dis* 2011; **15**: 134-36.

402 Zrinski Topić R, Zoričić-Letoja I, Pavić I, et al. Indeterminate results of QuantiFERON-TB Gold In-Tube assay in nonimmunosuppressed children. *Arch Med Res* 2011; **42**: 138-43.

403 Zwerling A, Pai M, Michael JS, Christopher DJ. Serial testing using interferon-γ release assays in nursing students in India. *Eur Respir J* 2014; **44**: 257-60.

Pre-specified analysis plan

Indeterminate Events in Interferon-Gamma Release Assays in the Screening of Latent Tuberculosis Infection: A Systematic Review and Meta-Analysis Protocol

Background

Testing people for latent TB infection (LTBI) and providing effective treatment are critical for the elimination of tuberculosis (TB) disease (1). Given the absence of a gold-standard test to detect LTBI, two immune-based tests, interferon-γ release assay (IGRA) and tuberculin skin test (TST), are currently used (2, 3). Both types of commercially available IGRA cause mitogen stimulation of cells in parallel to the specific antigen stimulation to measure the ability of the harvested cells to produce interferon-γ. If an individual does not respond sufficiently to either specific antigens or the mitogen control, they are deemed indeterminate (4).

Previous meta-analyses have evaluated the indeterminate rate of IGRA in the diagnosis of active TB(5-7). One study focused on the indeterminate results for the diagnosis of tuberculosis in children but included both active TB and LTBI(8). Three studies evaluated the indeterminate rate of IGRA in the screening of LTBI(9-11), however, two focused on patients with inflammatory bowel disease and one on transplant candidates.

Objective

To determine the indeterminate rate of IGRA in the screening of LTBI.

Criteria for considering studies for this review

Participants (P)

*General population or healthy people

*and High risk of LTBI:

(1) Recent contacts, both close/household and casual contacts;

(2) Immunocompromised patients, people living with HIV, chronic renal failure and/or haemodialysis, transplant recipients (organ or hematopoietic stem cell), drug and/or alcohol abusers, cancer (all types), malnourished (BMI ≤ 18.5 kg/m), silicosis;

(3) With the possibility of contact, occupational risk (e.g. healthcare worker), immigrants or refugees, army personnel;

(4) With the possibility of immunosuppression, immune-mediated inflammatory diseases (IMID), prisoners, children, nursing home residents, homeless.

*screened fro LTBI with IGRA testing.

*without age or sex restrictions.

Intervention and control (I+C)

Intervention: IGRA testing.

Control: None.

Outcome (O)

indeterminate events were reported.

Studies (s)

Original research articles with a cross-sectional, cohort design or randomized controlled trials.

Search methods for identification of studies

A comprehensive search strategy will be developed, including various terms, for identification of published and unpublished articles without start date or language restrictions. We will search the following electronic databases for primary studies: EMBASE, PubMed, and the Cochrane Library.

Data collection and analysis

Selection of studies

Two authors will independently screen the search outputs for potentially eligible studies, compare their results and resolve disagreements by a third author discussion and consensus. The two authors will then independently go through the full text of all potentially eligible studies to assess whether the studies meet the inclusion criteria defined by the study design, setting and outcomes. Discrepancies in the list of eligible studies between the two authors will be resolved through a third author discussion and consensus.

Data extraction

Data extraction will be performed by two investigators based on the predefined inclusion and exclusion criteria, and a data extraction sheet will be developed based on the Cochrane Handbook for Systematic Reviews of Intervention (12). The form will include a fixed set of fields: first author's name, year of publication, title, study area, study design, timing of data collection (prospective or retrospective), investigated population, patient demographics, IGRA type and manufacturer, number of patients screened, and number of patients with indeterminate results. Two investigators will independently extract data from individual studies. The third investigator cross-check extracted data. Disagreements were resolved through consensus.

Data synthesis

The data will be collected using Microsoft Excel (Microsoft Corp. Albuquerque, NM, USA). A meta-analysis will be conducted using the "meta" package in R statistical software version 3.4.3 (Schwarzer, 2007; Team, 2017) .

We plan to use a random-effects model to calculate pooled results and a 95% confidence interval (CI). The Q and I^2 tests will be used to evaluate the statistical heterogeneity among studies. All p values will be two-sided. A p value of < 0.05 will be considered statistically significant. "Peters" will be set as a parameter in publication bias detection, a linear regression test of funnel plot asymmetry. Trim and fill method will be used to adjust the publication bias.

Discussion

The overall indeterminate rate of IGRA at screening of LTBI remains unclear. Three studies evaluated the indeterminate rate of IGRA in the screening of LTBI(9-11), however, two focused on patients with inflammatory bowel disease and one on transplant candidates. To the best of our knowledge, this study will be the first to systematically assess the indeterminate rate of IGRA in the screening for LTBI. We believe that our outcomes will have significantly importance for clinical practice.

Ethics and dissemination

Given that this is a systematic review and mate-analysis that will use peer-reviewed, published and publicly available anonymized data, ethical approval of this protocol is not required. This review will be reported as much as possible in conformance with the PRISMA statement (13).

References

1. Rangaka MX, Cavalcante SC, Marais BJ, Thim S, Martinson NA, Swaminathan S, et al. Controlling the seedbeds of tuberculosis: diagnosis and treatment of tuberculosis infection. *Lancet* (2015) 386(10010):2344-53. doi: 10.1016/S0140-6736(15)00323-2
2. Campbell JR, Chen W, Johnston J, Cook V, Elwood K, Krot J, et al. Latent tuberculosis infection screening in immigrants to low-incidence countries: a meta-analysis. *Mol Diagn Ther* (2015) 19(2):107-17. doi: 10.1007/s40291-015-0135-6
3. Park CH, Park JH, Jung YS. Impact of Immunosuppressive Therapy on the Performance of Latent Tuberculosis Screening Tests in Patients with Inflammatory Bowel Disease: A Systematic Review and Meta-Analysis. *J Pers Med* (2022) 12(3). doi: 10.3390/jpm12030507
4. Brown J, Kumar K, Reading J, Harvey J, Murthy S, Capocci S, et al. Frequency and significance of indeterminate and borderline Quantiferon Gold TB IGRA results. *Eur Respir J* (2017) 50(4). doi: 10.1183/13993003.01267-2017
5. Diel R, Loddenkemper R, Nienhaus A. Evidence-based comparison of commercial interferon-gamma release assays for detecting active TB: a metaanalysis. Chest (2010) 137(4):952-68. doi: 10.1378/chest.09-2350
6. Huo ZY, Peng L. Accuracy of the interferon-γ release assay for the diagnosis of active tuberculosis among HIV-seropositive individuals: a systematic review and meta-analysis. BMC Infect Dis (2016) 16:350. doi: 10.1186/s12879-016-1687-8
7. Santin M, Muñoz L, Rigau D. Interferon-γ release assays for the diagnosis of tuberculosis and tuberculosis infection in HIV-infected adults: a systematic review and meta-analysis. PLoS One (2012) 7(3):e32482. doi: 10.1371/journal.pone.0032482
8. Meier NR, Volken T, Geiger M, Heininger U, Tebruegge M, Ritz N. Risk Factors for Indeterminate Interferon-Gamma Release Assay for the Diagnosis of Tuberculosis in Children-A Systematic Review and Meta-Analysis. Front Pediatr (2019) 7:208. doi: 10.3389/fped.2019.00208
9. Shahidi N, Fu YT, Qian H, Bressler B. Performance of interferon-gamma release assays in patients with inflammatory bowel disease: a systematic review and meta-analysis. Inflamm Bowel Dis (2012) 18(11):2034-42. doi: 10.1002/ibd.22901
10. Rahimifard N, Mahmoudi S, Mamishi S, Pourakbari B. Prevalence of latent tuberculosis infection in transplant candidates: A systematic review and meta-analysis. Microb Pathog (2018) 125:401-410. doi: 10.1016/j.micpath.2018.09.040

11. Park CH, Park JH, Jung YS. Impact of Immunosuppressive Therapy on the Performance of Latent Tuberculosis Screening Tests in Patients with Inflammatory Bowel Disease: A Systematic Review and Meta-Analysis. J Pers Med (2022) 12(3). doi: 10.3390/jpm12030507

12. Higgins JPT, Deeks JJ. Chapter 7: Selecting studies and collecting data. Cochrane Handbook for Systematic Reviews of Interventions Version 5.1.0. The Cochrane Collaboration (2011). www.cochrane-handbook. org.

13. Liberati A, Altman DG, Tetzlaff J, Mulrow C, Gøtzsche PC, Ioannidis JP, et al. The PRISMA statement for reporting systematic reviews and meta-analyses of studies that evaluate healthcare interventions: explanation and elaboration. *BMJ* (2009) 339:b2700. doi: 10.1136/bmj.b2700
